# Optimal frequency of scans for patients on cancer therapies: A population kinetics assessment

**DOI:** 10.1002/cam4.2571

**Published:** 2019-09-27

**Authors:** David J. Stewart, David B. Macdonald, Arif A. Awan, Kednapa Thavorn

**Affiliations:** ^1^ University of Ottawa Ottawa ON Canada

**Keywords:** population kinetics, scan frequency

## Abstract

**Background:**

Optimal frequency of follow‐up scans for patients receiving systemic therapies is poorly defined. Progression‐free survival (PFS) generally follows first‐order kinetics. We used exponential decay nonlinear regression analysis to calculate half‐lives for 887 published PFS curves.

**Method:**

We used the Excel formula x = EXP(‐*t*_n_*0.693/*t*_1/2_) to calculate proportion of residual patients remaining progression‐free at different times, where *t*_n_ is the interval in weeks between scans (eg, 6 weeks), * indicates multiplication, 0.693 is the natural logarithm of 2, and *t*_1/2_ is the PFS half‐life in weeks.

**Results:**

Proportion of residual patients predicted to remain progression‐free at each subsequent scan varied with scan intervals and regimen PFS half‐life. For example, with a 4‐month half‐life (17.3 weeks) and scans every 6 weeks, 21% of patients would progress by the first scan, 21% of the *remaining* patients would progress by the second scan at 12 weeks, etc With 2, 6‐ and 12‐month half‐lives (for example), the proportion of *remaining* patients progressing at each subsequent scan if repeated every 3 weeks would be 21%, 8% and 4%, respectively, while with scans every 12 weeks it would be 62%, 27% and 15%, respectively. Furthermore, optimal scan frequency can be calculated for populations comprised of distinct rapidly and slowly progressing subpopulations, as well as with convex curves arising from treatment breaks, where optimal scan frequency may differ during therapy administration vs during more rapid progression after therapy interruption.

**Conclusions:**

A population kinetics approach permits a regimen‐ and tumor‐specific determination of optimal scan frequency for patients on systemic therapies.

## Graphical abstract

Determination of progression‐free survival half‐lives using exponential decay nonlinear regression analysis of PFS curves permits calculation of proportion of remaining patients who would progress within a given time‐period after a follow‐up scan. This can help guide decisions on how frequently subsequent follow‐up scans should be performed.

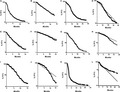

## 1 INTRODUCTION

Systemic therapies prolong median survival for most incurable malignancies. However, time to tumor progression varies substantially across patients, from weeks to years. For different therapy and tumor types, clinical trials report the median time to tumor progression across a population.

While there are many publications on optimal frequency of follow‐up scans after therapies given with curative intent, there are far fewer on optimal frequency of scans for patients with incurable malignancies. In clinical trials, patients have baseline assessments and then have periodic follow‐up scans to assess response and tumor progression. Typically, in trials, the frequency of follow‐up scans is every 6‐8 weeks or every two cycles of therapy, although this varies with tumor and treatment type. In standard clinical practice there is substantial variability across tumor types, therapies, and physician practices with respect to follow‐up scan frequency, and optimal scan frequency is uncertain.

In PubMed and Google searches, we could only find a few consensus statements on this topic rather than evidence‐based recommendations. For example, imaging guidelines from the US National Comprehensive Cancer Network (NCCN) Imaging Appropriate User Criteria (AUC^TM^)^1^ do not provide suggestions for some gastrointestinal malignancies. For metastatic breast cancer, they recommend scans every 2‐6 months for treatment with endocrine therapy and every 2‐4 cycles for treatment with cytotoxic therapy, with the stipulation that optimal frequency is uncertain. They recommend scans every 2‐4 cycles or every 6‐12 weeks (with actual scan frequency within these guidelines being a “clinical decision”) for non‐small cell lung cancer, every 2‐3 cycles during treatment and at “every subsequent follow‐up visit” for small cell lung cancer, and every 6‐16 weeks (based on “physician discretion and patient clinical status”) for kidney cancer.

For metastatic breast cancer, a survey of Italian medical oncologists found no consensus on optimal frequency of follow‐up scans, with approximately the same number of oncologists repeating scans every 3 months vs every 6 months,^2^ while European School of Oncology guidelines recommended assessment of response 2‐3 months after initiation of therapy, then every 2‐4 months for endocrine therapy and every 2‐4 cycles of chemotherapy “depending on the dynamics of the disease, the location and extent of metastatic involvement, and type of treatment.”^3^ A report based on a review of patients with metastatic breast cancer in a SEER Medicare database also noted that the optimal frequency of follow‐up scans was unknown, but the authors defined more than four scans in a 12 month period as being “extreme,” and noted that 37.6% of women were in this “extreme” group.^4^

For metastatic pancreatic cancer, an American Society of Clinical Oncology expert panel noted the absence of evidence on optimal scan frequency, but recommended a single scan 2‐3 months following initiation of therapy, then further follow‐up by clinical status alone.^5^

For genitourinary tumors, a consensus meeting of the Association of Urological Oncology of the German Cancer Society acknowledged the lack of firm data, and recommended follow‐up scans every 8 weeks in treatment of metastatic renal cell carcinoma, after every cycle of therapy for patients with urothelial cancers receiving potentially toxic chemotherapy, every 12 weeks for prostate cancer, and after two cycle of chemotherapy and again after completion of chemotherapy for nonseminoma testicular cancer.^6^

Overall, there is little firm evidence to guide frequency of follow‐up scans in most tumor types and there are pros and cons of doing scans more or less frequently. If done too frequently, there may have been too little change across scans to reliably permit assessment of change in tumor size. Measurement error can yield apparent changes of tumor size of greater than 10% in 10% or more patients.^7^, ^8^ One may require substantially greater growth or regression to be confident that the change is real. Furthermore, scans involve substantial radiation exposure,^9^ scan contrast can cause renal toxicity^10^ and allergic reactions,^11^ and overuse of scans would add significant economic burden in terms of cost of scans^12^ and lost days of productivity.

On the other hand, systemic therapies have potential toxicities and can be very expensive. Hence, they should be stopped if they are ineffective, and more frequent scanning may guide faster discontinuation of ineffective therapies.^2^ In addition, there are “opportunity costs”: if a patient continues ineffective therapy, they may be denied the option of changing to a potentially better alternative.

While there is uncertainty regarding optimal scan frequency, we could potentially define this better. Progression‐free survival (PFS) generally follows first‐order kinetics^13^ (see Table S1 for our definition of terms used). This permits use of exponential decay nonlinear regression analysis (EDNLRA) to calculate a PFS half‐life (time to progression or death of half the remaining patients). By determining PFS half‐life (which is similar to median PFS), one may then calculate the proportion of patients who would remain progression‐free at any particular time‐point. This could permit optimization of follow‐up scan frequency for that therapy.

If there are two distinct subpopulations with differing rates of progression, then PFS curves exhibit “2‐phase decay” on EDNLRA,^13^, ^14^, ^15^ and optimal scan timing may be relatively frequent early in follow‐up (when many rapidly progressing patients remain), then relatively less frequent, after most rapidly progressing patients have failed the therapy. Alternatively, tumor growth may be relatively slow while therapy is being administered, but faster if therapy is interrupted, so therapy interruption could impact optimal scan frequency.

In this paper, we present PFS half‐lives for several tumor therapy situations, and the proportion of remaining patients who would be expected to remain progression‐free at different subsequent time points. Our eventual objective is to follow this with a formal pharmacoeconomic assessment taking into consideration the direct and indirect costs of both scans and the therapy being monitored.

## 2 METHODS

**Figure 1:**
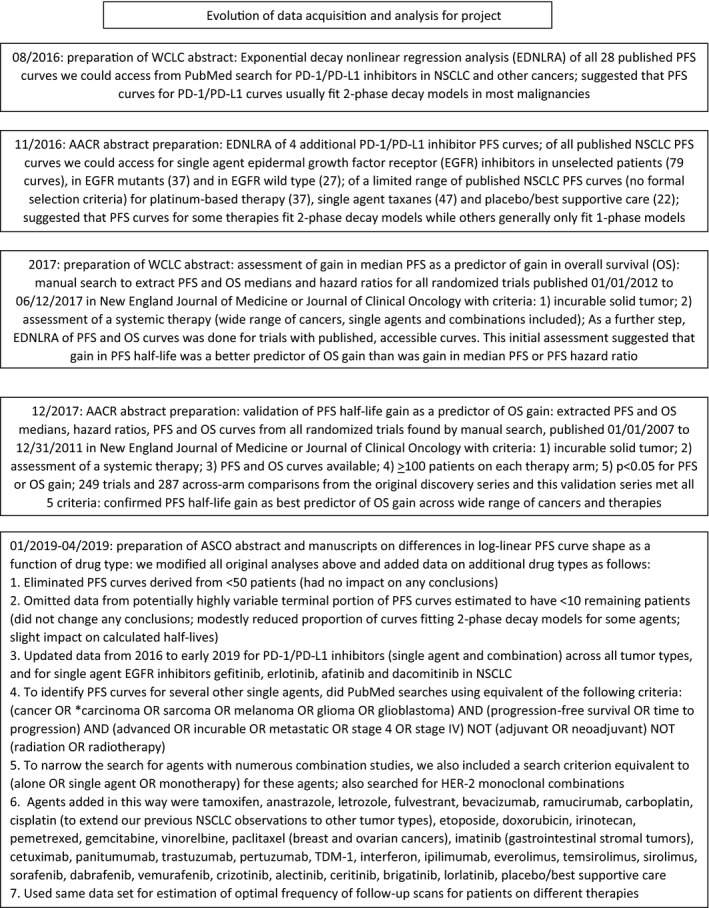
Literature search strategies: The data used for the calculations in this project came from two related projects using exponential decay nonlinear regression analysis (EDNLRA) of PFS curves, and our search strategies evolved to include larger numbers of trials as these projects developed. One project involved assessment of PFS curve shape as a function of therapy type and went from an initial assessment of PFS curve shape for PD1/PDL1 inhibitors to comparison of these to PFS curve shapes to those of a few selected other NSCLC therapies to a comparison to a wide range of additional therapies across all solid tumors. The second project initially assessed gain in PFS medians as a predictor of gain in OS medians, then evolved to an assessment of gain in PFS half‐life as a predictor of gain in OS half‐life. Ultimately, this latter project used all randomized controlled trials meeting our eligibility criteria (see figure details) published in the New England Journal of Medicine or Journal of Clinical Oncology from 1 January 2007 to 12 June 2017. PFS curves from this project were also subsequently used in our project on assessment of PFS curve shape. The only searches that did not include formal search and inclusion criteria were our initial assessment of PFS curve shapes for platinum‐based therapies and single agent taxanes in NSCLC, where PFS curves were only retrieved from a portion of all possible trials. However, observations and conclusions from these data were in agreement with data on these therapies from studies with formal search criteria included in our comparison of PFS half‐life gains to OS half‐life gains, in our assessment of single agent platinum or taxanes in other tumor types, and with a subsequent search to identify all trials of single agent cisplatin or carboplatin in NSCLC

As part of this and related population kinetics projects,^14^, ^15^, ^16^ we downloaded 887 PFS curves from 508 publications involving systemic therapies for incurable solid tumors. Our literature search approach is presented in Figure 1. Our objective was to include a wide range of drug types in a wide range of incurable solid tumors. Trials were excluded in curves derived from < 50 patients or if there were no accessible PFS curves.

We digitized these PFS curves using current or older versions of ://apps.automeris.io/wpd/. We then used GraphPad Prism 7 (GraphPad Software) for PFS half‐life calculation, using EDNLRA of the digitized data, setting the constraints Y_0_ = 100 and plateau = 0, and omitting data from potentially variable terminal curve portions estimated as having fewer than 10 remaining patients. Our broad preliminary explorations of this methodology suggested that PFS curves could generally be fit by 1‐phase decay EDNLRA models and that some could also be fit by 2‐phase decay models. If this is the case, then methods using EDNLRA to guide frequency of follow‐up scans should be potentially applicable across many tumor and therapy types, with the important factors being PFS half‐life and curve shape rather than drug or tumor type.

**Figure 2:**
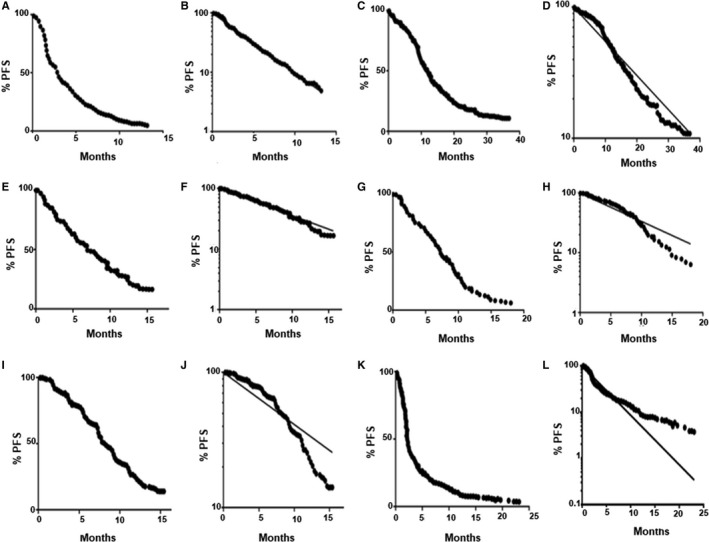
Examples of different PFS curve shapes: Kaplan‐Meier (KM) plots were reconstructed using digitized data from the original published PFS curves. Log‐linear (LL) plots were from GraphPad Prism exponential decay nonlinear regression analyses. The diagonal solid line down from 100% PFS in the LL plots is the exponential decay nonlinear regression plot for 1‐phase decay: A. 1‐phase decay (KM): docetaxel NSCLC^17^; B. 1‐phase decay (LL); C. S‐shape (KM): FOLFIRI/bevacizumab colorectal cancer^18^; D. S‐shape (LL); E. Low convexity (KM): Ceritinib NSCLC^19^; F. Low convexity (LL); G. Moderate convexity (KM): doxorubicin breast cancer^20^; H. Moderate convexity (LL); I. High convexity (KM): FOLFIRI colorectal cancer^21^; J. High convexity (LL); K. 2‐phase decay (KM):erlotinib NSCLC unselected for EGFR mutation^22^; L. 2‐phase decay (LL)

After digitizing the curves, we then created log‐linear plots of PFS curves and subjectively classified each curve based on appearance as 1‐phase (a straight line with constant slope), “S”‐shaped (undulating, but with an average slope approximating the EDNLRA regression line, and therefore very similar to 1‐phase curves), low convexity (initially following the EDNLRA regression line, but with a modest downturn at the curve's lower end, and again similar to 1‐phase curves), moderate convexity (slight early plateau above the EDNLRA regression line, followed by a modest downturn), high convexity (substantial early plateau above the EDNLRA regression line, followed by a marked downturn), or 2‐phase (initial straight line along or to the left of the EDNLRA regression line, followed by an inflection point, with deviation of the lower part of the curve to the right of the regression line). Illustrative examples of each curve type are presented in Figure 2. Note that there is substantial overlap between categories, and some curves could potentially be classified as belonging to either of two different classes (eg, either moderately convex or highly convex).

We also assessed whether the digitized data would fit EDNLRA 2‐phase decay models, with two distinct subpopulations with differing progression rates. We defined curves as fitting 2‐phase decay models if each subpopulation accounted for ≥1% of the entire population and if the half‐lives for the two subpopulations differed by a factor >2.

We then calculated the proportion of patients who would remain progression‐free at different time intervals that might be chosen for follow‐up scans. We converted half‐lives to weeks, then used the Excel formula:

$$x=\exp\left(-t_{n}*0.693/t_{1/2}\right)$$

where x is the proportion remaining progression‐free, *t*_n_ is the potential time for doing a follow‐up scan (eg, 6 weeks), * indicates multiplication, 0.693 is the natural logarithm of 2, and *t*_1/2_ is the PFS half‐life in weeks. Another way of expressing this formula (if not using Excel for the calculations) would be

$$x=2^{\wedge }\left(-t_{n}/t_{1/2}\right)$$

We then calculated

$$\%\;\text{progressing since last scan}=100-\left(x*100\right).$$

For curves with 2‐phase decay, we repeated this calculation for both the rapidly and slowly progressing subpopulations. We then calculated the proportion of all remaining patients who would be from the rapidly vs slowly progressing subpopulation at various time points of interest.

For moderate‐ or high‐convexity PFS log‐linear plots, we derived from the plot the estimated time of onset of curve convexity after therapy initiation. For high‐convexity curves, we also calculated PFS half‐lives from therapy initiation to the downward inflection point and from the downward inflection point to the end of the curve.

## 3 RESULTS

In Table 1, we present the proportion of patients remaining progression‐free at different time points after therapy initiation for populations with different PFS half‐lives. Since progression roughly follows first‐order kinetics, this means that, for example, if 80% are progression‐free at 6 weeks, then 80% of the remaining patients (or 64% of the original population) would still be progression‐free after a further 6 weeks, etc.

**Table 1:** For different progression‐free survival (PFS) half‐lives, proportion of remaining patients who would have progressed by the next scan for different time intervals between scans

PFS half‐life (mo)^a^	PFS half‐life (wk)	Time (wk) since last scan							
		3 wk	6 wk	9 wk	12 wk	18 wk	24 wk	52 wk	104 wk
		Percent of remaining patients progressing since prior scan							
1	4.3	38	62	76	85	94	98	100	100
1.5	6.5	27	47	62	72	85	92	100	100
2	8.7	21	38	51	62	76	85	98	100
2.5	10.8	17	32	44	54	68	78	96	100
3	13.0	15	27	38	47	62	72	94	100
3.5	15.2	13	24	34	42	56	67	91	99
4	17.3	11	21	30	38	51	62	87	98
5	21.7	9	17	25	32	44	54	81	96
6	26.0	8	15	21	27	38	47	75	94
7	30.3	7	13	19	24	34	42	70	91
8	34.7	6	11	16	21	30	38	65	87
9	39.0	5	10	15	19	27	35	60	84
10	43.3	5	9	13	17	25	32	56	81
12	52.0	4	8	11	15	21	27	50	75
14	60.7	3	7	10	13	19	24	45	70
16	69.3	3	6	9	11	16	21	41	65
18	78.0	3	5	8	10	15	19	37	60
20	86.7	2	5	7	9	13	17	34	56
25	108.3	2	4	6	7	11	14	28	49
30	130.0	2	3	5	6	9	12	24	43
35	151.6	1	3	4	5	8	10	21	38
40	173.3	1	2	4	5	7	9	19	34
48	208.0	1	2	3	4	6	8	16	29

a PFS half‐lives correlate strongly with and are generally similar to medians. For calculations of impact of scan frequency, half‐lives were converted from months to weeks.

Table S2 presents PFS half‐lives, PFS curve shape and references for each of the 887 PFS curves used. Note that this same supplementary table is also included in our separate manuscript on the use of population kinetics to assess the relationship between therapy type and PFS curve shape (publication pending) since both manuscripts are derived from the same data set.

In Table S3, we used 1‐phase EDNLRA half‐lives for different therapy types in different malignancies to calculate the proportion of patients who would have progressed since the previous scan after different scan intervals. As illustrated in this table, a given therapy may result in longer PFS in one tumor type than another (depending on factors such as sensitivity to the therapy and on tumor cell growth rate), drug combinations often result in longer PFS than do single agents and PFS is generally longer when therapies are used as front‐line therapy compared to when they are used in previously treated patients. Hence, proportion of patients progressing per unit time would vary and optimum scan frequency should be individualized based on therapy type, tumor type, and line of therapy.

As also illustrated in Table S3, PFS half‐lives vary from one trial to another for a given therapy in a given tumor type, as would be expected. Hence, initial studies would be a reasonable basis for initial estimate of PFS, but it would be anticipated that this would be refined as additional clinical trial data, meta‐analyses and real‐world evidence are accumulated.

Table 2 shows how frequently one should do scans for a selection of illustrative therapies if one accepted a progression rate of around 20% since the prior scan as a reasonable target, recognizing that there is no consensus on the optimum targeted progression rate and that this optimum rate would vary with therapy toxicity and cost. (A higher proportion of patients progressing might be reasonable if the therapy was inexpensive and nontoxic and if there were no good alternative, while one might want to detect progression earlier with, for example, only 5%‐10% of the patients having progressed if the therapy was expensive or toxic or if there were effective alternatives. The methods we propose do not define the proportion of progressing patients who should be detected. Instead, they potentially permit one to decide the optimal scan frequency once one defines a target progression rate based on cost and other factors.)

**Table 2:** Suggested interval between scans for selected illustrative therapies if one accepted a progression rate of 20% as a reasonable target to guide interval choice

Tumor^a^	Prior Rx?	Rx^a^	Median progression‐free survival *t*_1/2_ mo	Suggested interval (wk) between scans to detect progression in ~20% of remaining patients	NCCN recommendation for interval between scans (converted to wk)
Breast	No	Single agent vinorelbine	4.8	6	6‐12
Breast	No	Other single agent chemo	5.8‐6.4	9	6‐12
Breast	No	Combination chemo	14.1	18	6‐12
Breast ER+	No	Tamoxifen	8	12	9‐24
Breast ER+	No	Fulvestrant	18.8	24	9‐24
Breast ER+	No	Aromatase inhibitors	12.9	18	9‐24
Breast HER2+	No	Trastuzumab	4.2	6	No recommendation
Breast HER2+	No	T‐DM1	14.7	18	No recommendation
Breast	Yes	Single agent chemo	3.6‐4.6	6	6‐12
Breast	Yes	Combination chemo	6.5	9	6‐12
Breast ER+	Yes	Fulvestrant	5.4	6	9‐24
Breast ER+	Yes	Aromatase inhibitors	5.8	9	9‐24
Breast HER2+	Yes	T‐DM1	7.8	12	No recommendation
Colorectal	No	Capecitabine	5.2	6	No recommendation
Colorectal	No	Combination chemo	8.3	12	No recommendation
Colorectal	Yes	Single agent TAS‐102	2.4	3	No recommendation
Colorectal	Yes	Single agent irinotecan	4.1	6	No recommendation
Colorectal	Yes	Combination chemo	4.7	6	No recommendation
Colorectal KRAS WT	Yes	EGFR monoclonals	2.5	3	No recommendation
NSCLC	No	Single agent cisplatin/pemetrexed/vinorelbine	2.6‐3.1	3	6‐12
NSCLC	No	Single agent taxanes/gemcitabine	3.6‐3.8	6	6‐12
NSCLC	No	Combination chemo	5.0	6	6‐12
NSCLC	No	PD1/PDL1 monoclonals	5.2	6	No recommendation
NSCLC	No	PD1/PDL1 monoclonals + chemo	8.6	12	No recommendation
NSCLC ALK+	No	Crizotinib	10.8	12‐18	No recommendation
NSCLC ALK+	No	Other ALK inhibitors	21.7	24	No recommendation
NSCLC EGFR‐m	No	Gefitinib/erlotinib/afatinib	10.4	12	No recommendation
NSCLC EGFR‐m	No	Osimertinib	21.1	24	No recommendation
NSCLC	Yes	Single agent chemo	3.4‐3.6	6	6‐12
NSCLC‐PDL1 unspecified	Yes	PD1/PDL1 monoclonals	4.1	6	No recommendation
NSCLC ALK+	Yes	Crizotinib	7.6	12	No recommendation
NSCLC ALK+	Yes	Other ALK inhibitors	8	12	No recommendation
NSCLC EGFR‐m	Yes	Gefitinib/erlotinib	9	12	No recommendation
Prostate	No	Antiandrogens	23.8	24‐52	No recommendation
Prostate	Yes	Abiraterone/Enzalutamide/orteronel	8.4	12	No recommendation
Renal	No	Interferon	5.5	6	6‐16
Renal	No	mTOR inhibitor	4.7	6	6‐16
Renal	No	Sorafenib	7.5	9‐12	6‐16
Renal	No	Sunitinib	10.5	12	6‐16
Renal	Yes	mTOR inhibitor	4.6	6	6‐16
Renal	Yes	Sorafenib	4.5	6	6‐16
Various	Yes or no	Placebo/BSC	3.4	3^b^	No recommendation

a Abbreviations: see Table S2.

b Suggested interval between scans for trials with placebo/BSC arms.

In 15 of 20 cases, the frequency we identified was either at or below the lower end or else at or above the upper end of the range recommended by NCCN, despite the NCCN recommended ranges being very wide, and for several examples, NCCN does not offer guidelines. The benefits of the NCCN guidelines would depend on the costs arising as a result of following them as opposed to using more or less frequent scans, and we have not specifically calculated these costs in this paper.

In Table 3, we present PFS curves that fit 2‐phase EDNLRA models, the relative size and PFS half‐lives of the two subpopulations giving rise to 2‐phase decay, and our calculation of the proportion of remaining patients who would be from the rapidly progressing subpopulation at different time points of interest after therapy initiation. This proportion would be influenced by the proportion of the original patient population that consisted of the rapidly progressing subpopulation, by the PFS half‐life of the rapidly progressing subpopulation and by the PFS half‐life of the slowly progressing subpopulation. The optimum frequency of follow‐up scans would depend on rapid and slow PFS half‐lives and on therapy vs scan costs but would decrease as the proportion of rapidly vs slowly progressing patients decreased in the remaining population since the proportion of the overall population that would be expected to progress in a given time interval would decrease.

**Table 3:** For progression‐free survival (PFS) curves fitting 2‐phase decay EDNLRA models, percentage of total population included in the rapidly progressing subpopulation, PFS half‐lives for the rapidly and slowly progressing subpopulations, and proportion of all remaining progression‐free patients who are members of the rapidly progressing subpopulation at different time points after therapy initiation

Tumor type	Therapy^a^	No. studies	% fast (median)	Fast *t*_1/2_ mo (median)	Slow *t*_1/2_ mo^b^ (median)	Adjusted slow *t*_1/2_ mo^b^ (median)	Time (mo) from initiation of therapy							
							3	6	9	12	18	24	36	48
							% of remaining patients who are from rapidly progressing subpopulation							
No prior systemic therapy														
Breast ER+	Aromatase inhibitors	10	88.9	8.2	>1000	48	86	84	80	76	67	57	36	20
Breast ER+	Fulvestrant	3	82.2	16.1	>1000	48	81	80	78	77	73	70	62	54
Breast ER+	Tamoxifen	8	83.7	4.7	91.5	48	79	71	62	53	33	18	4	1
Melanoma	ipi + nivol	7	51.2	4.1	>1000	48	40	29	21	14	6	3	0	0
Melanoma	Ipilimumab	2	90.2	3.5	>1000	48	84	76	64	51	27	12	2	0
Melanoma	PD1/PDL1	6	59.8	3.3	>1000	48	44	29	21	11	3	1	0	0
NSCLC	PD1/PDL1	4	48.5	2.9	31.2	30	34	22	13	8	3	1	0	0
NSCLC ALK+	Alectinib	2	62.6	13.9	>1000	48	59	56	52	49	44	40	33	28
NSCLC EGFR‐u	EGFR TKI	8	91.1	2.6	>1000	48	84	72	55	36	11	3	0	0
Ovary	Carboplatin	5	88.2	11.5	>1000	48	87	85	83	81	77	71	59	45
Renal	ipi + nivol	1	36.7	3.3	26.8	26.8	25	16	10	6	2	1	0	0
Renal	Interferon	8	89.5	4.3	>1000	48	82	71	61	51	32	18	4	1
Sarcoma	Doxorubicin	4	11.1	0.7	5.8	5.8	0	0	0	0	0	0	0	0
Seminoma	Carboplatin	1	29.7	7.2	>1000	48	25	21	17	14	9	6	2	1
TMB high	ipi + nivol	1	95.5	8.5	>1000	48	95	93	92	90	86	81	65	46
Prior systemic therapy														
Breast ER+	Aromatase inhibitors	10	82.3	3.5	>1000	48	74	61	52	41	22	6	1	0
Breast ER+	Fulvestrant	11	87.4	4.3	>1000	48	79	66	53	45	30	18	6	1
Colon MSI high	PD1/PDL1	1	46.7	2.2	89.5	48	26	13	6	2	0	0	0	0
Gastric	PD1/PDL1	1	89.3	1.9	>1000	48	74	50	26	11	2	0	0	0
GIST	Regorafenib	1	9.2	0.8	5.2	5.2	1	0	0	0	0	0	0	0
GIST	Sunitinib	2	94.7	6.4	>1000	48	93	93	90	86	81	72	46	21
Head/neck	PD/PDL1	2	91.8	2.3	>1000	48	82	67	46	27	6	2	0	0
Melanoma	ipi + nivol		33.4	3.2	268.5	48	21	13	8	4	1	0	0	0
Melanoma	Ipilimumab	3	92.1	3.6	>1000	48	85	76	65	52	27	11	2	0
Melanoma	PD1/PDl1	7	87.8	3.9	>1000	48	82	73	63	51	28	13	2	0
Merkel	Avelumab	1	69.2	1.6	>1000	48	39	15	5	1	0	0	0	0
NSCLC	Pemetrexed	7	95.2	3.2	>1000	48	92	87	81	75	48	21	2	0
NSCLC	PD1/PDL1	28	80.9	2.5	>1000	48	66	52	35	21	6	1	0	0
NSCLC	Pemetrexed	1	97.3	3.5	>1000	48	95	92	87	80	57	31	5	1
NSCLC ALK+	Alectinib	2	81.2	6.1	>1000	48	76	70	63	57	46	34	19	9
NSCLC EGFR‐u	EGFR TKI	33	90.8	2.4	>1000	48	79	66	49	32	10	3	0	0
Prostate	Ipilimumab	1	93.6	5.8	94	48	91	89	85	81	69	54	25	9
Renal	Nivolumab	3	86.5	3.5	>1000	48	79	70	59	46	24	10	1	0
Tumors MSI‐H	PD1/PDL1	1	29.5	2.5	52.6	48	16	8	4	2	0	0	0	0
Urothelial	PD1/PDL1	4	81.2	1.8	>1000	48	61	36	16	6	1	0	0	0
GIST	Imatinib	4	77.4	16	153	48	76	75	73	71	68	64	56	49
Maintenance therapy														
NSCLC EGFR‐u	EGFR TKI	5	92.1	2.6	>1000	48	85	72	55	36	11	3	0	0

a Abbreviations: see Table S2.

b Since the calculated slow *t*_1/2_ was generally much longer than the actual patient follow‐up, 95% confidence intervals were very large or could not be estimated. Hence, we arbitrarily adjusted the median slow *t*_1/2_ to 48 mo in situations where it was longer than that.

For these 2‐phase decay calculations, we used an adjusted PFS half‐life (48 months) for the slowly progressing group if the calculated PFS half‐life exceeded 48 months. This was done since 95% confidence intervals for long half‐lives were very wide or could not be determined if the duration of follow‐up of patients on the trial was not substantially longer than the calculated long half‐life.

In Table 4, we present PD1/PDL1 monoclonals in previously treated NSCLC as an example to illustrate how scan frequency might be adjusted with 2‐phase decay, as the proportion of the remaining population coming from the rapidly progressing group decreases. If one again accepted 20% of remaining patients progressing by the next scan, then in this example, one would do the first follow‐up scan 3‐6 weeks after therapy initiation. By 3 and 6 months after therapy initiation, follow‐up scans would be spaced at 6‐week intervals, but by 12 months, they might be spaced at 18‐week intervals, and after 24 months, could potentially be spaced at 12‐18 month intervals, with shorter term follow‐up scans if a given scan suggests the possibility of progression. This interval might also be shortened for high‐risk patients, if patients developed new symptoms or if additional follow‐up of PD1/PDL1 studies began to suggest that the PFS half‐life for the slowly progressing subpopulation is actually shorter than 48 months. In addition, with scans spaced this far apart, the scans are likely to be much less expensive than the therapy. Hence, one might consider adjusting the scan frequency to permit detection of (for example) 5% or 10% progression rate rather than a 20% progression rate.

**Table 4:** Second‐line treatment of NSCLC with PD1/PDL1 monoclonals as an illustration of how scan frequency might change over time for a therapy demonstrating 2‐phase decay

Time from Rx initiation (mo)	% of total from fast progression subpopulation^a^	% of total from slow progression subpopulation^a^	Proportion of total population progressing by next scan			
			Next scan at 3 wk^b^	Next scan at 6 wk^b^	Next scan at 12 wk^b^	Next scan at 18 wk^b^
0	81%	19%	(0.17 × 81%) + 0.01 × 19%) = 14% + 0.2% =14.2%	(0.32 × 81%) + 0.02 × 19%) = 26% + 0.4% = 26.4%	(0.54 × 81%) + (0.04 × 19%) = 44% + 0.8% = 44.8%	(0.68 × 81%) + (0.06 × 19%) = 55% + 1% = 56%
3	66%	34%	(0.17 × 66%) + (0.01 × 34%) = 11% + 0.3% = 11.3%	(0.32 × 66%) + (0.02 × 34%) = 21% + 0.7% = 21.7%	(0.54 × 66%) + (0.04 × 34%) = 36% + 1.4% = 37.4%	(0.68 × 66%) + (0.06 × 34%) = 45% + 2% = 47%
6	52%	48%	(0.17 × 52%) + (0.01 × 48%) = 9% + 0.5% = 9.5%	(0.32 × 52%) + (0.02 × 48%) = 17% + 1% = 18%	(0.54 × 52%) + (0.04 × 48%) = 28% + 2% = 30%	(0.68 × 52%) + (0.06 × 48%) = 35% + 3% = 38%
12	21%	79%	(0.17 × 21%) + (0.01 × 79%) = 4% + 0.8% = 4.8%	(0.32 × 21%) + (0.02 × 79%) = 7% + 1.6% = 8.6%	(0.54 × 21%) + (0.04 × 79%) = 11% + 3% = 14%	(0.68 × 21%) + (0.06 × 79%) = 14% + 5% = 19%

a As time from therapy initiation increases, the proportion of the remaining patients who are from the fast progression subpopulation decreases and the proportion from the slow progression subpopulation increases.

b As per Table 1, for the fast progression subpopulation (progression‐free survival (PFS) half‐life 2.5 mo), 17%, 32%, 54%, and 68% would have progressed by 3 wk, 6 wk, 12 wk, and 18 wk, respectively, while for the slow progression subpopulation (PFS half‐life 48 mo), it would be 1%, 2%, 4%, and 6%, respectively. Numbers in each cell are the proportion of the total population that would have progressed at each time point, showing the contribution to this of the fast and slow progression subpopulations.

Overall, 150 of 887 curves (17%) had moderate convexity and 42 (5%) had high convexity, with acceleration of progression rate after a downward inflection point. For high‐convexity curves, we also calculated the PFS half‐life before convexity onset and the PFS half‐life after convexity onset (Table 5) and calculated suggested scan intervals during ongoing therapy (preconvexity) vs following therapy discontinuation (postconvexity) (Table 6). For several malignancies including NSCLC,^23^ SCLC,^24^ and cancers of ovary,^25^ breast,^26^ and colon,^27^ patients who respond but have a therapy discontinued before progression may respond again with reinitiation of the same therapy once they progress. It might be advantageous to identify progression early before the deterioration of performance status.

**Table 5:** Progression‐free survival (PFS) half‐life before and after onset of convexity for PFS curves with high convexity

Author^a^	Rx^b^	Tumor type	overall PFS *t*_1/2_ mo	Time of convexity onset after Rx initiation, mo	% of population progression‐free at convexity initiation	PFS *t*_1/2_ (mo) before convexity				PFS *t*_1/2_ (mo) after convexity			
						PFS *t*_1/2_	LCI^b^	UCI^b^	*R* ^2^	PFS *t*_1/2_	LCI	UCI	*R* ^2^
Douillard	panitum + FOLFOX	colon	9.6	9.3	56	12.8	11.7	14.1	0.91	5.5	5.1	6.0	0.95
Hecht	panitum + oxal chemo + bev	colon	9.4	7.8	67	15.4	14	17.2	0.91	4.9	4.8	5.0	0.99
Schwartzberg	panitum + FOLFOX6	colon	10.4	7.0	75	21.3	17.9	26.2	0.87	6.3	5.9	6.7	0.97
Hecht	Oxal chemo + bev	colon	11.1	8.5	75	25.1	22.3	28.6	0.87	4.9	4.5	4.8	0.99
Saltz	bev+(XELOX or FOLFOX)	colon	9.6	5.8	78	21	19.6	22.6	0.97	5.5	5.2	5.9	0.96
Schwartzberg	bev + FOLFOX6	colon	9.3	7.2	75	19.7	17.8	20.4	0.97	4.8	4.6	4.9	0.98
Douillard	FOLFOX4	colon	7.9	5.3	75	15.4	13.4	17.9	0.87	5.0	4.8	5.1	0.99
Saltz	XELOX or FOLFOX	colon	7.9	4.4	77	14.5	12.9	16.6	0.93	5.0	4.8	5.2	0.99
Fuchs	FOLFIRI	colon	7.1	6.6	66	11.5	10.8	12.3	0.95	3.8	3.7	4.0	0.99
Van Cutsem	FOLFIRI	colon	7.9	7.3	62	12.6	11.8	13.6	9.92	3.7	3.6	3.8	0.99
Scagliotti	pem + cisp	NSCLC	3.7	3.8	56	4.9	4.6	5.3	0.98	2.2	2.1	2.3	0.99
Maemondo	carbo‐taxol	NSCLC	4.7	4.2	68	9.2	8.6	10	0.94	2.1	2.0	2.2	0.97
Mok	carbo‐taxol	NSCLC	5.1	5.3	64	9	8.4	9.8	0.94	2.0	1.9	2.1	0.99
Mok	carbo‐taxol	NSCLC EGFR WT	5.6	4.3	71	10.2	9	11.6	0.87	2.4	2.3	2.6	0.96
Mok	carbo‐taxol	NSCLC EGFR‐m	5.7	5.1	68	10.6	9.9	11.4	0.94	2.2	2.1	2.3	0.98
Reck	cisp‐gem	NSCLC	4.9	6.0	49	7.2	6.7	7.8	0.94	2.0	1.8	2.2	0.94
Reck	bev high dose + cisp‐gem	NSCLC	5.9	3.8	76	10.5	9.6	11.5	0.94	3.7	3.5	3.8	0.98
Reck	bev low dose + cisp‐gem	NSCLC	6.3	4.3	79	14.7	13.5	16.2	0.52	3.3	3.2	3.5	0.98
Aghajanian	bev + gem‐carbo	ovary	15.4	6.5	89	70.6	57.1	92.2	0.7	7.2	6.9	7.6	0.97
Perren	bev + carboplatin	ovary	21.2	11.7	81	52.6	46.1	61.3	0.8	10.8	10.5	11.1	0.99
Reck	platinum + etop	SCLC	4.3	4.0	75	19.4	15.1	26.8	0.58	1.1	1.0	1.3	0.94
Socinski	carbo + etop	SCLC	6.3	4.5	73	11.3	10.7	12	0.97	2.6	2.6	2.7	0.99
Tiseo	bev + cisp+etop	SCLC	6.3	4.4	75	11.8	11.1	12.7	0.97	3.4	3.3	3.6	0.98
Satouchi	amrubicin + cisp	SCLC	4.6	4.5	66	10.8	9.1	13.2	0.84	1.4	1.3	1.5	0.98
Satouchi	irinotecan + cisp	SCLC	5.5	4.6	74	15.5	12.1	21.4	0.72	1.9	1.8	2.0	0.99
von Pawel	amrubicin	SCLC	3.6	4.0	52	4.8	4.5	5.2	0.93	2.3	2.2	2.4	0.99
Reck	ipil + platinum+etop	SCLC	4.6	4.0	75	16.3	13.2	21.3	0.65	1.6	1.5	1.7	0.96
Escudier	sorafenib	renal	5.3	4.2	68	8.5	7.9	9.3	0.91	2.6	2.5	2.8	0.95
Chapman	vemurafenib	melanoma BRAF‐m	5.8	6.2	44	7.1	6.5	7.7	0.83	1.2	1.1	1.4	0.89
Amado	panitumumab	colon	2.1	1.5	87	12.9	9.5	19.7	0.63	0.3	0.3	0.4	0.86
Muro	panitumumab	colon	2.5	1.5	94	29.5	24.4	37.3	0.63	0.8	0.7	0.9	0.56
Fassnacht	lisitinib	adrenocortical	2.2	1.8	42	3	2.3	4.1	0.37	0.9	0.8	1.1	0.35
Powles	durvalumab	urothelial	1.7	1.1	89	6.5	4.9	9.8	0.63	0.4	0.4	0.6	0.71
Zimmer	ipilimumab	melanoma	3.5	1.8	93	20.8	14.8	34.8	0.8	1.7	1.5	1.9	0.85
Hodi	gp100	melanoma	2.8	2.6	72	7.6	6.4	9.2	0.79	0.51	0.43	0.6	0.81
Rao	placebo	colon	2.4	2.6	58	4.7	4.2	5.3	0.86	0.3	0.2	0.5	0.72
Pavlakis	placebo	gastric	1	0.8	77	3.6	3	4.5	0.79	0.2	0.1	0.3	0.52
Parikh	placebo	NSCLC	1.5	1.3	77	3.8	3.4	4.3	0.85	0.5	0.4	0.6	0.68
Motzer	placebo	renal	2.2	1.7	78	7.2	5.8	9.2	0.72	0.8	0.7	1.0	0.71
Jonker	BSC	colon	2.0	1.4	87	7.7	6.5	9.5	0.82	0.7	0.6	0.7	0.94
Karapetis	BSC	colon	2.1	1.2	89	9.1	8	10.6	0.9	1.0	0.9	1.0	0.95
Van Cutsem	BSC	colon	1.3	1.6	48	2.2	2	2.5	0.87	0.3	0.2	0.3	0.94

a See Table S1 for references.

b Abbreviations: see Table S2.

**Table 6:** Suggested interval between scans before onset of curve convexity (eg, prior to giving a break from therapy) and after onset of curve convexity (eg, after giving a break from therapy) for therapies associated with high convexity progression‐free survival (PFS) curves

Tumor^a^	Rx	No. studies	Median time of convexity onset, mo	Median PFS *t*_1/2_ preconvexity onset, mo	Suggested interval (wk) between scans preconvexity onset	Median PFS *t*_1/2_ postconvexity onset, mo	Suggested interval (wk) between scans postconvexity onset
Colon	Combination chemo	10	7.1	15.4	18‐24	5	6
NSCLC	Combination chemo	8	4.3	9.7	12	2.2	3
SCLC	Combination chemo	7	4.4	11.8	18^b^	1.9	3
Ovary	Combination chemo	2	9.1	61.6	40^b^	9	12
Renal	sorafenib	1	4.2^c^	8.5	12	2.6	3
Melanoma	vemurafenib	1	6.2^c^	7.1	9	1.2	3

a Other examples with high convexity had onset of the convexity at a median of 2.1 mo posttherapy initiation and were probably primarily related to the time interval between therapy initiation and early follow‐up scans.

b Around time of completion of induction chemotherapy/onset of convexity.

c Related to dose reductions or therapy discontinuation due to toxicity?

## 4 DISCUSSION

As would be expected, these analyses suggest that the shorter the PFS half‐life for a given therapy, the more frequently one should do follow‐up scans, while less frequent follow‐up scans would be reasonable for more slowly progressive tumors. These analyses provide a mechanism to calculate the optimal frequency of follow‐up scans for individual therapies.

While the analyses estimate the proportion of remaining patients who would progress in a given time interval, they do not directly indicate how frequently follow‐up scans should be done, since several different factors might be taken into consideration. We plan further pharmacoeconomic analyses to assess implications of varying scan frequency based on PFS half‐life. As discussed above, financial and toxicity costs of scans might be taken into consideration, as well as sensitivity to detect tumor size changes if scans are done too frequently. One would also consider the cost, toxicity, and inconvenience of continuing therapy that is no longer controlling a patient's tumor, the opportunity costs associated with delaying access to a potentially effective alternative, and the threshold for cost‐effectiveness of the local healthcare system.

The data presented here for different cancers and therapies estimate the rate of progression in an overall population, but they do not predict what would happen in an individual patient. They also do not predict rate of progression in a population that differs from the patients entered on the clinical trial from which the data were derived. Clinical trials eligibility criteria typically require patients with good performance status. Since poor performance status patients may progress more rapidly than good performance status patients,^28^ as may patients with high tumor bulk,^29^ patients with poor performance status or high tumor bulk would on average be expected to have more rapid tumor progression than the patients entered on the clinical trials that formed the basis for our scan frequency calculations. Hence, it would be rational to do scans more frequently than our calculations would suggest if one is dealing with a patient with poor performance status patients, high tumor bulk, an unusually aggressive cancers, or an early indication of possible tumor progression.

In addition, as would be expected, for a given therapy in a given tumor type, there was variability in PFS half‐lives across trials. In Supplementary Online Table 3, we presented proportion of patients progressing at different times based on the median PFS half‐life values across trials, and we also presented the highest and lowest PFS half‐lives calculated for that therapy/tumor pair. In some situations, the half‐life range across studies was quite narrow, while in others it was quite wide. Examples in which the median half‐life value was close to the low end of the range, with a much higher value at the high end would suggest an outlier, and further assessment might clarify driving factors.

The estimates that we provide would hopefully serve as a useful starting point, the understanding being that these estimates should be updated as additional trials are published. Meta‐analyses using patient‐specific PFS data could be particularly informative. With increasing use of electronic medical records, it will hopefully become easier to also collect the data required for eventual use of real‐world evidence to improve upon the estimates we have provided, while at the same time collecting the additional data on the link between patient characteristics and outcome that might eventually permit rational personalization of scan frequency.

PFS half‐lives correlate strongly with PFS medians (D. Stewart, unpublished data). Therefore, it would be reasonable to use medians as a surrogate for half‐lives in calculations of proportion of patients progressing per unit time. However, EDNLRA and log‐linear PFS curve plots have the advantage of easily revealing which curves follow 2‐phase decay or have convexities. These properties may not be judged reliably from Kaplan‐Meier plots. EDNLRA also permits easy estimation of the relative size and respective half‐lives for the two subpopulations leading to 2‐phase decay, the time of onset of curve convexity and the PFS half‐life before and after onset of convexity. This can help in guiding adjustment of scan frequency with increasing duration of follow‐up.

Where PFS curves fit only 1‐phase EDNLRA models, it would be reasonable to keep scan frequency the same no matter how far one is from the therapy initiation since a constant proportion of remaining patients would be expected to first show progression at each future designated point. On the other hand, where PFS curves fit 2‐phase decay models, one might consider initially having relatively frequent scans, but with progressively less frequent scans with increasing duration of follow‐up, as the proportion of patients from the rapidly progressing subpopulation decreases.

For therapies yielding PFS curve convexities due to planned therapy interruptions or due to toxicity‐related dose reductions or therapy discontinuation, our analyses support the concept of doing less frequent scans early on, during the period of full dose therapy, and more frequent scans after therapy interruption or dose reduction, particularly if potentially effective second line therapy is available. With NSCLC and SCLC (for example), it is usual to stop therapy after 4‐6 cycles, while for colon cancer, it is common to plan for continuation of therapy until progression, but for a high proportion of patients to stop before progression, due to factors such as toxicity.^30^, ^31^, ^32^, ^33^

The analyses support a number of other intuitive assumptions, including the assumption that it may be important to do scans relatively frequently in clinical trials with a placebo/BSC arm or if using agents with unproven efficacy, while less frequent scans may be reasonable when using agents known to be effective. Similarly, the analyses support doing baseline scans very shortly before initiation of therapy for rapidly progressive tumors, while longer time intervals between baseline scan and initiation of therapy would be reasonable for more slowly progressive tumors.

Ultimately, it would probably be most rational to do scans more frequently with rapidly progressive tumors and therapies that are very expensive or more toxic and to do them less frequently with tumors that are more slowly progressive, and with therapies that are relatively inexpensive and less toxic, particularly if there are no highly effective alternatives. These analyses could help in informing such decisions. Our analyses do not give a perfect final answer to the question of optimal scan frequency, but they do provide an evidence‐based starting point.

## CONFLICT OF INTEREST

DJ Stewart: honoraria for consulting/advisory boards, etc: Roche Canada, Boehringer‐Ingelheim (BI) Canada, Merck Canada, AstraZeneca (AZ) Canada, Bristol‐Myers Squib (BMS) Canada, Exactis; Clinical trials support: Celgene; DB Macdonald: no disclosures; A. Awan: honoraria for consulting/advisory boards, etc, Novartis, Apobiologix; K. Thavorn: no disclosures.
