# Supplementary material for: Optimal frequency of scans for patients on cancer therapies: A population kinetics assessment
Source: Cancer Med. 2019 Sep 27;8(16):6871–86. doi: 10.1002/cam4.2571 (PMC6853816; doi:10.1002/cam4.2571)
Supplement: Supplementary file 1 [file CAM4-8-6871-s001.pdf]

Supplementary Online Table 1. Definitions*	
Term	Definition
PFS population kinetics	Defined for this project as the rate at which progression-free patients disappear from the population
PFS first order kinetics	A given proportion of remaining patients generally progress per unit subsequent follow-up time (rather than a given number progressing, as would happen with zero order kinetics). Many biological processes (eg, drug disappearance from the blood stream) follow first order kinetics, as do many physical processes (eg, radioactive decay).
Exponential decay nonlinear regression analysis (EDNLRA)	a. 1-phase decay model: a single progression rate constant is derived for the overall population<br>b. 2-phase decay model: EDNLRA evidence of 2 distinct population subgroups with differing rates of progression. By our definitions, curves fit 2-phase decay models if by EDNLRA: i) half-lives for the 2 groups differed > 2-fold; ii) each subgroup constituted >1% of the total population
Curve Shape on log-linear plots	EDNLRA plots were converted to log-linear plots as follows: When in the graph of the EDNLRA plot in GraphPad Prism, click on "Change" in the top menu bar, then click on "Y Axis (left)" in the drop-down menu, change "Scale" from "Linear" to "Log10", click to turn off "Automatically determine the range and interval", then set the "Maximum" interval as 100. We created 6 different curve-shape classification groups (below) and designated each log-linear curve as belonging to one of these groups. The groups were:<br>a. 1-phase: straight line on log-linear plot, constant slope<br>b. "S" shaped: undulating; average slope approximates the 1-phase EDNLRA regression line. Functionally, there may be no major difference from 1-phase.<br>c. low convexity: straight line initially that approximates the 1-phase EDNLRA regression line; modest late down-turn. Functionally, there may be no major difference from 1-phase.<br>d. moderate convexity: early slight deviation above the 1-phase EDNLRA regression line; then inflection point followed by late persistent modest deviation below the 1-phase EDNLRA regression line.

	e. high convexity: early substantial deviation above the 1-phase EDNLRA regression line; then inflection point followed by late persistent marked deviation below the 1-phase EDNLRA regression line. Functionally, there may be no major difference from moderate convexity.<br>f. 2-phase: curve fits EDNLRA 2-phase decay model per our definition and/or curve initially follows or demonstrates a persistent deviation to left of the 1-phase EDNLRA regression line; then has an unequivocal inflection point followed by a persistent deviation to the right of the 1-phase EDNLRA regression line.
PFS half-life	Defined by EDNLRA decay models: time to progression or death of half the remaining patients. PFS half-life is generally very similar to and correlates strongly with PFS medians (D. Stewart, unpublished data). If one wished to do so, it would be reasonable to use PFS medians as a surrogate for PFS half-lives if the PFS curve on a log-linear plot is 1-phase, S-shaped or low convexity. Using medians would not permit adjustment for changes in optimal follow-up frequency before vs after inflection points for moderate and high convexity and 2-phase decay curves.
Calculation of proportion of remaining patients who will still be progression-free after a further defined time interval	t_n: The proposed time interval (weeks) between scans<br>*: in an Excel formula, this indicates multiplication<br>0.693: the natural logarithm of <u>2</u>, used since we are using <u>half</u>-lives<br>$t_{1/2}$: PFS half-life, in weeks<br>2^ ($-t_n / t_{1/2}$): if using the alternative approach to define proportion of patients remaining progression free, this indicates “2 to the order of ($-t_n / t_{1/2}$)”
* Note that this table is a slightly modified version of a table included in our related paper using PFS population kinetics to assess association of PFS curve shape with therapy type.	

Supplementary Online Table 2. Exponential decay nonlinear regression analysis models for different therapies and references for sources of published PFS curves

author	Tumor type	Prior Rx	no. pts	Rx	class Rx	PFS $t_{1/2}$ mo.	LCI mo	UCI mo	R^2	PFS curve shape	2-phase by NLRA	% fast	LCI %	UCI %	fast $t_{1/2}$ mo.	LCI mo	UCI mo	slow $t_{1/2}$ mo.	LCI mo	UCI mo	R2	2 phase by shape or NLRA
Ellis[1]	breast ER+	n	103	anastrazole	AI	12.6	12.1	13.2	0.96	1 phase	n											n
Howell[2]	breast ER+	y	229	anastrazole	AI	5.4	5.1	5.7	0.96	2 phase	n											y
Kaufman[3]	breast HER2+/ER+	y	104	anastrazole	AI	3.7	3.4	4.2	0.91	2 phase	y	92.3	83.3	?	3.2	2.5	4	4.90E+10	3.5	?	0.92	y
Mehta[4]	breast ER+	n	345	anastrazole	AI	13.4	13.2	13.6	0.99	1 phase	y	98.9	92.7	100	13.1	12.4	13.9	1.60E+11	7.4	infin	0.99	y
Osborne[5]	breast ER+	y	194	anastrazole	AI	5.3	4.9	5.6	0.94	2 phase	y	92.3	85.3	?	4.5	3.8	5.4	8.80E+10	?	?	0.94	y
Robertson[6]	breast ER+	n	102	anastrazole	AI	12.5	12	13	0.93	1 phase	n											n
Xu[7]	breast ER+	y	113	anastrazole	AI	5.8	5.5	6.1	0.92	2 phase	y	94.7	?	?	5.4	?	?	8.60E+11	?	?	0.92	y
Goetz[8]	breast ER+	n	165	anastrazole or letrozole	AI	14.5	14.2	14.8	0.99	1 phase	n											n
Tominaga[9]	breast ER+	y	75	fadrozole	AI	4.7	4.5	4.9	0.97	2 phase	y	88.5	10.8	97	3.8	?	?	1.30E+11	5.2	?	0.97	y
Dickler[10]	breast ER+	n	174	letrozole	AI	15	14.8	15.3	0.99	1 phase	y	98.8	94.3	100	14.7	13.8	15.7	7.70E+12	14.7	infin	0.99	y
Finn[11]	breast ER+	n	81	letrozole	AI	9	8.6	9.3	0.98	2 phase	y	28	3.1	95.3	4.1	0.9	8.2	12	9.3	?	0.98	y
Finn[12]	breast ER+	n	222	letrozole	AI	15.2	14.9	15.4	0.99	1 phase	n											n
Goss[13]	breast ER+	n	431	letrozole	AI	10.4	10.1	10.6	0.99	2 phase	n											y
Hortobagyi[14]	breast ER+	n	334	letrozole	AI	15.2	15	15.5	0.99	Con-low	n											n
Johnston[15]	breast ER+	y	390	letrozole	AI	9.2	8.8	9.5	0.96	1 phase	y	14.8	1.3	?	3.1	0.8	?	10.9	9.3	?	0.96	y
Johnston[16]	breast HER2+/ER+	n	108	letrozole	AI	4.9	4.5	5.3	0.91	2 phase	y	87	57.5	100	3.5	2.6	5.6	3.40E+15	?	?	0.95	y
Mouridsen[17]	breast ER+	n	453	letrozole	AI	10.4	10.2	10.7	0.98	2 phase	y	94.2	88.8	99.1	9.4	8.4	10.3	8.90E+10	10.7	?	0.98	y
Mouridsen[18]	breast ER+	n	458	letrozole	AI	10.8	10.4	11.2	0.97	2 phase	y	87.7	71.4	90	8.2	7	?	5.20E+15	?	?	0.99	y
Mouridsen[19]	breast ER+>70	n	152	letrozole	AI	13.3	12.9	13.6	0.99	2 phase	y	96	88.9	?	12.4	10.9	13.6	7.60E+11	?	?	0.99	y
Mouridsen[19]	breast ER+<70	n	301	letrozole	AI	9.7	9.3	10.3	0.97	2 phase	y	88.9	52.6	93.3	7.7	5.6	?	5.00E+15	?	?	0.99	y
O'Shaughnessy[20]	breast ER+	n	113	letrozole	AI	19.6	18.8	20.4	0.94	Con-low	n											n
Sonke[21]	breast ER+>65	n	145	letrozole	AI	21.5	21.1	21.9	0.99	1 phase	n											n
Sonke[21]	breast ER+<65	n	189	letrozole	AI	13.1	12.6	13.6	0.97	Con-low	n											n
Tominaga[9]	breast ER+	y	73	letrozole	AI	7.2	7	7.5	0.97	2 phase	y	40.6	18.9	78.6	2.8	1.8	4.7	14	9.7	?	0.99	y
Wolff[22]	breast ER+	n	553	letrozole	AI	8.1	2.9	8.2	0.98	1 phase	y	3.9	0.2	97.9	1.4	0.3	7.8	8.5	8	?	0.98	y
Dombernowsky[23]	breast ER+	y	188	letrozole 0.5 mg	AI	5.7	5.4	6.1	0.9	2 phase	y	77.1	64.1	81	3.4	2.9	?	4.20E+15	?	?	0.95	y
Dombernowsky[23]	breast ER+	y	174	letrozole 2.5 mg	AI	7.6	7.1	8.1	0.79	2 phase	y	72	59.6	73.2	3.5	2.9	3.9	4.30E+15	?	?	0.93	y
Gershanovich[24]	breast ER+	y	192	letrozole 0.5 mg	AI	5.8	5.5	6.2	0.94	2 phase	y	87.5	51	91.3	4.3	2.9	?	3.70E+15	?	?	0.96	y
Gershanovich[24]	breast ER+	y	185	letrozole 2.5 mg	AI	6.8	6.3	7.3	0.87	2 phase	y	60.4	40.6	?	3	2.1	4.1	21.8	12.6	?	0.95	y
Hida[25]	NSCLC ALK+	n	103	alectinib	ALK TKI	28.3	27.5	29.1	0.96	1 phase	y	75.6	58.5	?	20.2	14.4	28.9	2.00E+11	?	?	0.96	y
Ou[26]	NSCLC ALK+	y	138	alectinib	ALK TKI	8.2	8	8.5	0.97	2 phase	y	71.7	68.8	75	4.7	4.4	5.2	2.60E+10	13.4	?	0.99	y
Peters[27]	NSCLC ALK+	n	152	alectinib	ALK TKI	23.5	22.4	24.6	0.88	2 phase	y	49.6	41	51.9	7.5	6.3	8.2	3.20E+15	?	?	0.98	y
Shaw[28]	NSCLC ALK+	y	87	alectinib	ALK TKI	8.7	8.4	8.9	0.97	1 phase	y	90.6	81.9	?	7.4	6.3	8.8	4.30E+10	?	?	0.98	y
Camidge[29]	NSCLC ALK+	n	137	brigatinib	ALK TKI	19.9	19.4	20.5	0.97	2 phase	y	74.4	59.8	?	13.9	?	?	4.40E+11	?	?	0.98	y
Kim[30]	NSCLC ALK+	y	109	brigatinib arm A	ALK TKI	16.8	16.3	17.5	0.93	Con-low	n											n
Kim[30]	NSCLC ALK+	y	110	brigatinib arm B	ALK TKI	9.2	9	9.4	0.97	1 phase	n											n

Continuation of Supplementary Online Table 2. Exponential decay nonlinear regression analysis models for different therapies and references for sources of published PFS curves																						
author	Tumor type	Prior Rx	no. pts	Rx	class Rx	PFS $t_{1/2}$ mo.	LCI mo	UCI mo	R^2	PFS curve shape	2-phase by NLRA	% fast	LCI %	UCI %	fast $t_{1/2}$ mo.	LCI mo	UCI mo	slow $t_{1/2}$ mo.	LCI mo	UCI mo	R2	2 phase by shape or NLRA
Crino[31]	NSCLC ALK+	y	113	ceritinib	ALK TKI	7.4	7.2	7.6	0.98	Con-low	n											n
Kim[32]	NSCLC ALK+	n	83	ceritinib	ALK TKI	16.8	16.3	17.3	0.97	1 phase	y	93.3	67.1	?	15.3	?	?	8.70E+10	?	?	0.97	y
Kim[32]	NSCLC ALK+	y	163	ceritinib	ALK TKI	6.7	6.5	6.9	0.98	Con-low	n											n
Shaw[33]	NSCLC ALK+	y/n	114	ceritinib	ALK TKI	7.7	7.5	8	0.98	1 phase	n											n
Shaw[34]	NSCLC ALK+	y	115	ceritinib	ALK TKI	4.9	4.8	5	0.99	1 phase	n											n
Camidge[35]	NSCLC ALK+	n	149	crizotinib	ALK TKI	10.6	10.4	10.9	0.98	1 phase	n											n
Camidge[29]	NSCLC ALK+	n	138	crizotinib	ALK TKI	10.6	10.3	11	0.98	Con-low	n											n
Hida[25]	NSCLC ALK+	n	104	crizotinib	ALK TKI	24.9	18.5	37.4	0.98	1 phase	n											n
Nishio[36]	NSCLC ALK+	n	172	crizotinib	ALK TKI	10.9	10.6	11.1	0.99	2 phase	y	95.1	90.6	?	10	9.2	10.9	6.30E+11	19.9	?	0.99	y
Nishio[36]	NSCLC ALK+	y	173	crizotinib	ALK TKI	7.6	7.3	7.9	0.97	1 phase	n											n
Peters[27]	NSCLC ALK+	n	151	crizotinib	ALK TKI	11.6	11.3	12	0.97	Con-low	n											n
Shaw[37]	NSCLC ALK+	y	173	crizotinib	ALK TKI	7.6	7.3	8	0.95	1 phase	n											n
Shaw[38]	NSCLC ROS1+	n	50	crizotinib	ALK TKI	20	19.5	20.5	0.97	1 phase	y	8.6	1.1	89.9	4.6	1.1	?	23.8	20.5	?	0.97	y
Solomon[39]	NSCLC ALK+	n	172	crizotinib	ALK TKI	10.6	10.4	10.8	0.99	S	n											n
Cainap[40]	hepatocell	n	514	linafanib	angio inh	4.8	4.6	4.9	0.99	1 phase	n											n
Kang[41]	renal	y	134	axitinib	angio inh	3.7	3.5	3.9	0.96	1 phase	n											n
Li[42]	gastric	y	176	apatinib	angio inh	3	2.7	3.2	0.91	S	n											n
Llovet[43]	hepatocell	y	263	brivanib	angio inh	4	3.7	4.3	0.94	Con-low	n											n
Motzer[44]	renal	y/n	260	tivozanib	angio inh	12.4	12.1	12.7	0.98	1 phase	n											n
Motzer[45]	renal	y	52	lenvatinib	angio inh	8	7.7	8.4	0.96	1 phase	n											n
Schlumberger[46]	thyroid	y	261	lenvatinib	angio inh	17.6	17.4	17.7	0.99	1 phase	y	1.8	0.9	3.9	1.3	0.4	3.7	18.2	17.8	18.8	0.99	y
Sternberg[47]	renal	y/n	290	pazopanib	angio inh	10.1	9.8	10.4	0.98	1 phase	y	88.2	?	?	8.3	?	?	612.7	?	?	0.98	y
van der Graaf[48]	sarcoma	y	246	pazopanib	angio inh	4.2	4.1	4.4	0.97	Con-low	n											n
von Pawel[49]	SCLC	y	424	amrubicin	anthra-other	3.6	3.4	3.8	0.96	Con-high	n											n
Crump[50]‡	breast	n	112	anthra or taxane	anthra-other	9.7	9.2	10.1	0.97	2 phase	y	92.7	47.9	100	8.3	5.7	14.9	3.20E+15	?	?	0.97	y
Chan[51]	breast	y	165	doxorubicin	anthra-other	5	4.8	5.2	0.97	Con-low	n											n
Gish[52]	hepatocell	n	223	doxorubicin	anthra-other	4.1	3.7	4.5	0.89	S	n											n
Henderson[53]	breast	y	146	doxorubicin	anthra-other	3.5	3.2	3.7	0.93	Con-mod	n											n
Ingle[54]	breast	y	74	doxorubicin	anthra-other	3.7	3.6	3.8	0.98	2 phase	y	98.4	95.2	?	3.6	3.4	3.8	2.90E+11	?	?	0.98	y
Kramer[55]	breast	n	146	doxorubicin	anthra-other	5.1	4.5	5.7	0.74	Con-mod	n											n
Paridaens[56]	breast	n	165	doxorubicin	anthra-other	6.4	6	6.8	0.94	Con-mod	n											n
Qin[57]	hepatocell	n	187	doxorubicin	anthra-other	2.6	2.3	3	0.83	S	n											n
Reyno[58]	breast	y/n	152	doxorubicin	anthra-other	5.3	5.1	5.4	0.98	S	n											n
Sledge[59]	breast	n	224	doxorubicin	anthra-other	5.6	5.4	5.7	0.98	Con-low	n											n
Thigpen[60]	endometrial	n	132	doxorubicin	anthra-other	3.4	3.4	3.5	0.96	Con-low	n											n
Thigpen[61]	endometrial	n	150	doxorubicin	anthra-other	4.2	4.1	4.3	0.99	2 phase	y	96.8	?	99.8	3.9	1.9	?	1.40E+16	?	?	0.99	y
Henderson[53]	breast	y	156	mitoxantrone	anthra-other	2.7	2.6	2.9	0.98	1 phase	n											n
Pujade-Lauraine[62]‡	ovary	y	182	PEG doxo or paclitax or topot	anthra-other	3.4	3.1	3.7	0.93	S	n											n
Judson[63]	sarcoma	n	228	doxorubicin	anthra-sarcoma	4.8	4.6	5.1	0.97	2 phase	y	93.1	91.6	94.6	4	3.8	4.2	2.70E+10	15.8	?	0.99	y
Lorigan[64]	sarcoma	n	110	doxorubicin	anthra-sarcoma	3	2.8	3.2	0.94	2 phase	n											y

Continuation of Supplementary Online Table 2. Exponential decay nonlinear regression analysis models for different therapies and references for sources of published PFS curves

author	Tumor type	Prior Rx	no. pts	Rx	class Rx	PFS $t_{1/2}$ mo.	LCI mo	UCI mo	R^2	PFS curve shape	2-phase by NLRA	% fast	LCI %	UCI %	fast $t_{1/2}$ mo.	LCI mo	UCI mo	slow $t_{1/2}$ mo.	LCI mo	UCI mo	R2	2 phase by shape or NLRA
Martin-Broto[65]	sarcoma	n	59	doxorubicin	anthra-sarcoma	4.5	4.2	4.9	0.92	2 phase	y	19.8	16.4	23.6	0.3	0.2	0.5	6.2	5.8	6.7	0.99	y
Maurel[66]	sarcoma	n	67	doxorubicin	anthra-sarcoma	5.2	5	5.4	0.99	1 phase	y	2.4	?	?	0.6	?	?	5.4	5.1	?	0.99	y
Tap[67]	sarcoma	n	323	doxorubicin	anthra-sarcoma	5.1	5	5.3	0.99	2 phase	y	2.1	?	?	0.7	?	?	5.3	?	?	0.99	y
Ryan[68]	prostate	y	546	abirat + pred	antiandrogen	16.6	16.2	17	0.98	1 phase	n											n
de Bono[69]	prostate	y	797	abiraterone	antiandrogen	6	5.8	6.3	0.95	Con-low	n											n
Sweeney[70]	Prostate	n	393	antiandrogen	antiandrogen	23.8	22.9	24.8	0.97	1 phase	y	82.7	76.4	89	17	?	?	4.60E+12	185	infin	0.99	y
Beer[71]	prostate	y	872	enzalutamide	antiandrogen	22.8	21.8	23.8	0.94	1 phase	n											n
Scher[72]	prostate	y	800	enzalutamide	antiandrogen	8.4	8	8.7	0.96	Con-low	n											n
Fizazi[73]	prostate	y	732	orteronel + prednisone	antiandrogen	8	7.8	8.3	0.97	S	n											n
Ardizzoni[74]	NSCLC	y	120	pemetrexed	Antifolate	3.4	3.3	3.5	0.99	2 phase	y	97.2	?	?	3.2	?	3.4	4.70E+15	?	?	0.99	y
Boeck[75]	pancreas	y	52	pemetrexed	Antifolate	2.1	2	2.2	0.94	2 phase	n											y
Bylicki	NSCLC	y	130	pemetrexed	Antifolate	4	3.8	4.2	0.97	2 phase	n											y
Ciuleanu[76]	NSCLC	Mnt	441	pemetrexed	Antifolate	4.5	4.3	4.6	0.99	1 phase	n											n
Cullen[77]	NSCLC	y	290	pemet-low dose	Antifolate	3	2.8	3.1	0.97	2 phase	n											y
Cullen[77]	NSCLC	y	291	pemet-high dose	Antifolate	3	2.9	3.2	0.97	2 phase	n											y
de Boer[78]	NSCLC	y	278	pemetrexed	Antifolate	3.2	3	3.4	0.96	Con-low	n											n
Dittrich[79]	NSCLC	y	83	pemetrexed	Antifolate	3	2.8	3.2	0.95	Con-low	n											n
Gautschi[80]	NSCLC	Mnt	52	pemetrexed	Antifolate	4.8	4.5	5.2	0.94	S	y	7.7	?	?	0.1	?	?	5.5	?	?	0.96	y
Gish[52]	hepatocell	n	222	nolatrexed	Antifolate	3.9	3.6	4.2	0.91	2 phase	n											y
Hanna[81]	NSCLC	y	283	pemetrexed	Antifolate	3	2.9	3	0.99	2 phase	n											y
Hanna[82]	NSCLC	y	360	pemetrexed	Antifolate	3.9	3.8	4.1	0.99	2 phase	y	92.1	82.6	93.9	3.2	2.9	?	4.40E+15	?	?	0.99	y
Jassem[83]	mesothelioma	y	123	pemetrexed	Antifolate	3.4	3.3	3.6	0.98	2 phase	n											y
Karampeazis[84]	NSCLC	y	166	pemetrexed	Antifolate	3.3	3.3	3.4	0.99	2 PHASE	y	97.8	infin	?	3.2	?	3.4	4.20E+15	?	?	0.98	y
Karayama[85]	NSCLC	Mnt	55	pemetrexed	Antifolate	8.7	8.2	9.2	0.96	Con-low	n											n
Kim[86]	NSCLC	y	304	pemetrexed	Antifolate	3.4	3.1	3.6	0.96	2 phase	n											y
Lee[87]	NSCLC nonsmoker	y	80	pemetrexed	Antifolate	4.6	4.6	4.7	0.96	2 phase	y	98.7	97.3	?	4.5	4.3	?	5.00E+11	?	?	0.99	y
Li[88]	NSCLC	y	62	pemetrexed	Antifolate	4.6	4.3	4.9	0.96	2 phase	n											y
Paz-Ares[89]	NSCLC nonsqu	Mnt	359	pemetrexed	Antifolate	4.9	4.8	5.1	0.99	2 phase	y	93.6	69.7	100	4.4	3.6	5.6	29	4.2	infin	0.99	y
Shaw[37]‡	NSCLC ALK+	y	174	pemet or docet	Antifolate	3.7	3.6	3.8	0.97	2 phase	y	97.3	92.6	?	3.5	3.1	3.8	1.40E+11	3.6	?	0.97	y
Smit[90]	NSCLC	y	121	pemetrexed	Antifolate	2.6	2.5	2.8	0.91	Con-low	n											n
Sun[91]	NSCLC	y	67	pemetrexed	Antifolate	3.6	3.5	3.7	0.98	2 phase	y	95.2	91.3	97.1	3.2	3.1	3.4	6.20E+15	?	?	0.98	y

Continuation of Supplementary Online Table 2. Exponential decay nonlinear regression analysis models for different therapies and references for sources of published PFS curves

author	Tumor type	Prior Rx	no. pts	Rx	class Rx	PFS $t_{1/2}$ mo.	LCI mo	UCI mo	R^2	PFS curve shape	2-phase by NLRA	% fast	LCI %	UCI %	fast $t_{1/2}$ mo.	LCI mo	UCI mo	slow $t_{1/2}$ mo.	LCI mo	UCI mo	R2	2 phase by shape or NLRA
Zhou[92]	NSCLC EGFR WT	y	76	pemetrexed	Antifolate	5.8	5.6	5.9	0.99	Con-low	n											n
Zukin[93]	NSCLC	n	102	pemetrexed	Antifolate	2.7	2.6	2.8	0.99	Con-low	n											n
Long[94]	melanoma B-m	n	212	dabrafenib	BRAF TKI-m	8.8	8.4	9.2	0.95	1 phase	n											n
Chapman[95]	melanoma	n	337	vemurafenib	BRAF TKI-m	5.8	5.3	6.2	0.84	Con-high	n											n
Larkin[96]	melanoma B-m	n	248	vemurafenib	BRAF TKI-m	6.7	6.4	7.1	0.91	S	n											n
McArthur[97]†	melanoma B-m	y	56	vemurafenib	BRAF TKI-m	4.5	4.2	4.8	0.93	Con-mod	n											n
McArthur[97]†	melanoma B-m	n	90	vemurafenib	BRAF TKI-m	3.5	3.2	3.7	0.92	Con-mod	n											n
Robert[98]	melanoma B-m	n	352	vemurafenib	BRAF TKI-m	7.3	7.1	7.5	0.95	1 phase	n											n
Sosman[99]	melanoma B-m	y	132	vemurafenib	BRAF TKI-m	6.6	6.4	6.9	0.98	1 phase	n											n
Dickler[10]	breast ER+	n	174	letroz+bev	combo AI	19.9	19.4	20.5	0.98	1 phase	n											n
Johnston[16]	breast HER2+	n	111	lapat+letroz	combo AI	7.7	7.3	8	0.97	S	n											n
Kaufman[3]	breast HER2+/ER+	y	103	trastuz+letroz	combo AI	6.1	5.5	6.7	0.93	2 phase	y	87.4	73.6	89.8	4.3	3.8	?	4.70E+15	?	?	0.98	y
Mehta[4]	breast ER+	n	349	anastr+fulv	combo AI	16.9	16.5	17.3	0.99	2 phase	y	59.3	44	74.6	9.6	8.3	11.5	36	27.3	53.2	0.99	y
Johnson[100]	NSCLC EGFR-u	Mnt	370	erlot+bev	combo EGFR-u	5.1	4.9	5.2	0.98	S	n											n
Scagliotti[101]	NSCLC	y	480	sunit+erlot	combo EGFR-u	3.8	3.7	4	0.97	2 phase	y	97.4	78.6	100	3.6	3	4.6	3.40E+15	?	?	0.97	y
Scagliotti[102]	NSCLC nonsqu	y	526	erlot+tevant	combo EGFR-u	3.5	3.3	3.6	0.97	1 phase	y	96.7	45	100	3.2	2.2	6.2	4.20E+15	?	?	0.97	y
Andersson[103]	breast HER2+	n	141	vinor+trastuz	combo HER2	16.2	15.6	16.8	0.97	S	n											n
Andersson[103]	breast HER2+	n	143	docet+trastuz	combo HER2	15.3	14.7	15.9	0.96	2 phase	y	89	84.3	94.9	12.3	?	?	3.50E+11	28.2	?	0.97	y
Baselga[104]	breast HER2+	n	402	pertuz+trastuz+ docet	combo HER2	19.3	.18.8	19.8	0.94	1 phase	n											n
Baselga[104]	breast HER2+	n	406	trastuz+docet	combo HER2	12.1	11.8	12.4	0.98	1 phase	n											n
Baselga[105]	breast HER2+	n	181	paclit+trastuz+ lip doxo	combo HER2	16.1	15.5	16.6	0.96	Con-low	n											n
Baselga[105]	breast HER2+	n	182	paclit+trastuz	combo HER2	14.1	13.6	14.6	0.97	Con-low	n											n
Blackwell[106]	breast HER2+	y	148	trastuz+lapat	combo HER2	2.9	2.7	3	0.97	S	n											n
Chan[107]	breast HER2+	n	69	vinor+trastuz	combo HER2	9	8.8	9.2	0.99	S	y	98.5	0.6	?	8.8	8.1	9.2	9.90E+12	8.8	?	0.99	y
Chan[108]	breast HER2+	n	50	vinor+cape+ trastuz	combo HER2	13.3	12.4	14.2	0.89	Con-mod	n											n
Dang[109]	breast HER2+	y/n	67	paclit+trastuz+ pertuz	combo HER2	20.4	19.7	21.2	0.95	Con-low	n											n
Gelmon[110]	breast HER2+	n	267	trastuz+paclit	combo HER2	11.7	11.1	12.4	0.94	Con-mod	n											n
Hurvitz[111]	breast HER2+	n	239	paclit+ trastuz	combo HER2	18.6	18.2	19	0.98	S	n											n
Hurvitz[111]	breast HER2+	n	480	paclit+trastuz+ evero	combo HER2	21.5	21.1	21.9	0.99	2 phase	n											y
Martin[112]	breast HER2+	n	88	cape+bev+trastuz	combo HER2	16.2	15.6	16.9	0.95	1 phase	n											n

Continuation of Supplementary Online Table 2. Exponential decay nonlinear regression analysis models for different therapies and references for sources of published PFS curves

author	Tumor type	Prior Rx	no. pts	Rx	class Rx	PFS $t_{1/2}$ mo.	LCI mo	UCI mo	R^2	PFS curve shape	2-phase by NLRA	% fast	LCI %	UCI %	fast $t_{1/2}$ mo.	LCI mo	UCI mo	slow $t_{1/2}$ mo.	LCI mo	UCI mo	R2	2 phase by shape or NLRA
Perez[113]	breast HER2+	n	106	vinor+trastuz+ pertuz	combo HER2	14.4	13.9	14.9	0.97	1 phase	n											n
Pivot[114]	breast HER2+	y	269	trastuz+cape	combo HER2	9	8.5	9.6	0.93	S	n											n
Robert[115]	breast HER2+	n	98	paclit+trastuz	combo HER2	7.9	7.6	8.1	0.98	Con-low	n											n
Robert[115]	breast HER2+	n	98	paclit+trastuz+ carbo	combo HER2	14.2	13.4	15	0.94	Con-mod	n											n
Swain[116]	breast HER2+	n	335	docet+trastuz+ pertuz	combo HER2	21.8	21.3	22.4	0.98	2 phase	y	91.1	81.2	100	18.4	16.6	20.7	1.80E+12	49.8	infin	0.98	y
Swain[116]	breast HER2+	n	369	docet+trastuz	combo HER2	14.3	13.8	14.9	0.96	2 phase	y	91.1	61.3	100	11.7	8.9	17	4.70E+15	?	?	0.97	y
Toi[117]	breast HER2+/As	n	105	paclit+trastuz	combo HER2	15.9	15.1	16.7	0.93	S	n											n
Toi[117]	breast HER2+/As	n	198	paclit+trastuz+ everlo	combo HER2	20	19.4	20.7	0.97	1 phase	n											n
Valerio[118]	breast HER2+	n	132	docet+carbo+ trastuz	combo HER2	11.1	10.2	12	0.92	Con-mod	n											n
Valero[118]	breast HER2+	n	131	docet+trastuz	combo HER2	12.1	11.6	12.7	0.97	1 phase	n											n
Wardley[119]	breast HER2+	n	110	docet+trastuz	combo HER2	13.1	12.8	13.4	0.99	1 phase	n											n
Wardley[119]	breast HER2+	n	112	docet+trastuz+ cape	combo HER2	17.9	17.4	18.8	0.97	1 phase	n											n
Govindan[120]	NSCLC squam	n	388	ipi+chemo	Combo Ipi/other	6.2	5.9	6.5	0.94	2 phase	n											y
Hodi[121]	melanoma	y	403	ipi+gp100	Combo Ipi/other	2.6	2.4	2.9	0.91	2 phase	n											y
Lynch[122]	NSCLC	n	68	ipi phased+chemo	Combo Ipi/other	6.6	5.9	7.3	0.89	Con-mod	n											n
Lynch[122]	NSCLC	n	70	ipi concurrent +chemo	Combo Ipi/other	5.4	5.1	5.7	0.96	Con-mod	n											n
Reck[123]	SCLC	n	478	ipi+plat+etop	Combo Ipi/other	4.6	4.1	5.1	0.83	Con-high	n											n
Robert[124]	melanoma	n	250	ipi+dacarb	Combo Ipi/other	4	3.7	4.2	0.89	2 phase	y	86.8	71.5	100	2.8	2.3	3.6	5.30E+15	?	?	0.94	y
Fassnacht[125]	adrenal	n	151	mitotane+cisp+ doxo+etop	combo- other	6.1	5.7	6.4	0.96	2 phase	y	75.4	49.6	90.1	3.9	2.9	?	23.4	11.4	?	0.98	y
Fassnacht[125]	adrenal	n	153	mitotane+streptoz	combo- other	2.7	2.5	3	0.87	2 phase	n											y
Valle[126]	biliary	y	204	cisp+gem	combo- other	6.6	6.3	7	0.95	Con-mod	n											n
Albain[127]	breast	y	266	gem+paclit	combo- other	6.5	6.1	6.9	0.97	2 phase	n											y
Baselga[128]	breast	y	114	soraf+cape	combo- other	7	6.7	7.4	0.94	Con-low	n											n
Crump[50]	breast	n	112	high dose carbo+CTX+mitox	combo- other	15.4	14.9	15.4	0.98	2 phase	y	86.6	66.8	100	11.2	9.2	14.3	5.20E+15	?	?	0.99	y
Miller[129]	breast	n	347	bev+paclit	combo- other	10.6	10.2	11.1	0.98	Con-mod	n											n
Park[130]	breast	Mnt	116	paclit+gem	combo- other	7.3	6.9	7.7	0.96	Con-mod	n											n
Rugo[131]	breast	n	245	ixabepilone+bev	combo- other	7.5	7.2	7.8	0.97	1 phase	n											n
Rugo[131]	breast	n	283	paclit+bev	combo- other	11.5	10.9	12	0.95	S	n											n
Sparano[132]	breast	y	378	PEG doxo+docet	combo- other	8.4	8	8.9	0.97	Con-low	n											n

Continuation of Supplementary Online Table 2. Exponential decay nonlinear regression analysis models for different therapies and references for sources of published PFS curves

author	Tumor type	Prior Rx	no. pts	Rx	class Rx	PFS $t_{1/2}$ mo.	LCI mo	UCI mo	R^2	PFS curve shape	2-phase by NLRA	% fast	LCI %	UCI %	fast $t_{1/2}$ mo.	LCI mo	UCI mo	slow $t_{1/2}$ mo.	LCI mo	UCI mo	R2	2 phase by shape or NLRA
Sparano[133]	breast	y	609	ixabepilone+cape	combo- other	5.8	5.5	6.1	0.97	Con-low	n											n
Stockler[134]	breast	n	109	CMF	combo- other	5.9	5.7	6.2	0.98	Con-mod	n											n
Baselga[135]	breast ER+	y	485	exem+evero	combo- other	11.2	10.9	11.6	0.97	1 phase	n											n
Gelmon[110]	breast HER2+	n	270	lapat+paclit	combo- other	9.1	8.4	10	0.89	Con-mod	n											n
Guan[136]	breast HER2+	n	222	lapat+paclit	combo- other	9.7	9	10.4	0.92	Con-low	n											n
Pivot[114]	breast HER2+	y	271	lapat+cape	combo- other	6.9	6.5	7.3	0.95	S	n											n
Verma[137]	breast HER2+	y	496	lapat+cape	combo- other	6.4	6.1	6.7	0.96	Con-low	n											n
O'Shaughnessy[138]	breast triple-	y	258	gem+carbo	combo- other	3.8	3.7	3.9	0.99	1 phase	n											n
O'Shaughnessy[138]	breast triple-	y	261	iniparib+gem+ carbo	combo- other	4.9	4.7	5.1	0.98	Con-low	n											n
Monk[139]	cervix	n	103	cisp+paclit	combo- other	6.1	5.9	6.2	0.98	Con-low	n											n
Monk[139]	cervix	n	112	gem+cisp	combo- other	4.3	4.2	4.5	0.97	Con-mod	n											n
Tewari[140]	cervix	n	112	topot+bev	combo- other	5.4	5.3	5.5	0.99	Con-low	n											n
Tewari[140]	cervix	n	115	cisp+bev	combo- other	7.4	7.2	7.7	0.98	Con-low	n											n
Douillard[141]	colon	n	590	FOLFOX	combo- other	7.9	7.4	8.6	0.92	Con-high	n											n
Douillard[141]	colon	n	593	panit+FOLFOX	combo- other	9.6	8.9	10.4	0.92	Con-high	n											n
Falcone[142]	colon	n	113	FOLFIRI	combo- other	5.6	5.4	5.9	0.97	Con-mod	n											n
Falcone[142]	colon	n	122	FOLFOXIRI	combo- other	8.3	7.6	9.1	0.87	Con-mod	n											n
Fuchs[143]	colon	n	141	bolus FU FOLFIRI	combo- other	5.4	5	5.9	0.93	Con-mod	n											n
Fuchs[143]	colon	n	144	FOLFIRI	combo- other	7.1	6.6	7.6	0.94	Con-high	n											n
Fuchs[143]	colon	n	145	cape+irino	combo- other	5.4	5.1	5.7	0.96	Con-low	n											n
Giantonio[144]	colon	y	286	FOLFOX+bev	combo- other	6.1	5.7	6.6	0.94	Con-mod	n											n
Giantonio[144]	colon	y	291	FOLFOX	combo- other	4.4	4.1	4.7	0.94	Con-mod	n											n
Haller[145]	colon	y	318	oxal+irino	combo- other	4.7	4.6	4.9	0.98	Con-low	n											n
Hecht[146]	colon	n	410	oxal combo+bev	combo- other	11.1	9.9	12.6	0.83	Con-high	n											n
Hecht[146]	colon	n	413	panit+oxal combo+bev	combo- other	9.4	8.6	10.3	0.90	Con-high	n											n
Hoff[147]	colon	n	358	FOLFOX/CAPEOX	combo- other	6.6	6.2	7	0.94	Con-mod	n											n
Hoff[147]	colon	n	502	cedir+oxal+FU or cape	combo- other	7.7	7.2	8.2	0.92	Con-mod	n											n
Kim[148]	colon	y	246	FOLFOX	combo- other	5.6	5.3	5.8	0.98	Con-low	n											n
Loupakis[149]	colon	n	252	FOLFOXIRI+bev	combo- other	11.6	11.1	12	0.96	S	n											n
Loupakis[149]	colon	n	256	FOLFIRI+bev	combo- other	9.2	8.7	9.6	0.95	S	n											n
Saltz[150]	colon	n	699	XELOX or FOLFOX	combo- other	7.9	7.2	8.7	0.92	Con-high	n											n
Saltz[150]	colon	n	701	bev+XELOX or FOLFOX	combo- other	9.6	8.6	10.8	0.87	Con-high	n											n
Schwartzberg[151]	colon	n	139	panit+FOLFOX	combo- other	10.4	9.4	11.6	0.91	Con-high	n											n
Schwartzberg[151]	colon	n	139	bev+FOLFOX	combo- other	9.3	8.5	10.2	0.89	Con-high	n											n
Siu[152]	colon	y	376	brivanib+cetux	combo- other	4.1	3.9	4.4	0.96	Con-mod	n											n

Continuation of Supplementary Online Table 2. Exponential decay nonlinear regression analysis models for different therapies and references for sources of published PFS curves

author	Tumor type	Prior Rx	no. pts	Rx	class Rx	PFS $t_{1/2}$ mo.	LCI mo	UCI mo	R^2	PFS curve shape	2-phase by NLRA	% fast	LCI %	UCI %	fast $t_{1/2}$ mo.	LCI mo	UCI mo	slow $t_{1/2}$ mo.	LCI mo	UCI mo	R2	2 phase by shape or NLRA
Sobrero[153]	colon	y	648	cetux+irino	combo- other	3.5	3.3	3.6	0.98	Con-mod	n											n
Tebbutt[154]	colon	n	157	bev+cape	combo- other	7.5	7.1	7.9	0.92	Con-mod	n											n
Tebbutt[154]	colon	n	158	bev+mito+cape	combo- other	7.6	7.1	8.1	0.93	Con-mod	n											n
Tol[155]	colon	n	377	cetux+bev+cape+ oxal	combo- other	8.8	8.4	9.2	0.95	S	n											n
Tol[155]	colon	n	378	bev+cape+oxal	combo- other	10.3	9.8	10.9	0.94	Con-mod	n											n
Van Cutsem[156]	colon	n	599	FOLFIRI	combo- other	7.9	7.3	8.5	0.88	Con-high	n											n
Van Cutsem[156]	colon	n	599	cetux+FOLFIRI	combo- other	9	8.6	9.5	0.94	Con-mod	n											n
Van Cutsem[157]	colon	y	426	PTK/ZK+FOLFOX	combo- other	4.7	4.3	5.1	0.93	Con-mod	n											n
Van Cutsem[157]	colon	y	429	FOLFOX	combo- other	3.9	3.6	4.3	0.92	Con-mod	n											n
Van Cutsem[158]	colon	y	612	aflib+FOLFIRI	combo- other	6	5.6	6.4	0.93	Con-mod	n											n
Van Cutsem[158]	colon	y	614	FOLFIRI	combo- other	4.6	4.5	4.8	0.99	1 phase	n											n
Peeters[159]	colon KRAS WT	y	294	FOLFIRI	combo- other	4.4	4.1	4.6	0.96	Con-low	n											n
Peeters[159]	colon KRAS WT	y	303	panit+FOLFIRI	combo- other	5.6	5.2	6	0.94	Con-mod	n											n
Ohtsu[160]	gastric	n	387	cape+cisp	combo- other	4.9	4.7	5.1	0.97	S	n											n
Ohtsu[160]	gastric	n	387	bev+cape+cisp	combo- other	6	5.8	6.3	0.96	Con-mod	n											n
Hecht[161]	gastric HER2+	n	238	cape+oxal	combo- other	4.9	4.6	5.3	0.93	S	n											n
Hecht[161]	gastric HER2+	n	249	lapat+cape+oxal	combo- other	6.2	5.9	6.5	0.97	Con-low	n											n
Brada[162]	glioma	n	224	PCV	combo- other	3.7	3.6	3.8	0.99	S	n											n
Chinot[163]	glioma	n	458	bev+XRT+temoz	combo- other	10.2	9.5	10.9	0.92	Con-mod	n											n
Chinot[163]	glioma	n	463	XRT+temoz	combo- other	6.8	6.5	7.1	0.98	1 phase	n											n
Gilbert[164]	glioma	n	317	XRT+temoz	combo- other	7.6	7.4	7.8	0.99	2 phase	y	92.4	85.7	?	6.3	5.9	?	3.40E+15	?	?	0.99	y
Gilbert[164]	glioma	n	320	bev+XRT+temoz	combo- other	10.2	9.6	10.7	0.93	Con-mod	n											n
Vermorken[165]	head/neck	n	220	FU+plat	combo- other	3.2	3	3.4	0.94	Con-mod	n											n
Vermorken[165]	head/neck	n	222	cetux+FU+plat	combo- other	4.8	4.5	5.2	0.93	Con-mod	n											n
Qin[57]	hepatocell	n	184	FOLFOX	combo- other	3.6	3.3	3.9	0.91	S	n											n
Atkins[166]	melanoma	n	195	cisp+vinbl+dacarb	combo- other	2.8	2.6	2.9	0.97	2 phase	y	86.2	81.3	91.2	2.2	2	2.4	31.3	20	72.1	0.99	y
Atkins[166]	melanoma	n	200	IL2+IFN+chemo	combo- other	4.6	4.4	4.8	0.98	2 phase	y	87.4	81.8	93.1	3.6	3.4	4	35.2	21.1	106.9	0.99	y
Larkin[96]	melanoma B-m	n	247	cobem+vemur	combo- other	11.9	11.4	12.4	0.94	1 phase	n											n
Long[94]	melanoma B-m	n	211	dabra+tramet	combo- other	10.9	10.2	11.7	0.88	Con-low	n											n
Robert[98]	melanoma B-m	n	352	dabra+tramet	combo- other	12.5	12.3	12.8	0.96	1 phase	n											n
Abe[167]	NSCLC	n	138	docet+cisp	combo- other	4.6	4.3	4.8	0.95	2 phase	n											y
Belani[168]	NSCLC	n	55	pemet+cisp+axit contin	combo- other	8.9	8.1	9.9	0.84	Con-mod	n											n
Belani[168]	NSCLC	n	55	pemet+carbo	combo- other	7.4	6.9	8	0.93	Con-mod	n											n
Belani[168]	NSCLC	n	58	pemet+cisp+axit modif	combo- other	8.3	7.6	9.1	0.86	Con-mod	n											n

Continuation of Supplementary Online Table 2. Exponential decay nonlinear regression analysis models for different therapies and references for sources of published PFS curves

author	Tumor type	Prior Rx	no. pts	Rx	class Rx	PFS $t_{1/2}$ mo.	LCI mo	UCI mo	R^2	PFS curve shape	2-phase by NLRA	% fast	LCI %	UCI %	fast $t_{1/2}$ mo.	LCI mo	UCI mo	slow $t_{1/2}$ mo.	LCI mo	UCI mo	R2	2 phase by shape or NLRA
Crawford[169]	NSCLC	n	54	carbo+paclit	combo- other	4.4	4.1	4.7	0.94	Con-mod	n											n
Crawford[169]	NSCLC	n	112	panit+carbo+ paclit	combo- other	3.7	3.6	3.9	0.98	Con-low	n											n
Han[170]	NSCLC	n	150	gem+cisp	combo- other	5.4	4.9	6	0.83	Con-mod	n											n
Heigener[171]	NSCLC	n	140	carbo+vinor	combo- other	4.7	4.4	5	0.92	Con-mod	n											n
Janne[172]	NSCLC	n	63	gem+cisp	combo- other	6.3	6	6.6	0.97	S	n											n
Janne[172]	NSCLC	n	66	LY 600 mg+gem+ cisp	combo- other	5.4	5	5.9	0.93	Con-low	n											n
Janne[172]	NSCLC	n	71	LY 200 mg+gem+ cisp	combo- other	3.9	3.7	4.2	0.95	Con-low	n											n
Kubota[173]	NSCLC	n	303	docet+cisp	combo- other	4.6	4.4	4.8	0.98	2 phase	n											y
Kubota[173]	NSCLC	n	305	S-1+cisp	combo- other	4.4	4.2	4.6	0.96	Con-low	n											n
Langer[174]	NSCLC	n	332	carbo+paclit	combo- other	4.2	3.9	4.5	0.94	Con-mod	n											n
Langer[174]	NSCLC	n	338	carbo+paclit+ figit	combo- other	4.2	3.8	4.7	0.9	Con-mod	n											n
Lilenbaum[175]	NSCLC	n	51	carbo+paclit	combo- other	3	2.9	3.1	0.97	S	n											n
Ma[176]	NSCLC	n	264	plat+docet	combo- other	6.1	5.5	6.6	0.94	1 phase	y	31.2	26.2	36.1	1.2	1	1.6	10.3	9.6	11.2	0.99	y
Ma[176]	NSCLC	n	265	plat+docet+TNF	combo- other	8.7	8.5	9	0.99	1 phase	y	98.1	88.6	100	8.4	7.5	9.4	2.20E+11	6.6	infin	0.99	y
Mitsudomi[177]	NSCLC	n	86	cisp+docet	combo- other	6.3	5.7	6.9	0.84	Con-mod	n											n
Novello[178]	NSCLC	n	178	gem+cisp	combo- other	4.3	4.1	4.5	0.94	Con-low	n											n
Novello[178]	NSCLC	n	182	gem+cisp+iniparib	combo- other	6.1	5.7	6.4	0.92	Con-low	n											n
Novello[179]	NSCLC	n	80	motes+carbo+ paclit	combo- other	4.6	4.4	4.9	0.95	Con-mod	n											n
Patel[180]	NSCLC	n	467	paclit+carbo+bev	combo- other	5.4	5.1	5.8	0.95	Con-mod	n											n
Patel[180]	NSCLC	n	472	pemet+carbo+bev	combo- other	6.2	6	6.4	0.99	1 phase	n											n
Paz-Ares[181]	NSCLC	n	385	soraf+gem+cisp	combo- other	5.9	5.6	6.3	0.96	Con-mod	n											n
Paz-Ares[181]	NSCLC	n	387	gem+cisp	combo- other	4.9	4.7	5.3	0.90	S	n											n
Ramlau[182]	NSCLC	y	456	aflib+docet	combo- other	4.9	4.7	5.1	0.99	1 phase	n											n
Reck[183]	NSCLC	n	345	cisp+gem+bev low dose	combo- other	6.3	5.7	7	0.86	Con-high	n											n
Reck[183]	NSCLC	n	347	cisp+gem	combo- other	4.9	4.5	5.4	0.91	Con-high	n											n
Reck[183]	NSCLC	n	351	cisp+gem+bev high dose	combo- other	5.9	5.4	6.3	0.91	Con-high	n											n
Rosell[184]	NSCLC	n	87	plat combo	combo- other	4.9	4.5	5.3	0.86	Con-mod	n											n
Smit[90]	NSCLC	n	119	carbo+pemet	combo- other	3.7	3.5	3.9	0.94	Con-mod	n											n
Takeda[185]	NSCLC	n	298	plat combo 6 cycles	combo- other	3.9	3.5	4.3	0.96	Con-mod	n											n
Tsukada[186]	NSCLC	n	63	docet+cisp	combo- other	5.6	5	6.2	0.88	Con-mod	n											n
Wu[187]	NSCLC	n	188	carbo+paclit	combo- other	5.9	5.5	6.4	0.85	Con-mod	n											n
Wu[188]	NSCLC	n	107	gem+cisp	combo- other	5.5	5.1	6	0.85	Con-mod	n											n
Zhou[189]	NSCLC	n	82	carbo+gem	combo- other	4.6	4.3	4.9	0.87	Con-mod	n											n
Zukin[93]	NSCLC	n	103	carbo+pemet	combo- other	5.3	5.1	5.5	0.99	S	n											n
Mok[190]	NSCLC adeno nonsmoker	n	609	carbo+paclit	combo- other	5.1	4.6	5.6	0.87	Con-high	n											n
Solomon[39]	NSCLC ALK+	n	171	plat+pemet	combo- other	6	5.6	6.4	0.93	Con-mod	n											n

Continuation of Supplementary Online Table 2. Exponential decay nonlinear regression analysis models for different therapies and references for sources of published PFS curves

author	Tumor type	Prior Rx	no. pts	Rx	class Rx	PFS $t_{1/2}$ mo.	LCI mo	UCI mo	R^2	PFS curve shape	2-phase by NLRA	% fast	LCI %	UCI %	fast $t_{1/2}$ mo.	LCI mo	UCI mo	slow $t_{1/2}$ mo.	LCI mo	UCI mo	R2	2 phase by shape or NLRA
Gridelli[191]	NSCLC EGFR WT	n	117	gem+cisp	combo- other	4.9	4.7	5.1	0.97	Con-low	n											n
Mok[190]	NSCLC EGFR WT	n	85	carbo+paclit	combo- other	5.6	5	6.2	0.85	Con-high	n											n
Maemondo[192]	NSCLC EGFR-m	n	114	carbo+paclit	combo- other	4.7	4.3	5.2	0.84	Con-high	n											n
Mok[190]	NSCLC EGFR-m	n	129	carbo+paclit	combo- other	5.7	5.1	6.4	0.83	Con-high	n											n
Sequist[193]	NSCLC EGFR-m	n	115	cisp+pemet	combo- other	6.5	6.1	6.9	0.95	Con-mod	n											n
Gridelli[191]	NSCLC EGFR-u	n	380	cisp+gem	combo- other	5	4.8	5.2	0.97	2 phase	n											y
Scagliotti[194]	NSCLC nonsqu	n	618	pemet+cisp	combo- other	4.6	4.2	4.9	0.96	Con-mod	n											n
Scagliotti[194]	NSCLC nonsqu	n	634	gem+cisp	combo- other	4.1	3.8	4.4	0.96	Con-mod	n											n
Scagliotti[195]	NSCLC nonsqu	n	541	carbo+paclit	combo- other	4.3	4	4.6	0.96	Con-mod	n											n
Scagliotti[195]	NSCLC nonsqu	n	549	motes+carbo+ paclit	combo- other	5.1	4.8	5.4	0.97	S	n											n
Reck[196]	NSCLC PDL1+	n	151	plat combo	combo- other	4.8	4.6	5	0.95	S	n											n
Scagliotti[194]	NSCLC squam	n	229	gem + cispl	combo- other	4.5	4.2	4.8	0.96	Con-mod	n											n
Scagliotti[194]	NSCLC squam	n	244	pemet+cisp	combo- other	3.7	3.4	3.9	0.97	Con-high	n											n
Pujade-Lauraine[62]	ovary	y	179	bev+PEG doxo or paclit or topot	combo- other	6.6	6.1	7.1	0.94	Con-mod	n											n
Borad[197]	pancreas	n	76	TH-302 240 mg+ gem	combo- other	4.8	4.6	5.1	0.97	S	n											n
Borad[197]	pancreas	n	80	TH-302 340 mg+ gem	combo- other	5.5	5.3	5.7	0.97	S	n											n
Conroy[198]	pancreas	n	171	FOLFIRINOX	combo- other	5.4	5.1	5.7	0.95	Con-mod	n											n
Cunningham[199]	pancreas	n	267	cape+gem	combo- other	4.9	4.7	5.2	0.98	Con-low	n											n
Moore[200]	pancreas	n	285	erlot+gem	combo- other	3.9	3.8	4.1	0.97	Con-low	n											n
Ueno[201]	pancreas	n	275	S1+gem	combo- other	5.5	5.4	5.7	0.99	1 phase	n											n
Van Cutsem[202]	pancreas	n	301	gem+erlot	combo- other	3.2	3	3.5	0.96	Con-low	n											n
Van Cutsem[202]	pancreas	n	306	bev+gem+erlot	combo- other	4.1	3.9	4.4	0.98	Con-low	n											n
von Hoff[203]	pancreas	n	431	nab-paclit+gem	combo- other	5.2	5	5.4	0.97	Con-low	n											n
Kelly[204]	prostate	y	524	bev+docet+pred	combo- other	9	8.6	9.5	0.96	Con-mod	n											n
Kelly[204]	prostate	y	526	docet+pred	combo- other	7	6.8	7.3	0.99	1 phase	n											n
Sweeney[70]	Prostate	n	397	docet+anitandro	combo- other	37	36	38	0.98	1 phase	y	84.7	75.9	93.6	28.8	24.7	34.4	8.40E+12	377	infin	0.98	y
Escudier[205]	renal	n	327	bev+IFN	combo- other	9.1	8.8	9.4	0.98	Con-low	n											n
Rini[206]	renal	n	369	bev+IFN	combo- other	8.3	8.1	8.5	0.99	1 phase	n											n
Reck[123]	SCLC	n	476	plat+etop	combo- other	4.3	3.9	5	0.78	Con-high	n											n
Satouchi[207]	SCLC	n	142	irino+cisp	combo- other	5.5	4.8	6.6	0.78	Con-high	n											n
Satouchi[207]	SCLC	n	142	amrub+cisp	combo- other	4.6	3.9	5.5	0.71	Con-high	n											n
Socinski[208]	SCLC	n	453	pemet+carbo	combo- other	4.2	4	4.5	0.94	S	n											n

Continuation of Supplementary Online Table 2. Exponential decay nonlinear regression analysis models for different therapies and references for sources of published PFS curves

author	Tumor type	Prior Rx	no. pts	Rx	class Rx	PFS $t_{1/2}$ mo.	LCI mo	UCI mo	R^2	PFS curve shape	2-phase by NLRA	% fast	LCI %	UCI %	fast $t_{1/2}$ mo.	LCI mo	UCI mo	slow $t_{1/2}$ mo.	LCI mo	UCI mo	R2	2 phase by shape or NLRA
Socinski[208]	SCLC	n	455	etop+carbo	combo- other	6.3	5.8	7	0.86	Con-high	n											n
Tiseo[209]	SCLC	n	101	bev+cisp+etop	combo- other	6.3	5.7	6.9	0.89	Con-high	n											n
Tiseo[209]	SCLC	n	103	cisp+etop	combo- other	5.4	4.9	5.9	0.90	Con-mod	n											n
Schmid[210]	breast	n	451	atezo+chemo	combo PD1/chem	6.9	6.4	7.3	0.95	Con-low	n											n
Schmid[210]	breast PDL1+	n	185	atezo+chemo	combo PD1/chem	7.5	7.3	7.8	0.99	S	n											n
Gandhi[211]	NSCLC	n	410	pembro+chemo	combo PD1/chem	8.6	8.4	8.8	0.98	1 phase	n											n
Paz-Ares[212]	NSCLC	n	278	pembro+chemo	combo PD1/chem	7.7	7.3	8.1	0.94	Con-low	n											n
Socinski[213]	NSCLC	n	356	atezo+chemo	combo PD1/chem	9	8.8	9.3	0.97	S	n											n
Hodi[214]	melanoma	n	95	ipi+nivol	combo PD1/ipi	16.6	15.1	18.2	0.58	2 phase	y	51.2	38.6	63.8	4.1	3.2	5.7	3.70E+15	?	?	0.97	y
Larkin[215]	melanoma	n	314	ipi+nivol	combo PD1/ipi	11.3	10.9	11.8	0.93	2 phase	y	55.8	28.3	63.4	4.5	2.7	5.4	146.2	22.1	?	0.98	y
Long[216]	melanoma	y	153	ipi+pembro	combo PD1/ipi	21.5	19.9	23.2	0.6	2 phase	y	33.4	25.7	37.9	3.2	2.3	?	268.5	76.6	?	0.95	y
Postow[217]	melanoma	n	72	ipi+nivol	combo PD1/ipi	12.2	11.7	12.7	0.93	2 phase	y	86.3	64.2	100	10	7.3	15.9	2.00E+12	32.3	infin	0.95	y
Tawbi[218]	melanoma	n	94	ipi+nivol	combo PD1/ipi	12.8	11.7	13.9	0.51	2 phase	y	45.1	36.7	49.4	2.3	1.9	2.7	171.6	66.5	?	0.97	y
Wolchok[219]	melanoma	n	314	ipi+nivol	combo PD1/ipi	18.7	17.1	20.5	0.6	2 phase	y	50.1	47.5	52.9	3.6	3.3	3.9	100.9	80.6	138.7	0.99	y
Tawbi[218]	melanoma†	n	94	ipi+nivol	combo PD1/ipi	14.5	13.2	16	0.36	2 phase	y	43.4	38.7	44.7	2.3	2	2.5	1698	121	?	0.97	y
Tawbi[218]	mel-extracran	n	94	ipi+nivol	combo PD1/ipi	21.3	20	22.8	0.63	2 phase	y	57.7	?	60.5	9.9	?	?	7.30E+12	22.8	?	0.79	y
Motzer[220]	renal	n	425	ipi+nivol	combo PD1/ipi	12.7	11.9	13.6	0.91	2 phase	y	36.7	27.1	51.6	3.3	2.4	4.6	26.8	21.1	46.4	0.99	y
Hellmann[221]	TMB high	n	63	ipi+nivol	combo PD1/ipi	8.8	8.2	9.5	0.87	2 phase	y	95.5	?	99.9	8.5	?	?	1.40E+12	8.3	?	0.88	y
Aghajanian[222]	ovary	y	238	gem+carbo	combo plat ovary	8.6	7.7	9.7	0.78	Con-mod	n											n
Aghajanian[222]	ovary	y	241	bev+gem+carbo	combo plat ovary	15.4	13.7	17.3	0.78	Con-high	n											n
Burger[223]	ovary	n	623	bev throughout carbo+paclit	combo plat ovary	15.1	14.4	15.9	0.95	Con-mod	n											n
Burger[223]	ovary	n	625	carbo+paclit	combo plat ovary	12	11.5	12.5	0.94	S	n											n
Du Bois[224]	ovary	n	860	carbo+paclit+gem	combo plat ovary	20.1	19.2	21	0.96	1 phase	y	95.7	87.7	100	18.5	?	?	4.70E+12	41.1	infin	0.96	y
Du Bois[224]	ovary	n	882	carbo+paclit	combo plat ovary	23.8	22.7	24.9	0.96	1 phase	y	89.2	0	100	19.4	10.1	263.4	5.90E+15	?	?	0.97	y
Perren[225]	ovary	n	745	bev+carbo	combo plat ovary	21.2	19.6	23.1	0.86	Con-high	n											n
Chapman[95]	melanoma	n	338	dacarbazine	dacarbazine	2	1.8	2.1	0.89	S	n											n
Demetri[226]	sarcoma	y	173	dacarbazine	dacarbazine	2.3	2.1	2.4	0.91	1 phase	n											n
Flaherty[227]	melanoma B-m	n	108	dacarbazine	dacarbazine	2.1	1.9	2.3	0.87	S	n											n

Continuation of Supplementary Online Table 2. Exponential decay nonlinear regression analysis models for different therapies and references for sources of published PFS curves

author	Tumor type	Prior Rx	no. pts	Rx	class Rx	PFS $t_{1/2}$ mo.	LCI mo	UCI mo	R^2	PFS curve shape	2-phase by NLRA	% fast	LCI %	UCI %	fast $t_{1/2}$ mo.	LCI mo	UCI mo	slow $t_{1/2}$ mo.	LCI mo	UCI mo	R2	2 phase by shape or NLRA
Robert[124]	melanoma	n	252	dacarbazine	dacarbazine	3.1	2.9	3.4	0.91	2 phase	y	93.8	71.5	100	2.7	2.1	3.8	4.70E+15	?	?	0.92	y
Robert[228]	melanoma B-WT	n	208	dacarbazine	dacarbazine	2.8	2.6	3.1	0.89	S	n											n
Cunningham[229]	colon	y	111	cetuximab	EGFR mono	1.9	1.7	2	0.82	Con-mod	n											n
Hecht[230]	colon	y	388	panitumumab	EGFR mono	2.5	2.2	2.9	0.9	Con-mod	n											n
Muro[231]	colon	y	52	panitumumab	EGFR mono	2.5	2.2	2.8	0.72	Con-high	n											n
Siu[152]	colon	y	374	cetuximab	EGFR mono	3.2	3	3.5	0.95	Con-mod	n											n
Van Cutsem[232]	colon	y	231	panitumumab	EGFR mono	2.4	2.2	2.6	0.90	S	n											n
Van Cutsem[233]	colon	y	176	panitumumab	EGFR mono	2.6	2.4	2.8	0.92	Con-mod	n											n
Hecht[234]	colon EG high	y	105	panitumumab	EGFR mono	3.2	3	3.4	0.94	1 phase	n											n
Jonker[235]	colon EG IHC+	y	287	cetuximab	EGFR mono	2.5	2.3	2.7	0.93	Con-low	n											n
Amado[236]	colon KRAS WT	y	124	panitumumab	EGFR mono	3.1	3	3.2	0.96	Con-low	n											n
Karapetis[237]	colon KRAS WT	y	110	cetuximab	EGFR mono	3.6	3.4	3.8	0.92	Con-mod	n											n
Amado[236]	colon KRAS-m	y	84	panitumumab	EGFR mono	2.1	1.7	2.7	0.44	Con-high	n											n
Zhang[238]	colon T-T geno	y	62	cetuximab	EGFR mono	1.6	1.4	1.7	0.86	S	n											n
Machiels[239]	head/neck	y	191	zalutumab	EGFR mono	2.8	2.7	2.9	0.94	S	n											n
Vermorkan[240]	head/neck	y	103	cetuximab	EGFR mono	2.4	2.3	2.6	0.89	Con-mod	n											n
Cheng[241]	NSCLC EGFR-m	n	65	gefitinib	EGFR TKI-m	11.1	10.4	7.8	0.92	Con-mod	n											n
Douillard[242]	NSCLC EGFR-m	n	106	gefitinib	EGFR TKI-m	9.8	9.4	10.4	0.91	Con-mod	n											n
Goto[243]	NSCLC EGFR-m	n	102	erlotinib	EGFR TKI-m	12.8	12.3	13.3	0.93	Con-mod	n											n
Maemondo[192]	NSCLC EGFR-m	n	114	gefitinib	EGFR TKI-m	10.1	9.7	10.5	0.95	Con-mod	n											n
Mitsudomi[177]	NSCLC EGFR-m	n	86	gefitinib	EGFR TKI-m	10.4	9.6	11.3	0.85	Con-mod	n											n
Mok[190]	NSCLC EGFR-m	n	132	gefitinib	EGFR TKI-m	8.1	7.6	8.7	0.91	Con-mod	n											n
Park[244]	NSCLC EGFR-m	n	159	gefitinib	EGFR TKI-m	9.5	9.1	10.1	0.91	Con-mod	n											n
Park[244]	NSCLC EGFR-m	n	160	afatinib	EGFR TKI-m	11.3	10.9	11.7	0.96	Con-low	n											n
Rosell[184]	NSCLC EGFR-m	n	86	erlotinib	EGFR TKI-m	10.2	9.8	10.6	0.95	Con-low	n											n
Sequist[193]	NSCLC EGFR-m	n	230	afatinib	EGFR TKI-m	10.3	9.9	10.7	0.97	Con-low	n											n
Seto[245]	NSCLC EGFR-m	n	77	erlotinib	EGFR TKI-m	9.5	9.4	9.7	0.99	1 phase	n											n
Soria[246]	NSCLC EGFR-m	n	277	standard TKIs	EGFR TKI-m	10.7	10.3	11.3	0.95	Con-mod	n											n
Soria[246]	NSCLC EGFR-m	n	279	osimertinib	EGFR TKI-m	21.1	20.2	22	0.94	Con-low	n											n
Urata[247]	NSCLC EGFR-m-L858R	y	80	erlotinib	EGFR TKI-m	8	7.6	8.4	0.97	Con-low	n											n
Urata[247]	NSCLC EGFR-m-exon 19	y	90	gefitinib	EGFR TKI-m	10.4	10	10.8	0.98	Con-low	n											n

Continuation of Supplementary Online Table 2. Exponential decay nonlinear regression analysis models for different therapies and references for sources of published PFS curves																						
author	Tumor type	Prior Rx	no. pts	Rx	class Rx	PFS $t_{1/2}$ mo.	LCI mo	UCI mo	R^2	PFS curve shape	2-phase by NLRA	% fast	LCI %	UCI %	fast $t_{1/2}$ mo.	LCI mo	UCI mo	slow $t_{1/2}$ mo.	LCI mo	UCI mo	R2	2 phase by shape or NLRA
Urata[247]	NSCLC EGFR-m-L858R	y	92	gefitinib	EGFR TKI-m	7.7	7.5	8	0.99	1 phase	n											n
Urata[247]	NSCLC EGFR-m-exon 19	y	102	erlotinib	EGFR TKI-m	10.7	10.1	11.5	0.95	S	n											n
Urata[247]	NSCLC EGFR-m	y	198	erlotinib	EGFR TKI-m	9.3	8.9	9.8	0.97	S	n											n
Urata[247]	NSCLC EGFR-m	y	203	gefitinib	EGFR TKI-m	8.6	8.3	8.8	0.99	Con-low	n											n
Wu[248]	NSCLC EGFR-m	n	242	afatinib	EGFR TKI-m	11.1	10.5	11.7	0.93	Con-mod	n											n
Wu[188]	NSCLC EGFR-m	n	110	erlotinib	EGFR TKI-m	12.4	11.7	13.2	0.89	Con-low	n											n
Wu[249]	NSCLC EGFR-m	n	225	gefitinib	EGFR TKI-m	9.6	8.9	10.3	0.91	Con-mod	n											n
Wu[249]	NSCLC EGFR-m	n	227	dacomitinib	EGFR TKI-m	14.8	14.4	15.3	0.98	S	n											n
Zhou[189]	NSCLC EGFR-m	n	83	erlotinib	EGFR TKI-m	14.2	13.7	14.7	0.94	Con-low	n											n
Aerts[250]	NSCLC EGFR-u	y	115	erlotinib	EGFR TKI-u	4.9	4.9	5	0.99	1 phase	n											n
Ahn[251]	NSCLC EGFR-u	Mnt	75	vandetanib	EGFR TKI-u	3.2	3.1	3.3	0.98	2 phase	y	86.7	22.6	100	2.6	1.7	5.1	16.5	1.7	infin	0.98	y
Cappuzzo[252]	NSCLC EGFR-u	Mnt	438	erlotinib	EGFR TKI-u	3.5	3.3	3.6	0.96	2 phase	y	95.5	69.2	100	3.1	2.4	4.3	6.00E+15	?	?	0.97	y
Chen[253]	NSCLC EGFR-u	y	58	gefitinib	EGFR TKI-u	5.4	5.2	5.5	0.97	S	n											n
Chen[254]	NSCLC EGFR-u	n	57	erlotinib	EGFR TKI-u	4.9	4.7	5	0.98	2 phase	y	91.9	80.6	100	4	3.5	4.6	4.60E+15	?	?	0.99	y
Cicenas[255]	NSCLC EGFR-u	Mnt	322	erlotinib	EGFR TKI-u	3.3	3.1	3.6	0.95	2 phase	n											y
Ciuleanu[256]	NSCLC EGFR-u	y	203	erlotinib	EGFR TKI-u	6.8	6.3	7.5	0.9	S	y	93.2	81.9	100	1.4	1.2	1.7	4.80E+10	4.2	infin	0.97	y
Crino[257]	NSCLC EGFR-u	n	97	gefitinib	EGFR TKI-u	2.2	2.2	2.3	0.97	1 phase	n											n
Ellis[258]	NSCLC EGFR-u	y	480	dacomitinib	EGFR TKI-u	3.5	3.4	3.7	0.97	2 phase	y	88.7	84.8	92.7	2.7	2.5	3	2.70E+12	29.8	infin	0.98	y
Fukuoka[259]	NSCLC EGFR-u	y	208	gefitinib	EGFR TKI-u	2.8	2.6	2.9	0.93	S	n											n
Gaafer[260]	NSCLC EGFR-u	Mnt	86	gefitinib	EGFR TKI-u	4.6	4.5	4.8	0.98	2 phase	y	93.9	57.9	100	4.1	3	6.2	4.80E+15	?	?	0.98	y
Gemma[261]	NSCLC EGFR-u	y	10,708	erlotinib	EGFR TKI-u	2.7	2.6	2.8	0.97	2 phase	y	70.1	58.9	80 1. 4	1.6	1.5	1.9	7.4	5.4	11.5	0.99	y
Goss[262]	NSCLC EGFR-u	n	100	gefitinib	EGFR TKI-u	1.6	1.6	1.7	0.97	1 phase	n											n
Gridelli[191]	NSCLC EGFR-u	n	380	erlotinib	EGFR TKI-u	3	2.8	3.2	0.95	2 phase	y	95.9	81.1	100	2.7	2.3	3.4	1.30E+16	?	?	0.95	y
Groen[263]	NSCLC EGFR-u	y	67	erlotinib	EGFR TKI-u	2.7	2.5	2.9	0.9	S	n											n
Han[264]	NSCLC EGFR-u	y	54	gefitinib	EGFR TKI-u	3.2	3	3.4	0.95	2 phase	y	57.2	6.5	100	1.9	1.2	4.5	6.2	3.5	25	0.96	y
Han[170]	NSCLC EGFR-u	n	159	gefitinib	EGFR TKI-u	5	4.9	5.1	0.99	S	n											n
Heigener[171]	NSCLC EGFR-u	n	144	erlotinib	EGFR TKI-u	3.1	2.9	3.4	0.89	S	n											n
Herbst[265]	NSCLC EGFR-u	y	317	erlotinib	EGFR TKI-u	2.4	2.2	2.5	0.94	2 phase	n											n

Continuation of Supplementary Online Table 2. Exponential decay nonlinear regression analysis models for different therapies and references for sources of published PFS curves

author	Tumor type	Prior Rx	no. pts	Rx	class Rx	PFS $t_{1/2}$ mo.	LCI mo	UCI mo	R^2	PFS curve shape	2-phase by NLRA	% fast	LCI %	UCI %	fast $t_{1/2}$ mo.	LCI mo	UCI mo	slow $t_{1/2}$ mo.	LCI mo	UCI mo	R2	2 phase by shape or NLRA
Jackman[266]	NSCLC EGFR-u	n	80	erlotinib	EGFR TKI-u	4.5	4.2	4.7	0.93	2 phase	y	78.3	62.9	93.8	2.8	2.3	3.5	24	10.6	infin	0.98	y
Janne[267]	NSCLC EGFR-u	n	81	erlotinib	EGFR TKI-u	6.1	5.7	6.4	0.94	2 phase	y	51	42.3	61	2.4	2	2.8	13.3	11.3	16.9	0.99	y
Karampeazis[84]	NSCLC EGFR-u	y	166	erlotinib	EGFR TKI-u	3.9	3.7	4	0.98	2 phase	y	94.8	91.6	98	3.5	3.3	3.7	3.80E+12	29.8	infin	0.99	y
Kawaguchi[268]	NSCLC EGFR-u	y	151	erlotinib	EGFR TKI-u	2.6	2.4	2.9	0.93	2 phase	y	72	42.8	100	1.5	1.1	2.5	9.8	4.3	infin	0.97	y
Kim[269]	NSCLC EGFR-u	y	733	gefitinib	EGFR TKI-u	2.7	2.6	2.8	0.97	2 phase	y	95.6	83.2	100	2.4	2.1	2.9	5.30E+15	?	?	0.97	y
Kim[270]	NSCLC EGFR-u	y	80	gefitinib	EGFR TKI-u	3.4	3.3	3.6	0.96	2 phase	y	88.5	82.2	96.5	2.8	2.4	3.3	1.10E+11	3.7	?	0.96	y
Lee[271]	NSCLC	y	617	vandetanib	EGFR TKI-u	2.6	2.5	2.9	0.93	S	n											n
Lee[272]	NSCLC EGFR-u	n	350	erlotinib	EGFR TKI-u	2.8	2.7	2.9	0.96	2 phase	y	95.1	90	100	2.5	2.3	2.7	5.10E+15	?	?	0.99	y
Lee[87]	NSCLC EGFR-u	y	82	erlotinib	EGFR TKI-u	4.8	4.5	5	0.97	2 phase	y	40	22.4	42.4	1.5	1.1	1.9	7	6.2	8	0.98	y
Lilenbaum[175]	NSCLC EGFR-u	n	52	erlotinib	EGFR TKI-u	1.6	1.5	1.6	0.99	2 phase	n											y
Maruyama[273]	NSCLC EGFR-u	y	200	gefitinib	EGFR TKI-u	2.7	2.5	2.8	0.94	2 phase	y	95.1	61.4	100	2.4	1.8	3.7	3.40E+15	?	?	0.94	y
Mok[190]	NSCLC adeno nonsmoker	n	608	gefitinib	EGFR TKI-u	5.8	5.7	5.9	0.99	Con-low	n											n
Mok[274]	NSCLC EGFR-u-Asian	y	1242	erlotinib	EGFR TKI-u	6.8	6.5	7	0.98	2 phase	y	34.8	27.6	42	2.3	1.9	2.9	10.3	9.5	11.2	0.99	y
Mok[274]	NSCLC EGFR-u non-Asian	y	5338	erlotinib	EGFR TKI-u	3.5	3.3	3.7	0.97	2 phase	y	85.3	76.8	93.8	2.7	2.4	3	19.4	11.4	65.2	0.99	y
Mok[275]	NSCLC EGFR-u	n	94	gefitinib	EGFR TKI-u	5.1	4.9	5.2	0.98	2 phase	y	93.1	53.2	95.8	4.4	3.3	?	4.70E+15	?	?	0.99	y
Natale[276]	NSCLC EGFR-u	y	83	vandetanib	EGFR TKI-u	2.5	2.4	2.6	0.95	1 phase	n											n
Natale[276]	NSCLC EGFR-u	y	85	gefitinib	EGFR TKI-u	1.9	1.7	2	0.92	2 phase	n											y
Natale[277]	NSCLC EGFR-u	y	617	erlotinib	EGFR TKI-u	2.9	2.8	3.1	0.97	2 phase	y	93.6	74.5	100	2.5	2.1	3.2	81.4	3.4	infin	0.97	y
Natale[277]	NSCLC EGFR-u	y	623	vandetanib	EGFR TKI-u	2.9	2.8	3	0.99	2 phase	y	79.5	44.4	100	2.3	1.8	3	6.9	3.3	infin	0.99	y
Perng[278]	NSCLC EGFR-u	y	299	erlotinib	EGFR TKI-u	6.1	6	6.3	0.98	1 phase	y	25.1	14.5	35.6	2	1.5	3.1	8.1	7.3	9.1	0.99	y
Perol[279]	NSCLC	Mnt	155	erlotinib	EGFR TKI-u	3.1	2.8	3.4	0.93	2 phase	y	92.1	85.2	95	2.6	2.3	?	4.70E+15	?	?	0.95	y
Ramalingam[280]	NSCLC EGFR-u	y	57	erlotinib	EGFR TKI-u	2.6	2.4	2.7	0.92	2 phase	y	89.8	79	100	2.1	?	?	7.60E+11	8.1	infin	0.87	y
Ramalingam[281]	NSCLC EGFR-u	y	94	erlotinib	EGFR TKI-u	2.6	2.5	2.8	0.91	2 phase	n											y
Ramalingam[281]	NSCLC EGFR-u	y	94	dacomitinib	EGFR TKI-u	3.6	3.4	3.8	0.94	2 phase	y	90.8	82.3	99.4	2.9	2.6	3.3	3.20E+15	?	?	0.97	y
Ramalingam[282]	NSCLC EGFR-u	y	439	erlotinib	EGFR TKI-u	2.9	2.8	3	0.96	2 phase	y	96.1	65.2	100	2.7	2	3.8	3.70E+15	?	?	0.96	y
Ramalingam[282]	NSCLC EGFR-u	y	439	dacomitinib	EGFR TKI-u	3.1	2.9	3.2	0.87	2 phase	y	93	68	100	2.6	2	3.7	3.80E+15	?	?	0.95	y
Reck[283]	NSCLC EGFR-u	n	58	gefitinib	EGFR TKI-u	2.5	2.4	2.7	0.9	2 phase	y	89.6	58.6	100	2.1	?	?	7.40E+10	2.3	infin	0.91	y
Reck[284]	NSCLC EGFR-u	y	5394	erlotinib	EGFR TKI-u	4	3.9	4.2	0.97	2 phase	y	76	68	83.9	2.7	2.4	3	14	10.7	20.1	0.99	y
Scagliotti[101]	NSCLC	y	480	erlotinib	EGFR TKI-u	3.1	3	3.3	0.95	2 phase	y	96.6	80.2	100	2.9	2.4	3.7	4.50E+10	2.8	infin	0.95	y

Continuation of Supplementary Online Table 2. Exponential decay nonlinear regression analysis models for different therapies and references for sources of published PFS curves

author	Tumor type	Prior Rx	no. pts	Rx	class Rx	PFS $t_{1/2}$ mo.	LCI mo	UCI mo	R^2	PFS curve shape	2-phase by NLRA	% fast	LCI %	UCI %	fast $t_{1/2}$ mo.	LCI mo	UCI mo	slow $t_{1/2}$ mo.	LCI mo	UCI mo	R2	2 phase by shape or NLRA
Scagliotti[102]	NSCLC nonsqu	y	522	erlotinib	EGFR TKI-u	2.5	2.3	2.7	0.93	2 phase	y	96.2	57.2	100	2.3	1.6	4	3.40E+15	?	?	0.93	y
Scagliotti[285]	NSCLC EGFR-u	y	290	erlotinib	EGFR TKI-u	2.6	2.5	2.7	0.97	2 phase	n											y
Schneider[286]	NSCLC EGFR-u	y/n	239	erlotinib	EGFR TKI-u	2.8	2.7	2.9	0.99	2 phase	y	93.3	85	100	2.5	2.3	2.8	20.2	6.9	infin	0.99	y
Sequist[287]	NSCLC EGFR-u	y	83	erlotinib	EGFR TKI-u	2.9	2.8	3	0.94	2 phase	n											y
Shepherd[288]	NSCLC EGFR-u	y	731	erlotinib	EGFR TKI-u	3.2	3	3.3	0.97	2 phase	n											y
Simon[289]	NSCLC EGFR-u	y/n	183	gefitinib	EGFR TKI-u	5.6	5.4	5.9	0.93	2 phase	y	93.1	52.7	100	4.8	3.4	8.2	6.20E+15	?	?	0.94	y
Soria[290]	NSCLC squam	y	397	erlotinib	EGFR TKI-u	2.7	2.6	3	0.89	2 phase	n											y
Soria[290]	NSCLC squam	y	398	afatinib	EGFR TKI-u	3.4	3.2	3.6	0.93	2 phase	n											y
Spigel[291]	NSCLC EGFR-u	y	55	erlotinib	EGFR TKI-u	2.9	2.7	3	0.94	2 phase	y	93.1	74.3	100	2.4	2	3.1	3.90E+15	?	?	0.95	y
Stinchcombe[292]	NSCLC EGFR-u	n	51	erlotinib	EGFR TKI-u	2.8	2.7	3	0.97	2 phase	y	90.2	11.3	?	2.4	1	?	5.50E+15	?	?	0.98	y
Sun[91]	NSCLC EGFR-u	y	68	gefitinib	EGFR TKI-u	8.1	7.9	8.4	0.97	2 phase	y	10.2	3.4	17	1.1	0.6	11.2	9.4	8.6	10.4	0.98	y
Takeda[185]	NSCLC EGFR-u	y	300	gefitinib	EGFR TKI-u	5.3	5	5.7	0.97	2 phase	y	95	87.9	96.6	4.7	4.2	?	3.50E+15	?	?	0.98	y
Thatcher[293]	NSCLC EGFR-u	y	1129	gefitinib	EGFR TKI-u	3.4	3.3	3.5	0.99	2 phase	y	93.9	81.1	100	3	2.6	3.4	4.60E+15	?	?	0.99	y
Tiseo[294]	NSCLC EGFR-u	y	651	erlotinib	EGFR TKI-u	4	3.8	4.1	0.98	2 phase	y	96	93.8	98.3	3.6	3.4	3.8	5.00E+12	25.8	infin	0.98	y
Uhm[295]	NSCLC EGFR-u	y	120	erlotinib	EGFR TKI-u	3.9	3.7	4.2	0.9	2 phase	y	65.7	55.8	75.6	1.9	1.6	2.3	12.9	9.7	19.2	0.98	y
Urata[247]	NSCLC EGFR-u	y	279	erlotinib	EGFR TKI-u	7.2	7	7.4	0.99	2 phase	y	98.3	71.1	100	6.9	5.5	9.5	4.50E+15	?	?	0.99	y
Urata[247]	NSCLC EGFR-u	y	280	gefitinib	EGFR TKI-u	6.6	6.4	6.8	0.99	1 phase	y	13.7	1.2	26.3	1.8	1	8.7	7.6	6.9	8.5	0.99	y
Van Meerbeeck[296]	NSCLC EGFR-u	y	261	erlotinib	EGFR TKI-u	2.8	2.6	2.9	0.96	2 phase	n											y
West[297]	NSCLC EGFR-u	y	101	gefitinib	EGFR TKI-u	5.1	4.6	5.6	0.82	2 phase	y	73.5	65.7	81.4	2.4	2.1	2.9	34.9	21	104.9	0.96	y
Witta[298]	NSCLC EGFR-u	y	65	erlotinib	EGFR TKI-u	2.1	2	2.2	0.96	1 phase	n											n
Wu[187]	NSCLC EGFR-u	n	184	gefitinib	EGFR TKI-u	6.5	6.4	6.7	0.98	1 phase	n											n
Zhang[299]	NSCLC EGFR-u	Mnt	148	gefitinib	EGFR TKI-u	6.4	6.1	6.7	0.93	2 phase	y	32.5	23.7	41.2	1.6	1.2	2.3	10.2	9	11.9	0.98	y
Bell[300]	NSCLC EGFR WT	y	63	gefitinib	EGFR TKI-WT	2.1	2	2.2	0.94	1 phase	n											n
Cappuzzo[252]	NSCLC EGFR WT	Mnt	165	erlotinib	EGFR TKI-WT	2.8	2.7	3	0.93	1 phase	n											n
Choi[301]§	NSCLC EGFR WT	n	54	erlotinib	EGFR TKI-WT	0.7	0.7	0.8	0.95	2 phase	n											y
Ciuleanu[256]	NSCLC EGFR WT	y	75	erlotinib	EGFR TKI-WT	1.9	1.7	2.1	0.87	1 phase	n											n
Douillard[302]	NSCLC EGFR WT	y	106	gefitinib	EGFR TKI-WT	2	1.9	2.1	0.96	2 phase	y	95.4	86.8	100	1.8	1.6	2.1	3.50E+15	?	?	0.97	y
Garassino[303]	NSCLC EGFR WT	y	112	erlotinib	EGFR TKI-WT	2.4	2.3	2.5	0.98	1 phase	n											n
Gridelli[191]	NSCLC EGFR WT	n	119	erlotinib	EGFR TKI-WT	1.9	1.8	2	0.96	2 phase	n											y

Continuation of Supplementary Online Table 2. Exponential decay nonlinear regression analysis models for different therapies and references for sources of published PFS curves

author	Tumor type	Prior Rx	no. pts	Rx	class Rx	PFS $t_{1/2}$ mo.	LCI mo	UCI mo	R^2	PFS curve shape	2-phase by NLRA	% fast	LCI %	UCI %	fast $t_{1/2}$ mo.	LCI mo	UCI mo	slow $t_{1/2}$ mo.	LCI mo	UCI mo	R2	2 phase by shape or NLRA
Han[304]	NSCLC EGFR WT	y	73	gefitinib	EGFR TKI-WT	3.1	2.8	3.3	0.87	1 phase	n											n
Kawaguchi[268]	NSCLC EGFR WT	y	109	erlotinib	EGFR TKI-WT	2.2	2	2.6	0.85	1 phase	n											n
Li[88]	NSCLC EGFR WT	y	61	erlotinib	EGFR TKI-WT	4.9	4.7	5.1	0.98	1 phase	n											n
Mok[190]	NSCLC EGFR WT	n	91	gefitinib	EGFR TKI-WT	2.1	1.9	2.3	0.89	Con-low	n											n
Schneider[286]	NSCLC EGFR WT	y/n	68	erlotinib	EGFR TKI-WT	2	1.9	2.1	0.98	2 phase	y	93.2	87.9	98.4	1.7	1.6	1.9	3.70E+15	?	?	0.99	y
Spigel[291]	NSCLC EGFR WT	y	55	erlotinib	EGFR TKI-WT	1.9	1.7	2.2	0.7	S	n											n
Urata[247]	NSCLC EGFR WT	y	51	erlotinib	EGFR TKI-WT	2.4	2.1	2.8	0.9	2 phase	y	90	2.1	100	2	1	23.3	4.30E+15	?	?	0.91	y
Yoshioka[305]	NSCLC EGFR WT	y	153	erlotinib	EGFR TKI-WT	2.2	2.1	2.4	0.94	1 phase	y	62.1	0	100	1.5	0.6	infin	4.4	0.9	infin	0.95	y
Zhou[92]	NSCLC EGFR WT	y	81	gefitinib	EGFR TKI-WT	2.8	2.6	3	0.95	2 phase	y	57	25.5	89.5	1.6	0.9	2.3	5.6	3.8	23.4	0.97	y
Baselga[128]	breast	y	115	capecitabine	Fluoropyr	4.3	4.1	4.6	0.94	Con-mod	n											n
Sparano[133]	breast	y	612	capecitabine	Fluoropyr	4.7	4.5	4.8	0.99	S	n											n
Stockler[134]	breast	n	214	capecitabine	Fluoropyr	6.4	6.2	6.7	0.98	2 phase	y	92.5	88.8	93.9	5.3	5.1	5.6	3.20E+15	?	?	0.99	y
Mayer[306]	colon	y	534	TAS-102	Fluoropyr	2.5	2.3	2.7	0.95	1 phase	n											n
Tebbutt[154]	colon	n	156	capecitabine	Fluoropyr	5.2	4.9	5.5	0.96	Con-mod	n											n
Ueno[201]	pancreas	n	280	S1	Fluoropyr	3.4	3.2	3.5	0.99	S	n											n
Baselga[135]	breast ER+	y	239	exemestane	Fulvest	4.4	4.2	4.6	0.96	S	n											n
Baselga[307]	breast ER+/P-a	y	184	fulvestrant	Fulvest	5	4.7	5.2	0.94	2 phase	n											y
Baselga[307]	breast ER+	y	571	fulvestrant	Fulvest	5.4	5.2	5.6	0.97	2 phase	y	58.4	33.8	85	3	2.1	4	11.1	7.9	42.6	0.98	y
Chia[308]	breast ER+	y	305	exemestane	Fulvest	3.9	3.7	4.1	0.97	2 phase	n											y
Chia[308]	breast ER+	y	351	fulvestrant	Fulvest	4	3.8	4.2	0.97	2 phase	n											y
Cristofanilli[309]	breast ER+/P-WT	y	86	fulvestrant	Fulvest	5	4.7	5.2	0.94	1 phase	n											n
Cristofanilli[309]	breast ER+	y	174	fulvestrant	Fulvest	4.9	4.6	5.1	0.95	1 phase	n											n
Di Leo[310]	breast ER+	y	362	fulves 500 mg	Fulvest	8	7.6	8.4	0.91	2 phase	y	52.2	2.2	100	4.3	2.6	11.2	14.6	8.4	55.8	0.98	y
Di Leo[310]	breast ER+	y	374	fulves 250 mg	Fulvest	6	5.7	6.4	0.97	2 phase	y	92.2	71.3	100	5	4	6.6	1.40E+11	7.2	infin	0.98	y
Di Leo[311]	breast ER+/P-WT	y	69	fulvestrant	Fulvest	2.4	2.2	2.7	0.91	2 phase	n											y
Di Leo[311]	breast ER+	y	143	fulvestrant	Fulvest	2.7	2.5	3	0.88	2 phase	n											y
Ellis[1]	breast ER+	n	102	fulvestrant	Fulvest	20.5	19.6	21.6	0.92	1 phase	y	83.1	18.3	100	16.2	9.3	61	2.20E+11	12.2	infin	0.93	y
Howell[2]	breast PM	y	222	fulvestrant	Fulvest	5.7	5.5	6	0.97	2 phase	n											y
Howell[312]	breast ER+/?	n	313	fulvestrant	Fulvest	7.2	6.9	7.4	0.98	2 phase	y	32.4	6.8	?	3.4	1.4	?	10	7.7	?	0.98	y
Jeselsohn[313]	breast GSG2	y	73	fulvestrant	Fulvest	11.2	10.8	11.6	0.96	Con-low	n											n
Johnston[314]	breast ER+	y	231	fulvestrant	Fulvest	4.9	4.7	5.1	0.97	2 phase	n											y
Krop[315]	breast ER+	y	79	fulvestrant	Fulvest	5.3	5.1	5.5	0.98	2 phase	y	74.9	?	?	4.1	?	5.3	11.5	5.2	?	0.98	y
Ohno[316]	breast ER+	y	51	fulv loading dose	Fulvest	8.1	7.5	8.7	0.88	Con-low	n											n

Continuation of Supplementary Online Table 2. Exponential decay nonlinear regression analysis models for different therapies and references for sources of published PFS curves

author	Tumor type	Prior Rx	no. pts	Rx	class Rx	PFS $t_{1/2}$ mo.	LCI mo	UCI mo	R^2	PFS curve shape	2-phase by NLRA	% fast	LCI %	UCI %	fast $t_{1/2}$ mo.	LCI mo	UCI mo	slow $t_{1/2}$ mo.	LCI mo	UCI mo	R2	2 phase by shape or NLRA
Osborne[5]	breast PM	y	206	fulvestrant	Fulvest	5.7	5.5	6	0.97	2 phase	n											y
Perey[317]	breast PM	y	80	fulvestrant	Fulvest	4.1	3.8	4.4	0.94	2 phase	y	87.4	27.4	93.9	3.1	1.7	?	3.90E+15	?	?	0.96	y
Pritchard[318]	breast ER+	y	51	fulv loading dose	Fulvest	6.7	6.4	7.1	0.91	1 phase	y	93.1	77.7	?	6.1	4.5	7.1	3.60E+10	?	?	0.91	y
Robertson[6]	breast ER+	n	102	fulvestrant	Fulvest	20.6	19.6	21.6	0.92	2 phase	y	82.2	?	?	16.1	?	?	6.80E+11	?	?	0.93	y
Robertson[319]	breast ER+	n	230	fulvestrant	Fulvest	17	16.7	17.3	0.99	1 phase	n											n
Slamon[320]	breast ER+	y/n	242	fulvestrant	Fulvest	12.6	12.3	12.9	0.97	2 phase	y	9.4	7.4	11.9	1.1	0.8	1.6	14.7	14.1	15.4	0.99	y
Sledge[321]	breast ER+	y	223	fulvestrant	Fulvest	10.6	10.2	11.1	0.93	2 phase	y	53	30.8	74.4	4.4	2.8	6	33.9	18.1	?	0.98	y
Turner[322]	breast ER+	y	174	fulvestrant	Fulvest	4.4	4.1	4.7	0.92	S	n											n
Xu[7]	breast PM	y	121	fulvestrant	Fulvest	4.2	4	4.4	0.94	2 phase	y	95.9	86.5	?	3.9	3.2	4.4	5.20E+11	?	?	0.94	y
Zhang[323]	breast ER+	y	110	fulv-250 mg	Fulvest	5.5	5.1	6	0.91	2 phase	y	91.9	?	?	4.8	?	5.9	4.10E+15	?	?	0.91	y
Zhang[323]	breast ER+/PM	y	111	fulv-500 mg	Fulvest	8	7.7	8.4	0.96	2 phase	y	92.8	87.2	99.7	6.9	6.1	8	1.20E+10	8.1	?	0.96	y
Valle[126]	biliary	y	206	gemcitabine	Gem	4.7	4.5	5	0.96	Con-mod	n											n
Brodowicz[324]	NSCLC	Mnt	138	gemcitabine	Gem	3.8	3.7	4	0.97	2phase	n											y
Perol[279]	NSCLC	Mnt	154	gemcitabine	Gem	3.8	3.6	4	0.97	2 phase	n											y
Sederholm[325]	NSCLC	n	170	gemcitabine	Gem	3.8	3.6	4	0.94	Con-mod	n											n
Berlin[326]	pancreas	n	162	gemcitabine	Gem	2.8	2.6	2.9	0.92	Con-low	n											n
Borad[197]	pancreas	n	73	gemcitabine	Gem	3.4	3.2	3.6	0.97	S	n											n
Colucci[327]	pancreas	n	199	gemcitabine	Gem	4.5	4.2	4.8	0.96	2 phase	n											y
Conroy[198]	pancreas	n	171	gemcitabine	Gem	3	2.8	3.2	0.95	Con-mod	n											n
Cunningham[199]	pancreas	n	266	gemcitabine	Gem	4	3.8	4.3	0.97	Con-mod	n											n
Heinemann[328]	pancreas	n	97	gemcitabine	Gem	6.4	6.1	6.7	0.95	1 phase	n											n
Lee[329]	pancreas	n	106	gemcitabine	Gem	5	4.7	5.2	0.98	1 phase	n											n
Middleton[330]	pancreas	n	70	gemcitabine	Gem	6.4	6.2	6.6	0.98	Con-low	n											n
Moore[200]	pancreas	n	284	gemcitabine	Gem	3.3	3.1	3.5	0.96	Con-mod	n											n
Poplin[331]	pancreas	n	275	gemcitabine	Gem	2.8	2.7	3	0.98	2 phase	n											y
Poplin[331]	pancreas	n	277	fixed dose rate gem	Gem	3.3	3.1	3.4	0.98	1 phase	n											n
Rocha Lima[332]	pancreas	n	180	gemcitabine	Gem	3	2.9	3.2	0.98	1 phase	n											n
Ueno[201]	pancreas	n	277	gemcitabine	Gem	3.7	3.6	3.9	0.98	1 phase	n											n
von Hoff[203]	pancreas	n	430	gemcitabine	Gem	3.7	3.5	3.9	0.95	S	n											n
Nishida[333]	GIST	y/n	74	imatinib	GIST	24.1	23.1	25.1	0.95	Con-low	n											n
Casali[334]	GIST	y/n	473	imatinib 400 mg	GIST	23	21.9	24.2	0.98	2 phase	y	88.6	81.8	92.3	17.2	15.8	18.3	252	102	?	0.99	y
Casali[334]	GIST	y/n	473	imatinib 800 mg	GIST	26.1	25.2	26.9	0.99	2 phase	y	93.8	90.4	94.7	22.1	21.2	22.7	5.70E+15	?	?	0.99	y
Blanke[335]	GIST	y/n	345	imatinib-400 mg	GIST	19.8	19.3	20.2	0.99	2 phase	y	46.3	30	68.9	10.2	7.9	12.8	33.4	27.6	53.2	0.99	y
Blanke[335]	GIST	y/n	349	imatinib-800 mg	GIST	22.6	22.2	23	0.99	2 phase	y	66.1	22.7	89.5	14.8	8.5	18.3	54.3	29	?	0.99	y
Demetri[336]	GIST	y	133	regorafenib	GIST	4.5	4.3	4.6	0.96	1 phase	y	9.2	3.5	47.7	0.8	0.4	2.5	5.2	4.7	8.3	0.97	y
Demetri[337]	GIST	y	207	sunitinib	GIST	5.3	5	5.5	0.92	1 phase	y	98.8	54.7	?	5.2	?	?	9.20E+11	?	?	0.92	y
Komatsu[338]	GIST	y	470	sunitinib	GIST	6.4	6.1	6.6	0.96	1 phase	n											n
Reichardt[339]	GIST	y	1124	sunitinib	GIST	9.1	8.8	9.3	0.99	2 phase	y	90.6	80.6	95.7	7.6	6.9	8.1	48.4	24.3	?	0.99	y
Hurvitz[340]	breast HER2+	n	67	T-DM1	HER2 mono	14.7	14.3	15.1	0.98	1 phase	n											n

Continuation of Supplementary Online Table 2. Exponential decay nonlinear regression analysis models for different therapies and references for sources of published PFS curves

author	Tumor type	Prior Rx	no. pts	Rx	class Rx	PFS $t_{1/2}$ mo.	LCI mo	UCI mo	R^2	PFS curve shape	2-phase by NLRA	% fast	LCI %	UCI %	fast $t_{1/2}$ mo.	LCI mo	UCI mo	slow $t_{1/2}$ mo.	LCI mo	UCI mo	R2	2 phase by shape or NLRA
Krop[341]	breast HER2+	y	404	T-DM1	HER2 mono	5.9	5.8	6.1	0.98	1 phase	n											n
Verma[137]	breast HER2+	y	495	T-DM1	HER2 mono	9.6	9.4	9.9	0.98	S	n											n
Inoue[342]	breast HER2+	n	56	trastuzumab	HER2 mono	4.2	4	4.4	0.98	2 phase	n											y
Vogel[343]	breast HER2 F+	n	79	trastuzumab	HER2 mono	5.5	5.1	5.8	0.95	2 phase	y	79.5	64.5	?	3.4	2.9	?	3.80E+15	?	?	0.98	y
Vogel[343]	breast HER2+	n	111	trastuzumab	HER2 mono	4.1	3.8	4.4	0.92	2 phase	y	82.3	65.1	85.7	2.8	2.3	?	5.50E+15	?	?	0.97	y
Creagan[344]	renal	y/n	87	interferon	Interferon	2.7	2.6	2.8	0.98	2 phase	y	89.5	85.3	94.4	2.2	2	2.4	2.20E+12	4	?	0.98	y
Escudier[345]	renal	n	92	interferon	Interferon	6.4	6.2	6.6	0.95	2 phase	y	94	87.6	?	5.8	5.1	6.6	4.30E+09	6.2	?	0.96	y
Escudier[205]	renal	n	322	interferon	Interferon	5.6	5.4	5.8	0.98	1 phase	y	98.8	94.9	?	5.4	5	5.8	5.70E+10	?	?	0.98	y
Gore[346]	renal	n	502	interferon	Interferon	6.2	5.9	6.4	0.97	2 phase	y	76.8	62.3	87.8	4.2	3.6	4.8	20.3	13.6	47.2	0.99	y
Hudes[347]	renal	n	207	interferon	Interferon	2.2	2.1	2.4	0.96	2 phase	y	68.9	45.6	88.5	1.4	1	1.7	6.4	4.1	27.3	0.98	y
Motzer[348]	renal	n	145	interferon	Interferon	5.5	5.3	5.7	0.99	2 phase	y	89.5	70.8	93	4.4	3.7	?	81.8	13.8	?	0.99	y
Motzer[349]	renal	n	375	interferon	Interferon	5.1	5	5.3	0.97	2 phase	y	83.2	76.9	89.5	3.7	3.3	4.3	2.20E+12	71.8	infin	0.98	y
Rini[350]	renal	n	363	interferon	Interferon	5.3	5.1	5.6	0.97	2 phase	y	94	92	96.1	4.6	4.3	4.9	7.30E+11	11	?	0.98	y
Bang[351]	gastric/GEJ	y	57	Ipilimumab	Ipilimumab	3.2	3.1	3.4	0.93	Con-low	n											n
Hodi[121]	melanoma	y	137	ipilimumab	Ipilimumab	3.2	2.9	3.5	0.89	2 phase	y	92.1	67.5	100	2.7	2.1	4	5.90E+15	?	?	0.92	y
Larkin[215]	melanoma	n	315	Ipilimumab	Ipilimumab	4.3	4	4.6	0.9	2 phase	y	93.2	?	?	3.7	?	4.4	4.60E+15	?	?	0.9	y
Robert[352]	melanoma	y/n	278	Ipilimumab	Ipilimumab	3.3	3.2	3.5	0.93	S	n											n
Wolchok[219]	melanoma	n	315	Ipilimumab	Ipilimumab	4.7	4.2	5.3	0.84	2 phase	y	87.2	80.9	89.3	3.3	2.9	?	3.80E+15	?	?	0.94	y
Chiarion[353]	melanoma $\leq$ 70	y	622	Ipilimumab	Ipilimumab	4.7	4.4	4.9	0.96	2 phase	y	90.3	83.7	91.6	3.6	3.3	?	6.00E+15	?	?	0.98	y
Chiarion[353]	melanoma >70	y	193	Ipilimumab	Ipilimumab	5.1	4.8	5.3	0.97	2 phase	y	92.7	88.4	97.8	4.4	3.9	4.9	1.00E+11	6.3	?	0.97	y
Larkin[215]	melanoma-PDL1-	n	162	Ipilimumab	Ipilimumab	3.6	3.3	3.9	0.9	2 phase	n											y
Larkin[215]	melanoma-PDL1+	n	144	Ipilimumab	Ipilimumab	4.7	4.4	5.2	0.89	2 phase	n											y
Zimmer[354]	melanoma-uveal	y/n	53	Ipilimumab	Ipilimumab	3.5	3	4	0.71	Con-high	n											n
Beer[355]	prostate	y	400	ipilimumab	Ipilimumab	6.8	6.6	7	0.98	2 phase	y	93.6	81.9	100	5.8	5.1	6.7	94	12	infin	0.98	y
Kwon[356]	prostate	y	399	Ipilimumab	Ipilimumab	4.3	4.1	4.5	0.97	2 phase	n											y
Doi[357]	gastric	y	53	everolimus	mTOR inh	2.5	2.3	2.6	0.92	Con-low	n											n
Ohtsu[358]	gastric	y	439	everolimus	mTOR inh	2.1	2	2.2	0.95	S	n											n
Yao[359]	GI neuroend	y	207	everolimus	mTOR inh	12.1	11.8	12.4	0.98	S	n											n
Buzzoni[360]	GI/lung neuroend-no prior chemo	n	151	everolimus	mTOR inh	12.6	12.1	13.1	0.98	1 phase	n											n
Buzzoni[360]	GI/lung neuroend-no prior SSA	n	151	everolimus	mTOR inh	12.2	11.6	12.9	0.97	1 phase	n											n
Buzzoni[360]	GI/lung neuroend-prior chemo	y	54	everolimus	mTOR inh	9	8.5	9.5	0.97	Con-low	n											n
Buzzoni[360]	GI/lung neuroend-prior SSA	y	139	everolimus	mTOR inh	11.9	11.5	12.4	0.98	1 phase	n											n

Continuation of Supplementary Online Table 2. Exponential decay nonlinear regression analysis models for different therapies and references for sources of published PFS curves

author	Tumor type	Prior Rx	no. pts	Rx	class Rx	PFS $t_{1/2}$ mo.	LCI mo	UCI mo	R^2	PFS curve shape	2-phase by NLRA	% fast	LCI %	UCI %	fast $t_{1/2}$ mo.	LCI mo	UCI mo	slow $t_{1/2}$ mo.	LCI mo	UCI mo	R2	2 phase by shape or NLRA
Zhu[361]	hepatocell	y	362	everolimus	mTOR inh	3.3	3.2	3.5	0.95	1 phase	n											n
Ou[362]	mesothelioma	y	58	everolimus	mTOR inh	3.1	2.8	3.5	0.81	Con-mod	n											n
Kulke[363]	pancreas neuroend	y/n	81	everolimus	mTOR inh	16.9	16.2	17.7	0.94	Con-low	n											n
Choueiri[364]	renal	y	188	everolimus	mTOR inh	4.4	4.2	4.6	0.96	1 phase	n											n
Escudier[365]	renal	n	92	everolimus	mTOR inh	4.7	4.4	5.1	0.92	Con-low	n											n
Hudes[347]	renal	n	209	temsirolimus	mTOR inh	4.2	4.1	4.4	0.98	1 phase	n											n
Hutson[366]	renal	y	259	temsirolimus	mTOR inh	4.3	4.2	4.5	0.98	Con-low	n											n
Motzer[367]	renal	n	238	everolimus	mTOR inh	7.6	7.4	7.8	0.99	2 phase	y	93	76.2	95.5	6.5	5.8	?	3.40E+15	?	?	0.99	y
Motzer[368]	renal	y	397	everolimus	mTOR inh	4.8	4.6	5	0.98	2 phase	y	88.3	34.3	100	4	2.9	6.8	17.9	3.1	infin	0.99	y
Motzer[45]	renal	y	50	everolimus	mTOR inh	5	4.8	5.2	0.97	S	n											n
Demetri[369]	sarcoma	Mnt	343	ridaforolimus	mTOR inh	4.4	4.2	4.6	0.97	1 phase	n											n
Hodi[121]	melanoma	y	136	gp100	Other	2.8	2.4	3.3	0.61	Con-high	n											n
Bellmunt[370]‡	urothelial	y	272	taxane or vinflunine	Other	3.4	3.1	3.6	0.95	1 phase	n											n
Brada[162]	glioma	n	111	temozol-21 day	Other	3.7	3.6	3.8	0.98	Con-mod	n											n
Brada[162]	glioma	n	112	temozol-5 day	Other	4.9	4.8	5	0.99	2 phase	n											y
Demetri[226]	sarcoma	y	345	trabectidin	Other	4.1	4	4.3	0.97	1 phase	n											n
Overman[371]	colon MSI-H	y	74	nivolumab	PD1/PDL1	12.5	11.5	13.7	0.6	2 phase	y	46.7	41.7	52.4	2.2	1.8	2.6	89.5	52.4	525.4	0.97	y
Shitara[372]	gastric/GEJ	y	196	pembrolizumab	PD1/PDL1	9.1	8.8	9.4	0.88	2 phase	y	46.5	25	81.7	4.3	2.7	6.3	16.5	12.4	97.1	0.99	y
Bauml[373]	head/neck	y	171	pembrolizumab	PD1/PDL1	2.9	2.6	3.1	0.86	S	n											n
Chow[374]	head/neck	y	132	pembrolizumab	PD1/PDL1	2.9	2.6	3.1	0.91	2 phase	y	88.9	28.2	100	2.2	1.4	6.5	4.80E+15	?	?	0.93	y
Ferris[375]	head/neck	y	240	nivolumab	PD1/PDL1	2.5	2.3	2.7	0.92	2 phase	y	94.7	83.47	100	2.3	?	?	9.40E+11	5.1	infin	0.92	y
Bauml[373]	head/neck HPV-	y	131	pembrolizumab	PD1/PDL1	2.6	2.4	2.8	0.89	S	n											n
Younes[376]	Hodgkin's	y	80	nivolumab	PD1/PDL1	15.1	14.1	16.3	0.9	Con-low	n											n
Tumeh[377]	mel/NSCLC- no liver	y	151	pembrolizumab	PD1/PDL1	16.8	15.5	18.2	0.81	2 phase	y	30.7	24.5	39.7	2.6	2	3.5	44.5	33.6	81.9	0.98	y
Tumeh[377]	mel/NSCLC-liver	y	72	pembrolizumab	PD1/PDL1	5.9	5.4	6.5	0.91	2 phase	y	76.9	61.9	81.4	3.3	2.7	3.9	3.20E+15	?	?	0.97	y
Larkin[215]	melanoma	n	316	nivolumab	PD1/PDL1	8.3	7.8	8.8	0.85	2 phase	y	64.2	45.9	68.5	3.5	2.6	?	3.20E+15	?	?	0.94	y
Larkin[215]	melanoma PDL1-	n	165	nivolumab	PD1/PDL1	6.5	6.1	6.9	0.84	2 phase	y	69.2	27.6	100	3	1.9	7.2	3.90E+15	?	?	0.93	y
Larkin[215]	melanoma PDL1+	n	143	nivolumab	PD1/PDL1	12.7	12.2	13.3	0.92	2 phase	y	35.4	0	100	3.6	1.5	infin	36.3	8.3	infin	0.95	y
Ribas[378]	melanoma	y	180	pembro 2 mg/kg	PD1/PDL1	4.2	3.9	4.6	0.91	S	n											n
Ribas[378]	melanoma	y	181	pembro 10 mg/kg	PD1/PDL1	4.8	4.5	5.2	0.88	2 phase	y	88	0	100	3.9	1.3	infin	6.40E+08	0.6	infin	0.88	y
Ribas[379]	melanoma	n	152	pembrolizumab	PD1/PDL1	12.5	12	13.1	0.91	2 phase	y	28.3	17.6	39	2.5	1.8	4.5	21.1	17.1	27.6	0.97	y
Ribas[379]	melanoma	y/n	655	pembrolizumab	PD1/PDL1	7.9	7.4	8.6	0.78	2 phase	y	64.2	52.6	75.7	2.9	2.3	3.7	46.3	22.9	infin	0.95	y
Robert[380]	melanoma	y	89	pembro 2 mg/kg	PD1/PDL1	5.3	5	5.7	0.9	2 phase	y	92.6	0	100	4.7	2.5	52.8	4.10E+10	0.8	infin	0.9	y
Robert[380]	melanoma	y	84	pembro 10 mg/kg	PD1/PDL1	4.4	4.1	4.7	0.9	S	n											n
Robert[228]	melanoma B-WT	n	210	nivolumab	PD1/PDL1	7	6.5	7.6	0.77	2 phase	y	61.5	14	100	2.7	1.6	9.7	5.00E+15	?	?	0.89	y
Robert[352]	melanoma	y	279	pembro q2wk	PD1/PDL1	5.7	5.4	6	0.91	2 phase	y	88.2	61.9	100	4.8	?	?	2.60E+11	7.9	infin	0.91	y
Robert[352]	melanoma	y	277	pembro q3wk	PD1/PDL1	5.5	5.2	5.8	0.88	2 phase	y	87.8	67.8	100	4.6	?	?	1.70E+12	15.4	infin	0.88	y
Topalian[381]	melanoma	y	107	nivolumab	PD1/PDL1	7.3	6.6	8.1	0.71	2 phase	y	64.9	50.4	79.4	2.6	2	3.6	58.4	20.8	infin	0.92	y

Continuation of Supplementary Online Table 2. Exponential decay nonlinear regression analysis models for different therapies and references for sources of published PFS curves

author	Tumor type	Prior Rx	no. pts	Rx	class Rx	PFS $t_{1/2}$ mo.	LCI mo	UCI mo	R^2	PFS curve shape	2-phase by NLRA	% fast	LCI %	UCI %	fast $t_{1/2}$ mo.	LCI mo	UCI mo	slow $t_{1/2}$ mo.	LCI mo	UCI mo	R2	2 phase by shape or NLRA
Wolchok[219]	melanoma	n	316	nivolumab	PD1/PDL1	14.6	13	16.3	0.53	2 phase	y	57.9	50.9	65.5	3.5	2.9	4.2	101	55.8	1615	0.93	y
Algazi[382]	melanoma uveal	y/n	56	nivol or pembro or atezo	PD1/PDL1	2.7	2.5	2.9	0.93	2 phase	n											y
Weber[383]	melanoma-C4-6	y	92	nivolumab	PD1/PDL1	8.2	7.3	9.2	0.8	2 phase	y	70.9	58.5	?	3.7	3.1	?	5.70E+15	?	?	0.93	y
Kaufman[384]	Merkel	y	88	avelumab	PD1/PDL1	3.8	3.4	4.1	0.74	2 phase	y	69.2	57.7	72.5	1.6	1.3	1.8	4.80E+15	?	?	0.91	y
Fehrenbacher[385]	NSCLC	y	144	atezolizumab	PD1/PDL1	3.8	3.8	4.1	0.93	2 phase	y	57.7	26.8	88.5	1.7	1.2	3.3	9.5	5.2	52.7	0.98	y
Borghaei[386]	NSCLC nonsqu	y	292	nivolumab	PD1/PDL1	3.8	3.4	4.2	0.92	2 phase	y	86.5	83.2	90	2.5	?	?	4.00E+12	44.2	infin	0.98	y
Borghaei[386]	NSCLC PDL1<1%	y	108	nivolumab	PD1/PDL1	2.7	2.4	3.1	0.95	2 phase	y	89.2	45.5	100	2.1	1.4	4.6	3.30E+15	?	?	0.92	y
Borghaei[386]	NSCLC PDL1<5%	y	136	nivolumab	PD1/PDL1	2.7	2.5	3	0.89	2 phase	y	91.3	56.1	100	2.2	1.6	3.9	4.10E+15	?	?	0.94	y
Borghaei[386]	NSCLC PDL1<10%	y	145	nivolumab	PD1/PDL1	3	2.7	3.3	0.92	2 phase	y	89	68	100	2.3	1.8	3.1	5.80E+11	5.4	infin	0.95	y
Borghaei[386]	NSCLC PDL1>1%	y	123	nivolumab	PD1/PDL1	5.1	4.7	5.6	0.9	2 phase	y	67.4	52	82.9	2.3	1.9	3.1	33.2	13.1	infin	0.99	y
Borghaei[386]	NSCLC PDL1>5%	y	95	nivolumab	PD1/PDL1	6.5	6.1	7	0.91	2 phase	y	75.5	41.9	100	3.6	2.6	6.3	5.50E+15	?	?	0.98	y
Borghaei[386]	NSCLC PDL1>10%	y	86	nivolumab	PD1/PDL1	6.1	5.7	6.6	0.92	2 phase	y	74.5	54.9	94.1	3.2	2.5	4.4	4.40E+15	?	?	0.99	y
Brahmer[387]	NSCLC squam	y	135	nivolumab	PD1/PDL1	4.6	4.3	4.8	0.96	2 phase	y	87.7	51.6	100	3.4	2.4	5.7	4.20E+15	?	?	0.97	y
Brahmer[387]	NSCLC PDL1<1%	y	54	nivolumab	PD1/PDL1	3.8	3.7	4	0.96	2 phase	y	72.5	0	100	2.7	0.9	infin	10.9	1	infin	0.97	y
Brahmer[387]	NSCLC PDL1<5%	y	75	nivolumab	PD1/PDL1	3.2	3	3.5	0.96	2 phase	y	63.2	0	100	1.8	1.1	7	8.6	2.9	infin	0.98	y
Brahmer[387]	NSCLC PDL1<10%	y	81	nivolumab	PD1/PDL1	3.7	3.5	3.9	0.97	2 phase	y	88.7	57.5	100	2.9	2.2	4.3	1.00E+11	3.5	infin	0.97	y
Brahmer[387]	NSCLC PDL1>1%	y	63	nivolumab	PD1/PDL1	4.4	4	4.8	0.91	2 phase	y	76.1	39.9	100	2.4	1.6	4.6	6.20E+15	?	?	0.97	y
Fehrenbacher[385]	NSCLC TC&IC 0	y	51	atezolizumab	PD1/PDL1	2.8	2.5	3	0.92	2 phase	y	83	22.6	100	1.8	1.1	6.4	3.30E+15	?	?	0.95	y
Fehrenbacher[385]	NSCLC TC-IC 1-3	y	93	atezolizumab	PD1/PDL1	4.2	4	4.6	0.94	2 phase	y	53.4	11.5	95.2	1.9	1.2	5.2	9.4	4.9	124	0.97	y
Fehrenbacher[385]	NSCLC TC-IC 2-3	y	50	atezolizumab	PD1/PDL1	4.4	4	4.8	0.92	2 phase	y	69.2	11.2	100	2.3	1.3	8.9	25.3	4	infin	0.94	y
Garon[388]	NSCLC PDL1<1%	y/n	76	pembrolizumab	PD1/PDL1	3	2.8	3.3	0.92	S	n											n
Garon[388]	NSCLC PDL1 1-49%	y/n	161	pembrolizumab	PD1/PDL1	3.5	3.2	3.8	0.91	1 phase	n											n
Garon[388]	NSCLC PDL1>50%	y/n	119	pembrolizumab	PD1/PDL1	7.1	6.6	7.8	0.8	2 phase	y	57	30.7	83.4	2.4	1.6	4.4	104	13.1	infin	0.96	y
Gettinger[389]	NSCLC	y	129	nivolumab	PD1/PDL1	4.1	3.7	4.6	0.93	2 phase	y	87.7	53	100	3	2	5.5	4.60E+15	?	?	0.93	y
Gettinger[390]	NSCLC	n	52	nivolumab	PD1/PDL1	4.7	4.4	5	0.9	2 phase	y	89.1	0	100	3.9	1.6	infin	3.80E+15	?	?	0.94	y
Gulley[391]	NSCLC PDL1+	y	122	avelumab	PD1/PDL1	3.4	3.3	3.6	0.95	2 phase	y	90.4	16.9	92.2	2.9	?	2.9	4.80E+15	?	?	0.96	y
Herbst[392]	NSCLC	y	344	pembro 2 mg/kg	PD1/PDL1	4.1	4	4.3	0.97	2 phase	y	90.8	62.9	100	3.3	2.6	4.8	3.70E+15	?	?	0.98	y
Herbst[392]	NSCLC	y	346	pembro 10 mg/kg	PD1/PDL1	4.7	4.5	5	0.94	2 phase	y	79.5	55.1	100	3	2.3	4.2	44.7	8.1	infin	0.98	y
Herbst[392]	NSCLC PDL1>50%	y	139	pembro 2 mg/kg	PD1/PDL1	5.9	5.6	6.1	0.96	2 phase	y	79.7	40.6	100	3.7	2.6	6.5	3.30E+15	?	?	0.98	y
Herbst[392]	NSCLC PDL1>50%	y	151	pembro 10 mg/kg	PD1/PDL1	6.5	6.1	6.9	0.89	2 phase	y	72.5	44.3	100	3.3	2.4	5.3	5.70E+15	?	?	0.97	y
Peters[393]	NSCLC TC-IC 2	n	74	atezolizumab	PD1/PDL1	4.7	4.5	4.8	0.96	2 phase	y	11.6	?	?	2	?	?	5.2	4.5	?	0.96	y
Peters[393]	NSCLC TC-IC 2	y	145	atezol-2nd line	PD1/PDL1	3	2.9	3.2	0.93	2 phase	y	90.1	62.6	93.4	2.4	1.8	?	6.10E+15	?	?	0.95	y
Peters[393]	NSCLC TC-IC 2	y	136	atezol-3rd line	PD1/PDL1	2.9	2.8	3.1	0.93	2 phase	y	93.8	89.7	98.5	2.5	2.3	2.9	1.20E+12	?	?	0.94	y
Peters[393]	NSCLC TC-IC 3	n	65	atezolizumab	PD1/PDL1	5.7	5.5	6	0.94	2 phase	y	49.2	14.7	?	2.8	1.3	4.4	12	7	?	0.95	y
Peters[393]	NSCLC TC-IC 3	y	122	atezol-2nd line	PD1/PDL1	4.4	4.2	4.7	0.89	2 phase	y	80	63.1	82.6	2.6	2.1	?	3.50E+15	?	?	0.95	y
Peters[393]	NSCLC TC-IC 3	y	115	atezol-3rd line	PD1/PDL1	4.5	4.4	4.7	0.96	2 phase	y	90.9	86.8	95.7	3.7	3.4	4.2	9.00E+11	5.6	?	0.96	y
Reck[196]	NSCLC PDL1+	n	154	pembrolizumab	PD1/PDL1	9.4	9	9.9	0.88	2 phase	y	47.7	24.3	71.2	3	2.1	5.2	50.8	16.2	infin	0.98	y
Rizvi[394]	NSCLC squam	y	117	nivolumab	PD1/PDL1	3.3	3.1	3.6	0.85	2 phase	y	81.7	48.6	100	2	1.4	3.6	4.80E+15	?	?	0.92	y
Motzer[368]	renal	y	406	nivolumab	PD1/PDL1	5.4	5.1	5.8	0.93	2 phase	y	86.5	70	100	3.8	3.1	4.8	613.7	12.4	infin	0.97	y
Motzer[395]	renal	y	60	nivol 0.3 mg/kg	PD1/PDL1	3.6	3.3	4	0.8	2 phase	y	78.5	66	91	1.9	1.6	2.4	4.10E+15	?	?	0.96	y
Motzer[395]	renal	y	54	nivol 2 mg/kg	PD1/PDL1	3.9	3.7	4.1	0.95	1 phase	y	94.8	0	100	3.5	1.4	infin	9.20E+09	0.3	infin	0.95	y
Motzer[395]	renal	y	54	nivol 10 mg/kg	PD1/PDL1	4.2	4.1	4.4	0.98	1 phase	n											n

Continuation of Supplementary Online Table 2. Exponential decay nonlinear regression analysis models for different therapies and references for sources of published PFS curves

author	Tumor type	Prior Rx	no. pts	Rx	class Rx	PFS $t_{1/2}$ mo.	LCI mo	UCI mo	R^2	PFS curve shape	2-phase by NLRA	% fast	LCI %	UCI %	fast $t_{1/2}$ mo.	LCI mo	UCI mo	slow $t_{1/2}$ mo.	LCI mo	UCI mo	R2	2 phase by shape or NLRA
Antonia[396]	SCLC	y	98	nivolumab	PD1/PDL1	2	1.5	3.4	0.89	2 phase	n											y
Le[397]	tumors MSI-H	y	86	PD1/PDL1	PD1/PDL1	19.3	17.7	21.1	0.73	2 phase	y	29.5	23.1	39.4	2.5	1.8	3.7	52.6	38.2	118.4	0.97	y
Bellmunt[370]	urothelial	y	270	pembrolizumab	PD1/PDL1	3.2	2.9	3.6	0.85	2 phase	y	82.5	59.6	100	2	1.5	3	1.10E+11	6.8	infin	0.93	y
Powles[398]	urothelial	y	191	durvalumab	PD1/PDL1	2.3	2.1	2.6	0.86	2 phase	y	87.2	81.9	93.1	1.7	1.5	2.1	1.00E+11	6	?	0.89	y
Powles[398]	urothelial high PDL1	y	98	durvalumab	PD1/PDL1	3.1	2.8	3.4	0.87	2 phase	y	79.8	53.7	85.6	1.9	1.4	2.2	3.40E+15	?	?	0.92	y
Powles[398]	urothelial low PDL1	y	79	durvalumab	PD1/PDL1	1.7	1.4	2	0.62	Con-high	n											n
Sharma[399]	urothelial	y	86	nivolumab	PD1/PDL1	3.9	3.5	4.4	0.85	2 phase	y	51.4	16.5	86.3	1.4	0.83	4.2	11	5	infin	0.94	y
Park[130]	breast	Mnt	115	BSC	placebo/BSC	4.4	4.2	4.6	0.98	2 phase	y	90	71	100	3.5	2.9	4.4	6.10E+15	?	?	0.99	y
Van Cutsem[232]	colon	y	232	BSC	placebo/BSC	1.5	1.3	1.7	0.84	Con-mod	n											n
Van Cutsem[233]	colon	y	176	BSC	placebo/BSC	1.3	1.2	1.5	0.87	Con-high	n											n
Jonker[235]	colon EG IHC+	y	285	BSC	placebo/BSC	2	1.8	2.3	0.82	Con-high	n											n
Amado[236]	colon KRAS WT	y	119	BSC	placebo/BSC	1.5	1.3	1.6	0.85	Con-mod	n											n
Karapetis[237]	colon KRAS WT	y	105	BSC	placebo/BSC	2.1	1.9	2.3	0.79	Con-high	n											n
Amado[236]	colon KRAS-m	y	100	BSC	placebo/BSC	1.5	1.4	1.7	0.88	Con-mod	n											n
Bang[351]¶	gastric/GEJ	Mnt	57	BSC	placebo/BSC	4.9	4.8	5	0.97	1 phase	n											n
Machiels[239]	head/neck	y	95	BSC or MTX	placebo/BSC	2.2	2	2.3	0.86	Con-mod	n											n
Jassem[83]	mesothelioma	y	120	BSC	placebo/BSC	2.1	1.9	2.4	0.94	2 phase	y	91.5	76.3	94.8	1.7	1.4	1.9	3.60E+12	8.1	?	0.96	y
Brodowicz[324]	NSCLC	Mnt	68	BSC	placebo/BSC	2.4	2.2	2.5	0.94	S	n											n
Perol[279]	NSCLC	y	155	BSC	placebo/BSC	2.1	1.9	2.2	0.94	2 phase	n											y
Ranson[400]	NSCLC	n	78	BSC	placebo/BSC	1.3	1.1	1.4	0.83	Con-mod	n											n
Paz-Ares[89]	NSCLC nonsqu	Mnt	180	BSC	placebo/BSC	2.8	2.6	2.9	0.97	1 phase	n											n
Grothey[401]	colon	y	255	placebo	placebo/BSC	1.5	1.3	1.7	0.75	Con-mod	n											n
Mayer[306]	colon	y	266	placebo	placebo/BSC	1.5	1.3	1.9	0.75	S	n											n
Rao[402]	colon	y	133	placebo	placebo/BSC	2.4	2.1	2.7	0.71	Con-high	n											n
Li[42]	gastric	y	91	placebo	placebo/BSC	1.6	1.3	1.8	0.74	Con-low	n											n
Ohtsu[358]	gastric	y	217	placebo	placebo/BSC	1.5	1.3	1.6	0.87	S	n											n
Pavlakis[403]	gastric	y	50	placebo	placebo/BSC	1	0.9	1.2	0.71	Con-high	n											n
Caplin[404]	GI neuroend	n	103	placebo	placebo/BSC	18.9	17.8	20	0.90	1 phase	n											n
Yao[405]	GI neuroend	y	203	placebo	placebo/BSC	4.9	4.6	5.2	0.94	S	n											n
Demetri[337]	GIST	y	105	placebo	placebo/BSC	1.8	1.6	2	0.78	mod convex	n											n
Demetri[336]	GIST	y	66	placebo	placebo/BSC	1.2	1	1.3	0.77	Con-mod	n											n
Abou-Alfa[406]	hepatocell	y	211	placebo	placebo/BSC	3.2	2.8	3.5	0.84	S	n											n
Llovet[407]	hepatocell	n	303	placebo	placebo/BSC	3.3	3.1	3.5	0.92	S	n											n
Llovet[43]	hepatocell	y	132	placebo	placebo/BSC	2.6	2.3	3	0.85	S	n											n
Zhu[408]	hepatocell	y	282	placebo	placebo/BSC	2.4	2.2	2.6	0.92	1 phase	n											n
Eisen[409]	melanoma	y	154	placebo	placebo/BSC	2.6	2.5	2.8	0.92	S	n											n
Cappuzzo[252]	NSCLC	Mnt	451	placebo	placebo/BSC	2.5	2.3	2.6	0.95	1 phase	n											n
Cicenas[255]	NSCLC	Mnt	322	placebo	placebo/BSC	3.1	2.9	3.3	0.96	2 phase	n											y
Ciuleanu[76]	NSCLC	Mnt	222	placebo	placebo/BSC	2.4	2.2	2.6	0.93	Con-mod	n											n
Ellis[258]	NSCLC	y	240	placebo	placebo/BSC	2.4	2.2	2.6	0.93	2 phase	y	87.5	16	100	1.8	1	7.8	4.40E+15	?	?	0.94	y
Fidias[410]	NSCLC	y	98	delayed docet	placebo/BSC	2.8	2.6	2.9	0.97	2 phase	y	95.9	89.2	98.3	2.5	2.3	?	4.20E+15	?	?	0.98	y
Gaafer[260]	NSCLC	Mnt	87	placebo	placebo/BSC	2.7	2.5	2.8	0.96	2 phase	n											y

Continuation of Supplementary Online Table 2. Exponential decay nonlinear regression analysis models for different therapies and references for sources of published PFS curves																						
author	Tumor type	Prior Rx	no. pts	Rx	class Rx	PFS $t_{1/2}$ mo.	LCI mo	UCI mo	R^2	PFS curve shape	2-phase by NLRA	% fast	LCI %	UCI %	fast $t_{1/2}$ mo.	LCI mo	UCI mo	slow $t_{1/2}$ mo.	LCI mo	UCI mo	R2	2 phase by shape or NLRA
Goss[262]	NSCLC	n	101	placebo	placebo/BSC	1.4	1.3	1.4	0.94	2 phase	y	95.7	58.5	100	1.2	0.9	2	3.50E+15	?	?	0.94	y
Johnson[411]	NSCLC	Mnt	92	placebo	placebo/BSC	3	2.9	3.2	0.96	2 phase	y	95.5	85.2	100	2.7	2.4	3.2	5.50E+15	?	?	0.97	y
Lee[271]	NSCLC	y	307	placebo	placebo/BSC	1.8	1.6	2.1	0.85	S	n											n
Lee[272]	NSCLC	n	320	placebo	placebo/BSC	2.5	2.4	2.6	0.99	2 phase	n											y
Miller[412]	NSCLC	y	195	placebo	placebo/BSC	1.5	1.3	1.6	0.84	S	n											n
O'Brien[413]	NSCLC	Mnt	52	placebo	placebo/BSC	3.3	3.1	3.4	0.97	1 phase	n											n
Parikh[414]	NSCLC	y	50	placebo	placebo/BSC	1.5	1.3	1.7	0.75	Con-high	n											n
Paz-Ares[415]	NSCLC	y	353	placebo	placebo/BSC	1.9	1.8	2.1	0.89	2 phase	n											y
Ramalingam[416]	NSCLC	y	242	placebo	placebo/BSC	2.2	2	2.3	0.94	S	n											n
Shepherd[288]	NSCLC	y	731	placebo	placebo/BSC	1.8	1.7	2	0.93	1 phase	n											n
Thatcher[293]	NSCLC	y	563	placebo	placebo/BSC	2.8	2.7	2.9	0.98	S	n											n
Zhang[299]	NSCLC	Mnt	148	placebo	placebo/BSC	2.4	2.2	2.5	0.94	1 phase	n											n
Cappuzzo[252]	NSCLC EGFR WT	Mnt	163	placebo	placebo/BSC	2.2	2	2.4	0.89	2 phase	n											y
Ledermann[417]	ovary	Mnt	129	placebo	placebo/BSC	4.7	4.4	5.2	0.87	Con-mod	n											n
Raymond[418]	pancreas neuroend	y	85	placebo	placebo/BSC	5.4	5.1	5.8	0.97	Con-low	n											n
Beer[71]	prostate	y	845	placebo	placebo/BSC	4.9	4.6	5.2	0.92	1 phase	n											n
Beer[355]	prostate	y	202	placebo	placebo/BSC	4.1	3.9	4.5	0.92	2 phase	y	95.8	73.4	100	3.7	2.9	5.3	3.80E+15	?	?	0.92	y
de Bono[69]	prostate	y	398	placebo	placebo/BSC	4.5	4.2	4.8	0.92	Con-low	n											n
Kwon[356]	prostate	y	400	placebo	placebo/BSC	3.3	3.1	3.6	0.92	S	n											n
Scher[72]	prostate	y	399	placebo	placebo/BSC	4.1	3.7	4.5	0.85	Con-low	n											n
Sternberg[419]	prostate	y	413	placebo	placebo/BSC	4.9	4.6	5.3	0.89	S	n											n
Kang[41]	renal	y	68	placebo	placebo/BSC	2.2	2	2.5	0.79	Con-mod	n											n
Motzer[420]	renal	y	139	placebo	placebo/BSC	2.2	2	2.5	0.78	Con-high	n											n
Sternberg[47]	renal	y/n	145	placebo	placebo/BSC	4	3.8	4.2	0.94	1 phase	y	91.5	84.9	?	3.4	2.9	4	6.20E+10	4	?	0.95	y
Escudier[421]	renal	y	452	placebo	placebo/BSC	2.8	2.7	2.9	0.92	Con-low	n											n
Escudier[422]	renal-high VEGF	y	172	placebo	placebo/BSC	3.1	3	3.3	0.94	1 phase	n											n
Escudier[422]	renal-low VEGF	y	176	placebo	placebo/BSC	3.8	3.7	4	0.96	2 phase	y	95.8	64.3	100	3.5	?	?	3.40E+10	1.5	infin	0.96	y
Demetri[369]	sarcoma	Mnt	359	placebo	placebo/BSC	3.3	3.1	3.5	0.96	2 phase	y	94.3	73.4	100	2.8	2.3	3.8	5.10E+15	?	?	0.92	y
Schlumberger[46]	thyroid	y	131	placebo	placebo/BSC	3.5	3.3	3.7	0.94	S	n											n
Banerjee[423]	ovary	n	481	carbo-flat dosing	Plat 1st Rx ovary	14.4	13.8	15	0.95	2 phase	y	89.5	80	93.7	11.6	10.5	12.8	5.10E+15	?	?	0.96	y
Banerjee[423]	ovary	n	483	carbo-dose escalation	Plat 1st Rx ovary	14.6	13.9	15.3	0.94	2 phase	y	88.3	81.4	91.6	11.3	10.3	12.3	4.60E+15	?	?	0.96	y
Gore[424]	ovary	n	110	carbo-AUC12	Plat 1st Rx ovary	14	13.4	14.7	0.95	2 phase	y	84.1	79	86.8	9.7	8.9	10.4	4.30E+15	?	?	0.97	y
Gore[424]	ovary	n	117	carbo-AUC6	Plat 1st Rx ovary	15	14.3	15.6	0.95	2 phase	y	88.2	79.5	91.8	11.5	10.5	12.7	3.30E+15	?	?	0.96	y
ICONG[425]	ovary	n	943	carboplatin	Plat 1st Rx ovary	18	17.4	18.5	0.95	2 phase	y	85.9	80.9	87.6	13.1	12.4	?	6.00E+15	?	?	0.99	y
Lambert[426]	ovary	n	115	Plat 8 cycles	Plat 1st Rx ovary	14.9	14.4	15.5	0.98	S	n											n
Lambert[426]	ovary	n	116	cisplatin	Plat 1st Rx ovary	13.1	12.6	14	0.97	2 phase	n											y
Lambert[426]	ovary	n	117	carboplatin	Plat 1st Rx ovary	15.2	14.7	15.8	0.97	2 phase	n											y
Lambert[426]	ovary	n	118	Plat 5 cycles	Plat 1st Rx ovary	12.4	11.9	13	0.96	S	n											n

Continuation of Supplementary Online Table 2. Exponential decay nonlinear regression analysis models for different therapies and references for sources of published PFS curves

author	Tumor type	Prior Rx	no. pts	Rx	class Rx	PFS $t_{1/2}$ mo.	LCI mo	UCI mo	R^2	PFS curve shape	2-phase by NLRA	% fast	LCI %	UCI %	fast $t_{1/2}$ mo.	LCI mo	UCI mo	slow $t_{1/2}$ mo.	LCI mo	UCI mo	R2	2 phase by shape or NLRA
Muggia[427]	ovary	n	200	cisplatin	Plat 1st Rx ovary	16.1	15.8	16.3	0.99	2 phase	n											y
Perren[225]	ovary	n	753	carboplatin	Plat 1st Rx ovary	19.3	18.4	20.3	0.94	S	n											n
Rankin[428]	ovary	n	81	carboplatin	Plat 1st Rx ovary	11.1	10.7	11.5	0.96	1 phase	n											n
Skarlos[429]	ovary	n	73	carboplatin	Plat 1st Rx ovary	17.9	16.9	19.1	0.92	S	n											n
Horwich[430]	seminoma	n	64	carboplatin	Plat 1st Rx ovary	88.1	79.7	98.1	0.006	2 phase	y	29.7	27.2	30.5	7.2	6.2	?	3.80E+15	?	?	0.95	y
Baselga[431]	breast triple-	y/n	58	cisplatin	Plat: other	2.2	1.9	2.6	0.84	Con-mod	n											n
Long[432]	cervix	y/n	146	cisplatin	Plat: other	3.6	3.4	3.8	0.95	1 phase	n											n
Moore[433]	cervix	y/n	134	cisplatin	Plat: other	3.2	3	3.3	0.97	2 phase	n											y
Omura[434]	cervix	n	140	cisplatin	Plat: other	3.2	3.1	3.4	0.96	1 phase	n											n
Burtness[435]	head/neck	n	56	cisplatin	Plat: other	2.7	2.5	2.8	0.97	1 phase	n											n
Patil[436]	head/neck	n	53	cisplatin	Plat: other	2.2	2	2.5	0.91	Con-mod	n											n
Urba[437]	head/neck	n	397	cisplatin	Plat: other	3.1	3	3.2	0.98	S	n											n
Urba[438]	head/neck-A	n	56	cisplatin	Plat: other	2.1	1.9	2.4	0.89	S	n											n
Gatzemeier[439]	NSCLC	n	207	cisplatin	Plat: other	2.7	2.6	2.9	0.97	Con-low	n											n
Sandler[440]	NSCLC	n	261	cisplatin	Plat: other	3.8	3.7	3.9	0.99	Con-low	n											n
von Pawel[441]	NSCLC	n	219	cisplatin	Plat: other	2.4	2.3	2.5	0.99	Con-low	n											n
Wozniak[442]	NSCLC	n	207	cisplatin	Plat: other	2.4	2.2	2.5	0.98	2 phase	y	97.2	74.2	100	2.2	1.7	3.1	3.20E+10	1.1	infin	0.98	y
Bolis[443]	ovary	y	95	carboplatin	Plat: other	16.5	15.7	17.2	0.87	Con-mod	n											n
Pfisterer[444]	ovary	y	178	carboplatin	Plat: other	5.6	5.3	5.9	0.96	Con-low	n											n
Loehrer[445]	urothelial	n	120	cisplatin	Plat: other	2.8	2.6	3	0.92	Con-mod	n											n
Grothey[401]	colon	y	505	regorafenib	RAF non-GIST	2.4	2.3	2.5	0.93	Con-low	n											n
Pavlakis[403]	gastric	y	97	regorafenib	RAF non-GIST	2.4	2.3	2.5	0.97	1 phase	n											n
Abou-Alfa[446]	hepatocell	n	137	sorafenib	RAF non-GIST	4.6	4.4	4.9	0.96	1 phase	n											n
Cainap[40]	hepatocell	n	521	sorafenib	RAF non-GIST	4	3.8	4.2	0.96	2 phase	y	91.9	79.7	100	3.3	2.8	4	3.80E+11	7.9	infin	0.98	y
Cheng[447]	hepatocell	y	544	sorafenib	RAF non-GIST	3.8	3.5	4	0.96	2 phase	y	95.5	70.5	100	3.4	2.6	4.8	3.10E+15	?	?	0.96	y
Llovet[407]	hepatocell	n	299	sorafenib	RAF non-GIST	5.6	5.4	5.7	0.97	S	n											n
Dingemans[448]	NSCLC	y	57	sorafenib	RAF non-GIST	2.5	2.4	2.7	0.95	Con-mod	n											n
Paz-Ares[415]	NSCLC	y	350	sorafenib	RAF non-GIST	3	2.8	3.2	0.93	Con-mod	n											n
Zhou[449]	NSCLC	y	64	sorafenib	RAF non-GIST	3.2	2.9	3.4	0.92	Con-low	n											n
Escudier[345]	renal	n	97	sorafenib	RAF non-GIST	5.9	5.6	6.3	0.88	Con-mod	n											n
Hutson[366]	renal	y	253	sorafenib	RAF non-GIST	3.6	3.5	3.8	0.97	2 phase	n											y
Motzer[44]	renal	y/n	257	sorafenib	RAF non-GIST	9.6	9.2	10.1	0.95	S	n											n
Procopio[450]	renal	n	62	sorafenib	RAF non-GIST	9.1	8.6	9.6	0.92	Con-low	n											n
Escudier[421]	renal	y	451	sorafenib	RAF non-GIST	5.3	4.9	5.8	0.87	Con-high	n											n
Maki[451]	sarcoma	y/n	122	sorafenib	RAF non-GIST	3.4	3.2	3.7	0.91	Con-mod	n											n
Choueiri[364]	renal	y	187	cabozantinib	small mol	7.4	7.1	7.7	0.96	1 phase	n											n
Smith[452]	prostate	y	682	cabozantinib	small mol	5.3	5	5.8	0.91	Con-low	n											n
Rao[402]	colon	y	235	R115777	small mol	2.5	2.3	2.8	0.89	Con-mod	n											n
Caplin[404]	GI neuroend	n	101	lanreotide	small mol	34.9	34.1	35.8	0.97	1 phase	y	60.7	47.3	74.1	18.6	14.8	24.9	1.20E+13	171	infin	0.98	y
Blackwell[453]	breast HER2+	y	78	lapatinib	small mol	3.3	3	3.5	0.89	Con-mod	n											n

Continuation of Supplementary Online Table 2. Exponential decay nonlinear regression analysis models for different therapies and references for sources of published PFS curves																						
author	Tumor type	Prior Rx	no. pts	Rx	class Rx	PFS $t_{1/2}$ mo.	LCI mo	UCI mo	R^2	PFS curve shape	2-phase by NLRA	% fast	LCI %	UCI %	fast $t_{1/2}$ mo.	LCI mo	UCI mo	slow $t_{1/2}$ mo.	LCI mo	UCI mo	R2	2 phase by shape or NLRA
Kaufman[454]	breast HER2+	y	126	lapatinib	small mol	3.3	3.1	3.5	0.95	Con-mod	n											n
Blackwell[106]	breast HER2+	y	148	lapatinib	small mol	1.9	1.8	2	0.95	S	n											n
Fassnacht[455]	adrenal	y	90	lisitinib	small mol	2.2	1.9	2.5	0.72	Con-high	n											n
Ledermann[417]	ovary	y	136	olaparib	small mol	10.4	9.5	11.4	0.89	S	n											n
Fizazi[73]	prostate	y	363	prednisone	small mol	6.1	5.9	6.4	0.97	1 phase	n											n
Michaelson[456]	prostate	y	289	prednisone	small mol	4.6	4.3	4.8	0.97	2 phase	y	97.1	8.9	100	4.3	2.5	16.6	3.80E+15	?	?	0.97	y
Ryan[68]	prostate	y	542	prednisone	small mol	9	8.7	9.2	0.98	1 phase	y	91.2	86.8	95.6	7.5	6.9	8.2	8.00E+12	87.8	infin	0.99	y
Smith[452]	prostate	y	346	prednisone	small mol	3.4	3	3.9	0.79	S	n											n
Sternberg[419]	prostate	y	832	tasquinimod	small mol	6.9	6.6	7.2	0.97	1 phase	n											n
Flaherty[227]	melanoma B-m	n	214	trametinib	small mol	4.7	4.4	521	0.89	Con-mod	n											n
Burstein[457]	breast	y	64	sunitinib	Sunit non-GIST	2.9	2.6	3.2	0.84	Con-mod	n											n
Cheng[447]	hepatocell	y	530	sunitinib	Sunit non-GIST	3.4	3.2	3.6	0.95	Con-low	n											n
Raymond[418]	pancreas neuroend	y	86	sunitinib	Sunit non-GIST	11.8	11.4	12.3	0.97	1 phase	y	73.1	?	?	7.6	?	?	1.40E+10	?	?	0.98	y
Michaelson[456]	prostate	y	584	sunitinib	Sunit non-GIST	6.1	5.9	6.4	0.97	S	n											n
Ansari[458]	renal	y/n	56	sunitinib	Sunit non-GIST	12.7	12.4	13.1	0.97	1 phase	n											n
Motzer[367]	renal	n	233	sunitinib	Sunit non-GIST	10.5	10.2	10.8	0.98	1 phase	n											n
Motzer[220]	renal	n	422	sunitinib	Sunit non-GIST	10.3	9.8	10.8	0.96	2 phase	y	78.1	50.6	81.2	6.3	4.6	6.9	5.50E+15	?	?	0.99	y
Motzer[349]	renal	n	375	sunitinib	Sunit non-GIST	12.5	12	13	0.93	Con-mod	n											n
Buzdar[459]	breast ER+	n	673	tamoxifen	tamoxifen	8.3	8.1	8.6	0.97	2 phase	y	83.7	39.1	88.8	6.1	4.1	6.8	4.40E+15	?	?	0.98	y
Gill[460]	breast ER+	n	58	tamoxifen	tamoxifen	9.5	9	10	0.94	2 phase	y	18.8	14.7	24.2	1.3	0.9	1.8	13.2	12.2	14.7	0.99	y
Howell[312]	breast ER+/?	n	274	tamoxifen	tamoxifen	8	7.8	8.3	0.98	2 phase	n											y
Ingle[461]	breast ER+	n	68	tamoxifen	tamoxifen	15.1	14.4	15.7	0.96	2 phase	y	31.9	26.8	38.6	3.9	3.2	4.8	24.3	22.3	27.3	0.99	y
Klijn[462]	breast ER+	n	50	tamoxifen	tamoxifen	7.1	6.6	7.6	0.94	1 phase	n											n
Lindemann[463]	ovary	y	82	tamoxifen	tamoxifen	2.2	2	2.4	0.89	S	n											n
Mouridsen[17]	breast ER+	n	454	tamoxifen	tamoxifen	7.3	7	7.6	0.96	2 phase	y	89.6	60.8	93.6	5.7	4.5	6.4	1.10E+13	14.2	?	0.96	y
Mouridsen[18]	breast ER+	n	458	tamoxifen	tamoxifen	6.8	6.4	7.1	0.95	2 phase	y	87.6	44.9	93.4	5.2	3.5	5.9	91.5	11.1	?	0.97	y
Mouridsen[19]	breast ER+>70	n	143	tamoxifen	tamoxifen	6.5	5.9	7.1	0.93	2 phase	y	84.6	14	95.2	4.7	1.9	6	4.10E+15	?	?	0.94	y
Mouridsen[19]	breast ER+<70	n	311	tamoxifen	tamoxifen	8	7.4	8.7	0.93	2 phase	n											y
Rose[464]	breast ER+	n	94	tamoxifen	tamoxifen	10.1	9.8	10.5	0.98	2 phase	y	43.4	27.3	67.8	4.6	3.3	6.2	17.4	14.2	28.8	0.99	y
Thigpen[465]	endometrial	n	68	tamoxifen	tamoxifen	2.5	2.4	2.7	0.99	2 phase	y	88.7	83.8	94.4	2	1.8	2.3	4.70E+10	3.6	?	0.97	y
Adachi[466]	breast	y/n	72	docetaxel	taxane	4.2	4	4.4	0.96	Con-low	n											n
Albain[127]	breast	y	263	paclitaxel	taxane	4.7	4.3	5.1	0.96	2 phase	n											y
Chan[51]	breast	y	161	docetaxel	taxane	5.3	5	5.7	0.91	Con-mod	n											n
Crump[50]‡	breast	n	112	anthra or taxane	taxane	9.7	9.2	10.1	0.97	2 phase	y	92.7	47.9	100	8.3	5.7	14.9	3.20E+15	?	?	0.97	y
Kramer[55]	breast	n	148	paclitaxel	taxane	3.4	3.1	3.6	0.88	Con-mod	n											n
Kruijtzer[467]	breast	y	82	docetaxel	taxane	4.5	4.1	4.9	0.93	Con-mod	n											n
Kruijtzer[467]	breast	y	96	docet-3rd line	taxane	4.3	4	4.6	0.94	Con-mod	n											n

Continuation of Supplementary Online Table 2. Exponential decay nonlinear regression analysis models for different therapies and references for sources of published PFS curves

author	Tumor type	Prior Rx	no. pts	Rx	class Rx	PFS $t_{1/2}$ mo.	LCI mo	UCI mo	R^2	PFS curve shape	2-phase by NLRA	% fast	LCI %	UCI %	fast $t_{1/2}$ mo.	LCI mo	UCI mo	slow $t_{1/2}$ mo.	LCI mo	UCI mo	R2	2 phase by shape or NLRA
Kruijtzer[467]	breast	y	52	docet-4th line	taxane	4.6	4.3	5	0.96	Con-mod	n											n
Miller[129]	breast	n	326	paclitaxel	taxane	6.6	6.3	6.9	0.98	S	n											n
Nabholtz[468]	breast	y	236	paclit 135 mg/m2	taxane	3.2	3.1	3.4	0.97	1 phase	n											n
Nabholtz[468]	breast	y	235	paclit 175 mg/m2	taxane	3.9	3.7	4.1	0.96	Con-low	n											n
Nabholtz[469]	breast	y	203	docetaxel	taxane	4	3.9	4.2	0.96	Con-low	n											n
Paridaens[56]	breast	n	166	paclitaxel	taxane	3.7	3.5	3.9	0.97	S	n											n
Seidman[470]	breast	y	350	paclit q1wk	taxane	11.6	10.8	12.5	0.94	S	n											n
Seidman[470]	breast	y	227	paclit q3wk	taxane	8.2	7.4	9.2	0.89	2 phase	n											y
Sjostrom[471]	breast	y	143	docetaxel	taxane	5.3	5	5.6	0.97	Con-low	n											n
Sledge[59]	breast	n	229	paclitaxel	taxane	6	5.9	8.8	0.99	1 phase	n											n
Sparano[132]	breast	y	373	docetaxel	taxane	5.9	5.6	6.2	0.97	Con-mod	n											n
Guan[136]	breast HER2+	n	222	paclitaxel	taxane	5.6	5.2	6	0.94	Con-low	n											n
Ferris[375]	head/neck	y	121	docet or MTX or cetux	taxane	2.5	2.4	2.7	0.91	Con-mod	n											n
Abe[167]	NSCLC	n	137	docetaxel	taxane	4.4	4.2	4.6	0.97	1 phase	n											n
Camps[472]	NSCLC	y	128	docet q1wk	taxane	3.3	3.1	3.6	0.9	S	n											n
Camps[472]	NSCLC	y	131	docet q3wk	taxane	3.7	3.5	3.9	0.93	1 phase	n											n
Fidias[410]	NSCLC	y	145	immediate docet	taxane	5.2	5	5.3	0.99	S	n											n
Fossella[473]	NSCLC	y	120	docet 75 mg/m2	taxane	2.6	2.2	2.3	0.96	1 phase	n											n
Fossella[473]	NSCLC	y	120	docet 100 mg/m2	taxane	2.3	2.2	2.4	0.97	1 phase	y	97.3	92.1	100	2.2	2	2.4	9.70E+11	4.1	infin	0.96	y
Garassino[303]	NSCLC	y	110	docetaxel	taxane	3.1	3.1	3.2	0.95	1 phase	n											n
Hanna[81]	NSCLC	y	288	docetaxel	taxane	3	2.9	3.1	0.98	1 phase	n											n
Herbst[474]	NSCLC	y	697	docetaxel	taxane	3.1	3	3.2	0.98	Con-mod	n											n
Herbst[392]	NSCLC	y	343	docetaxel	taxane	3.8	3.6	3.9	0.98	1 phase	n											n
Kawaguchi[268]	NSCLC	y	151	docetaxel	taxane	3.9	3.7	4.1	0.97	1 phase	n											n
Kim[269]	NSCLC	y	733	docetaxel	taxane	2.9	2.8	3.1	0.96	1 phase	n											n
Lee[475]	NSCLC	y	79	docetaxel	taxane	2.6	2.4	2.7	0.98	Con-mod	n											n
Lilenbaum[476]	NSCLC	n	55	docet q3wk	taxane	2.2	2.1	2.4	0.91	2 phase	y	65.8	53.5	78	1	0.9	1.3	11.3	6.1	78.5	0.99	y
Lilenbaum[476]	NSCLC	n	56	docet q1wk	taxane	2.5	2.4	2.6	0.98	2 phase	y	96.3	84.8	100	2.4	2.1	2.8	1.20E+11	2.8	infin	0.98	y
Maruyama[273]	NSCLC	y	187	docetaxel	taxane	2.6	2.5	2.7	0.96	1 phase	n											n
Pectasides[477]	NSCLC	y	65	docetaxel	taxane	4.5	4.1	5	0.81	Con-mod	n											n
Ramlau[182]	NSCLC	y	457	docetaxel	taxane	4	3.9	4.2	0.98	S	n											n
Ranson[400]	NSCLC	n	79	paclitaxel	taxane	3.9	3.7	4.1	0.94	S	n											n
Schuette[478]	NSCLC	y	105	docet q1wk	taxane	4.2	3.9	4.6	0.89	2 phase	y	80.7	67.2	94.2	2.6	2.2	3.2	3.20E+15	?	?	0.97	y
Schuette[478]	NSCLC	y	103	docet q3wk	taxane	5	4.5	5.5	0.86	2 phase	y	79.3	77.6	81	2.7	2.6	2.8	7.20E+12	301	infin	0.99	y
Tsukada[186]	NSCLC	n	63	docetaxel	taxane	3.6	3.3	4	0.92	Con-mod	n											n
Herbst[392]	NSCLC PDL1>50%	y	152	docetaxel	taxane	3.9	3.8	4.1	0.97	Con-low	n											n
Shaw[37]‡	NSCLC ALK+	y	174	pemet or docet	taxane	3.7	3.6	3.8	0.97	2 phase	y	97.3	92.6	?	3.5	3.1	3.8	1.40E+11	3.6	?	0.97	y
Douillard[302]	NSCLC EGFR WT	y	123	docetaxel	taxane	2.5	2.4	2.7	0.97	1 phase	n											n
Kawaguchi[268]	NSCLC EGFR WT	y	90	docetaxel	taxane	2.8	2.7	2.9	0.98	1 phase	n											n
Borghaei[386]	NSCLC nonsqu	y	290	docetaxel	taxane	3.7	3.5	3.9	0.98	Con-mod	n											n
Borghaei[386]	NSCLC PDL1<1%	y	101	docetaxel	taxane	3.8	3.6	4	0.96	Con-mod	n											n

Continuation of Supplementary Online Table 2. Exponential decay nonlinear regression analysis models for different therapies and references for sources of published PFS curves

author	Tumor type	Prior Rx	no. pts	Rx	class Rx	PFS $t_{1/2}$ mo.	LCI mo	UCI mo	R^2	PFS curve shape	2-phase by NLRA	% fast	LCI %	UCI %	fast $t_{1/2}$ mo.	LCI mo	UCI mo	slow $t_{1/2}$ mo.	LCI mo	UCI mo	R2	2 phase by shape or NLRA
Borghaei[386]	NSCLC PDL1<5%	y	138	docetaxel	taxane	3.9	3.7	4.1	0.96	Con-low	n											n
Borghaei[386]	NSCLC PDL1<10%	y	145	docetaxel	taxane	4.1	3.9	4.2	0.97	Con-low	n											n
Borghaei[386]	NSCLC PDL1>1%	y	123	docetaxel	taxane	4	3.9	4.2	0.95	Con-low	n											n
Borghaei[386]	NSCLC PDL1>5%	y	86	docetaxel	taxane	3.8	3.7	4	0.98	Con-low	n											n
Borghaei[386]	NSCLC PDL1>10%	y	101	docetaxel	taxane	3.6	4.5	3.8	0.97	Con-low	n											n
Brahmer[387]	NSCLC squam	y	137	docetaxel	taxane	3	2.8	3.3	0.94	Con-low	n											n
Brahmer[387]	NSCLC PDL1<1%	y	53	docetaxel	taxane	3.2	2.9	3.6	0.87	Con-mod	n											n
Brahmer[387]	NSCLC PDL1<5%	y	69	docetaxel	taxane	6.5	6.3	6.7	0.98	1 phase	n											n
Brahmer[387]	NSCLC PDL1<10%	y	75	docetaxel	taxane	3	2.8	3.3	0.93	Con-mod	n											n
Brahmer[387]	NSCLC PDL1>1%	y	56	docetaxel	taxane	3.1	2.9	3.3	0.94	Con-low	n											n
Fehrenbacher[385]	NSCLC	y	143	docetaxel	taxane	3.6	3.4	3.7	0.98	1 phase	y	93.6	88.8	98.5	3.1	2.8	3.4	1.50E+11	17.5	infin	0.99	y
Fehrenbacher[385]	NSCLC TC-IC 1-3	y	102	docetaxel	taxane	3.4	3.2	3.6	0.97	2 phase	y	92.5	85.1	99.9	2.9	2.5	3.4	3.10E+12	8.1	infin	0.97	y
Fehrenbacher[385]	NSCLC TC-IC 2-3	y	55	docetaxel	taxane	3	2.8	3.2	0.96	S	n											n
Buda[479]	ovary	y	106	paclitaxel	taxane	7.3	7	7.7	0.97	Con-low	n											n
Eisenhauer[480]	ovary	y	187	paclit 3 hr infusion	taxane	4	3.8	4.2	0.98	S	n											n
Eisenhauer[480]	ovary	y	204	paclit-24 hr infusion	taxane	3.6	3.4	3.8	0.95	Con-low	n											n
Eisenhauer[480]	ovary	y	199	paclit 135 mg/m2	taxane	3.1	3	3.3	0.98	1 phase	n											n
Eisenhauer[480]	ovary	y	192	paclit 175 mg/m2	taxane	3.8	3.7	4	0.98	Con-low	n											n
Muggia[427]	ovary	n	213	paclitaxel	taxane	11.2	10.9	11.5	0.98	2 phase	y	33.2	20.6	56.8	4.6	3.3	6.5	16	14.1	21.3	0.99	y
Pujade-Lauraine[62]‡	ovary	y	182	PEG doxo or paclit or topo	taxane	3.4	3.1	3.7	0.93	S	n											n
Rosenberg[481]	ovary	y	105	paclit q1wk	taxane	7.7	7.2	8.4	0.93	Con-low	n											n
Rosenberg[481]	ovary	y	103	paclit q3wk	taxane	8.7	8.1	9.3	0.94	Con-low	n											n
ten Bokkel[482]	ovary	y	114	paclitaxel	taxane	3.2	3	3.4	0.95	Con-low	n											n
Trope[483]	ovary PS0	y	69	paclitaxel	taxane	4.9	4.4	5.5	0.88	Con-mod	n											n
Trope[483]	ovary PS1	y	62	paclitaxel	taxane	4	3.6	4.3	0.85	Con-mod	n											n
Bellmunt[370]‡	urothelial	y	272	taxane or vinflunine	taxane	3.4	3.1	3.6	0.95	1 phase	n											n
Haller[145]	colon	y	310	irinotecan	Top I	2.8	2.8	2.9	0.99	1 phase	n											n
Kim[148]	colon	y	245	irinotecan	Top I	4.1	4	4.2	0.99	Con-low	n											n
Mendez[484]	colon	y	115	irinotecan	Top I	5.7	5.5	6	0.96	1 phase	n											n
Seymour[485]	colon	y	230	irinotecan	Top I	4.3	4.1	4.6	0.91	Con-mod	n											n
Sobrero[153]	colon	y	650	irinotecan	Top I	2.6	2.4	2.8	0.94	Con-mod	n											n
Higuchi[486]	gastric	y	63	irinotecan	Top I	10	9.5	10.4	0.94	Con-mod	n											n
Nishikawa[487]	gastric	y	84	irinotecan	Top I	4	3.8	4.3	0.95	Con-mod	n											n
Tanabe[488]	gastric	y	148	irinotecan	Top I	3.5	3.2	3.7	0.93	Con-mod	n											n
Pujade-Lauraine[62]‡	ovary	y	182	PEG doxo or paclit or topo	Top I	3.4	3.1	3.7	0.93	S	n											n
von Pawel[49]	SCLC	y	213	topotecan	Top I	3.3	3.1	3.5	0.95	Con-mod	n											n
Gligorov[489]	breast	Mnt	94	bevacizumab	VEGF mono	5.3	5	5.6	0.97	S	n											n
Johnsson[490]	colon	Mnt	79	bevacizumab	VEGF mono	4.6	4.2	5.1	0.87	Con-mod	n											n
Tournigand[491]	colon	Mnt	228	bevacizumab	VEGF mono	4.8	4.5	5.1	0.96	S	n											n

Continuation of Supplementary Online Table 2. Exponential decay nonlinear regression analysis models for different therapies and references for sources of published PFS curves

author	Tumor type	Prior Rx	no. pts	Rx	class Rx	PFS $t_{1/2}$ mo.	LCI mo	UCI mo	R^2	PFS curve shape	2-phase by NLRA	% fast	LCI %	UCI %	fast $t_{1/2}$ mo.	LCI mo	UCI mo	slow $t_{1/2}$ mo.	LCI mo	UCI mo	R2	2 phase by shape or NLRA
Cloughesy[492]	glioma	Mnt	65	bevacizumab	VEGF mono	3.9	3.6	4.2	0.89	S	n											n
Friedman[493]	glioma	y	85	bevacizumab	VEGF mono	4.3	4.1	4.5	0.95	Con-low	n											n
Zhu[408]	head/neck	y	283	ramucirumab	VEGF mono	3.9	3.7	4.2	0.96	2 phase	y	94.4	90.6	98.6	3.5	3.1	3.9	4.00E+10	4.4	?	0.96	y
Carvajal[494]	melanoma	n	50	ramucirumab	VEGF mono	2.1	1.9	2.3	0.86	S	n											n
Barlesi[495]	NSCLC	Mnt	125	bevacizumab	VEGF mono	4.2	4	4.4	0.96	Con-low	n											n
Johnson[100]	NSCLC	Mnt	373	bevacizumab	VEGF mono	3.7	3.5	3.8	0.97	2 phase	y	96.3	92.8	?	3.4	3.1	3.8	1.10E+10	3.5	?	0.97	y
Tew[496]	ovary	y	75	bevacizumab	VEGF mono	5.4	5.1	5.7	0.94	S	n											n
Bukowski[497]	renal	n	53	bevacizumab	VEGF mono	9.1	8.7	9.5	0.95	S	n											n
Flaherty[498]	renal	y/n	89	bevacizumab	VEGF mono	7.2	6.9	7.6	0.97	2 phase	y	12.7	10.9	14.6	0.5	0.3	0.7	8.6	8.4	8.9	0.99	y
Hainsworth[499]	renal	y/n	58	bev q1wk	VEGF mono	5.7	5.3	6.1	0.95	1 phase	n											n
Hainsworth[499]	renal	y/n	61	bev q2wk	VEGF mono	6.7	6.4	7.1	0.95	2 phase	y	61.1	33.6	85.4	3.4	2.1	?	18.4	10.5	?	0.98	y
Yang[500]	renal	y	37	bev-low dose	VEGF mono	3.3	2.9	3.7	0.85	Con-mod	n											n
Yang[500]	renal	y	39	bev-high dose	VEGF mono	5.5	5.1	5.9	0.92	Con-low	n											n
Fumoleau[501]	breast	n	157	vinorelbine	vinorelbine	5.1	4.9	5.2	0.98	Con-mod	n											n
Garcia-Conde[502]	breast	n	50	vinorelbine	vinorelbine	4.5	4.4	4.5	0.99	2 phase	y	94.3	89.2	?	4.1	3.7	?	4.10E+10	?	?	0.99	y
Gasparini[503]	breast	y	70	vinorelbine	vinorelbine	5.3	4.8	5.9	0.85	Con-mod	n											n
Jones[504]	breast	y	115	vinorelbine	vinorelbine	2.8	2.7	3	0.93	Con-low	n											n
Martin[505]	breast	y	127	vinorelbine	vinorelbine	4.3	4	4.6	0.93	Con-mod	n											n
Toi[506]	breast	y	50	vinorelbine	vinorelbine	3.4	3.2	3.5	0.94	Con-mod	n											n
Chen[507]	NSCLC	n	56	vinorelbine	vinorelbine	3	2.8	3.3	0.92	2 phase	n											y
Depierre[508]	NSCLC	n	115	vinorelbine	vinorelbine	3.1	2.8	3.6	0.82	Con-mod	n											n
Crino[257]	NSCLC	n	99	vinorelbine	vinorelbine	3.1	3	3.1	0.99	1 phase	n											n

† brain metastases

‡ included in more than 1 category

§ different sequencing methods found additional mutants

¶ could include chemotherapy

Abbreviations	
abirat	abiraterone
adeno	adenocarcinoma
aflib	aflibercept
AI	aromatase inhibitor
ALK	anaplastic lymphoma kinase
amrub	amrubicin
anastr	anastrazole
angio inh	Oral angiogenesis inhibitor
anthra	anthracycline
antiandro	antiandrogen
atezol	atezolizumab
AUC	area under the curve
axit	axitinib
bev	bevacizumab

Continuation of Supplementary Online Table 2. Exponential decay nonlinear regression analysis models for different therapies and references for sources of published PFS curves	
BRAF TKI-m	BRAF tyrosine kinase inhibitor in patients with BRAF mutations
breast ER+	breast estrogen receptor positive
breast ER+/?	breast estrogen receptor positive or unknown
breast ER+/P-a	breast ER+/PIK3Ca activated
breast ER+/PM	breast ER+/postmenopausal
breast ER+/P-WT	breast ER+/PIK3Ca wild type
breast GSG2	breast gene signature group 2
breast HER2 F+	breast HER2 FISH+
breast HER2+	breast HER2 positive
breast HER2+/As	breast HER2+/Asian
breast PM	breast postmenopausal
breast triple-	breast triple negative
BSC	best supportive care
cape	capecitabine
carbo	carboplatin
cedir	cedirinib
cetux	cetuximab
chemo	chemotherapy
cisp	cisplatin
CMF	cyclophosphamide + methotrexate + 5-fluorouracil
cobim	cobimetinib
colon EG high	colon: high expression of epidermal growth factor receptor
colon EG IHC+	colon epidermal growth factor receptor positive by immunohistochemistry
colon KRAS WT	colon KRAS wild type
colon KRAS-m	colon: KRAS mutant
colon T-T geno	colon KRAS T-T genotype
combo AI	Combination of aromatase inhibitor with other agents
combo EGFR-u	Combination of EGFR TKI with other agents in patients unselected for EGFR mutation
combo HER2	Combination of HER2 monoclonal antibody with other agent in breast cancer HER2+ patients
combo Ipi/other	Combination of ipilimumab and drug other than PD1/PDL1 monoclonal
combo PD1/chem	Combination of PD1/PDL1 monoclonal with chemotherapy
combo PD1/ipi	Combination of PD1/PDL1 monoclonal with ipilimumab
combo plat ovary	Platinum combination as first line therapy for ovarian carcinoma
Con-high	high degree of convexity
Con-low	low degree of convexity
Con-mod	moderate degree of convexity
contin	continuous
CTX	cyclophosphamide
dabra	dabrafenib
dacarb	dacarbazine
docet	docetaxel
doxo	doxorubicin
EGFR mono	EGFR monoclonal antibody
EGFR TKI-m	EGFR tyrosine kinase inhibitor in NSCLC patients with mutated EGFR
EGFR TKI-u	EGFR tyrosine kinase inhibitor in NSCLC patients unselected for presence of EGFR mutation (some with and some without mutation)
EGFR TKI-WT	EGFR tyrosine kinase inhibitor in NSCLC patients with wild type EGFR

Continuation of Supplementary Online Table 2. Exponential decay nonlinear regression analysis models for different therapies and references for sources of published PFS curves	
erlot	erlotinib
etop	etoposide
evero	everolimus
exem	exemestane
figit	figitumumab
Fluoropyr	fluoropyrimidine
FOLFIRI	5-fluorouracil + folinic acid + irinotecan
FOLFIRINOX	5-fluorouracil + folinic acid + irinotecan + oxaliplatin
FOLFOX	5-fluorouracil + folinic acid + oxaliplatin
FOLFOXIRI	5-fluorouracil + folinic acid + oxaliplatin + irinotecan
FU	5-fluorouracil
fulv	fulvestrant
Fulvest	fulvestrant
gastric HER2+	gastric HER2 positive
gastric/GEJ	gastric or gastroesophageal junction
Gem	gemcitabine
GI	Gastrointestinal
GIST	gastrointestinal stromal tumor
head/neck-A	Head and neck carcinoma in Asians
hepatocell	hepatocellular
HER2 mono	HER2 monoclonal antibody
HPV-	negative for human papilloma virus
ICONG	International Collaborative Ovarian Neoplasm Group
IFN	interferon
IL2	interleukin-2
infin	infinity
ipi	ipilimumab
irino	irinotecan
lapat	lapatinib
LCI	lower 95% confidence interval
letroz	letrozole
lip doxo	liposomal doxorubicin
mel/NSCLC- no liver	melanoma or NSCLC- no liver metastases
mel/NSCLC-liver	melanoma or NSCLC-liver metastases
melanoma B-m	melanoma BRAF mutant
melanoma B-WT	melanoma BRAF WT
melanoma-C4-6	melanoma-cohorts 4-6
mel-extracran	melanoma-extracranial sites
mito	mitomycin
mitox	mitoxantrone
Mnt	maintenance post induction therapy
mo.	months
modif	modified
motes	motesanib
MSI-H	high microsatellite instability
mTOR inh	mTOR inhibitor

Continuation of Supplementary Online Table 2. Exponential decay nonlinear regression analysis models for different therapies and references for sources of published PFS curves

MTX	methotrexate
n	no
neuroend	neuroendocrine
nivol	nivolumab
nonsmoker	never smoker or light smoker
NSCLC	non small cell lung cancer
NSCLC ALK+	NSCLC positive for ALK fusion gene
NSCLC EGFR WT	NSCLC with wild type EGFR
NSCLC EGFR-m	NSCLC positive for mutation in epidermal growth factor receptor
NSCLC EGFR-u	NSCLC unselected for EGFR mutation (mixture of patients with and without mutations)
NSCLC nonsqu	NSCLC non-squamous
NSCLC squam	NSCLC squamous
ovary PS0	ovary-performance status 0
ovary PS1	ovary-performance status 1
oxal	oxaliplatin
paclit	paclitaxel
panit	panitumumab
PCV	procarbazine + CCNU + vincristine
PD1/PDL1	PD1/PDL1 monoclonal antibodies
PDL1-	negative for PDL1 by immunohistochemistry
PDL1+	positive for PDL1 by immunohistochemistry
PEG doxo	pegylated doxorubicin
pembro	pembrolizumab
pemet	pemetrexed
pertuz	pertuzumab
Plat	cisplatin or carboplatin
Plat 1st Rx ovary	cisplatin or carboplatin as first line therapy for carcinomas of the ovary or seminoma
pred	prednisone
pts	patients
RAF non-GIST	RAF inhibitors in tumors other than gastrointestinal stromal tumors
Rx	treatment
SCLC	small cell lung cancer
small mol	Other small molecules
soraf	sorafenib
streptoz	streptozotocin
sunit	sunitinib
Sunit non-GIST	Sunitinib in tumors other than gastrointestinal stromal tumors
TC&IC 0	0 PDL1 staining in immune cells and tumor cells by immunohistochemistry
TC-IC 1-3	1-3+ PDL1 staining in immune cells or tumor cells by immunohistochemistry
TC-IC 2	2+ PDL1 staining in immune cells or tumor cells by immunohistochemistry
TC-IC 2-3	2-3+ PDL1 staining in immune cells or tumor cells by immunohistochemistry
TC-IC 3	3+ PDL1 staining in immune cells or tumor cells by immunohistochemistry
temoz	temozolamide
tivant	tivantinib
TKI	tyrosine kinase inhibitor
TMB high	high tumor mutation burden

Continuation of Supplementary Online Table 2. Exponential decay nonlinear regression analysis models for different therapies and references for sources of published PFS curves	
TNF	tumor necrosis factor
Top I	topoisomerase I inhibitor
Top II	topoisomerase II inhibitor
topot	topotecan
tramet	trametinib
trastuz	trastuzumab
UCI	upper 95% confidence interval
VEGF mono	VEGF monoclonal antibody (bevacizumab or ramucirumab)
vemur	vemurafenib
vinbl	vinblastine
vinor	vinorelbine
XELOX	capecitabine + folinic acid + oxaliplatin
XRT	radiotherapy
y	yes
y/n	yes or no

References for sources of PFS curves

1. Ellis MJ, Llombart-Cussac A, Feltl D, et al. Fulvestrant 500 mg Versus Anastrozole 1 mg for the First-Line Treatment of Advanced Breast Cancer: Overall Survival Analysis From the Phase II FIRST Study. J Clin Oncol 2015;33:3781-7.
2. Howell A, Robertson JF, Quaresma Albano J, et al. Fulvestrant, formerly ICI 182,780, is as effective as anastrozole in postmenopausal women with advanced breast cancer progressing after prior endocrine treatment. J Clin Oncol 2002;20:3396-403.
3. Kaufman B, Mackey JR, Clemens MR, et al. Trastuzumab plus anastrozole versus anastrozole alone for the treatment of postmenopausal women with human epidermal growth factor receptor 2-positive, hormone receptor-positive metastatic breast cancer: results from the randomized phase III TAnDEM study. J Clin Oncol 2009;27:5529-37.
4. Mehta RS, Barlow WE, Albain KS, et al. Combination anastrozole and fulvestrant in metastatic breast cancer. N Engl J Med 2012;367:435-44.
5. Osborne CK, Pippen J, Jones SE, et al. Double-blind, randomized trial comparing the efficacy and tolerability of fulvestrant versus anastrozole in postmenopausal women with advanced breast cancer progressing on prior endocrine therapy: results of a North American trial. J Clin Oncol 2002;20:3386-95.
6. Robertson JF, Llombart-Cussac A, Rolski J, et al. Activity of fulvestrant 500 mg versus anastrozole 1 mg as first-line treatment for advanced breast cancer: results from the FIRST study. J Clin Oncol 2009;27:4530-5.
7. Xu B, Jiang Z, Shao Z, et al. Fulvestrant 250 mg versus anastrozole for Chinese patients with advanced breast cancer: results of a multicentre, double-blind, randomised phase III trial. Cancer Chemother Pharmacol 2011;67:223-30.
8. Goetz MP, Toi M, Campone M, et al. MONARCH 3: Abemaciclib As Initial Therapy for Advanced Breast Cancer. J Clin Oncol 2017;35:3638-46.
9. Tominaga T, Adachi I, Sasaki Y, et al. Double-blind randomised trial comparing the non-steroidal aromatase inhibitors letrozole and fadrozole in postmenopausal women with advanced breast cancer. Ann Oncol 2003;14:62-70.
10. Dickler MN, Barry WT, Cirrincione CT, et al. Phase III Trial Evaluating Letrozole As First-Line Endocrine Therapy With or Without Bevacizumab for the Treatment of Postmenopausal Women With Hormone Receptor-Positive Advanced-Stage Breast Cancer: CALGB 40503 (Alliance). J Clin Oncol 2016;34:2602-9.
11. Finn RS, Crown JP, Lang I, et al. The cyclin-dependent kinase 4/6 inhibitor palbociclib in combination with letrozole versus letrozole alone as first-line treatment of oestrogen receptor-positive, HER2-negative, advanced breast cancer (PALOMA-1/TRIO-18): a randomised phase 2 study. Lancet Oncol 2015;16:25-35.
12. Finn RS, Martin M, Rugo HS, et al. Palbociclib and Letrozole in Advanced Breast Cancer. N Engl J Med 2016;375:1925-36.
13. Goss P, Bondarenko IN, Manikhas GN, et al. Phase III, double-blind, controlled trial of atamestane plus toremifene compared with letrozole in postmenopausal women with advanced receptor-positive breast cancer. J Clin Oncol 2007;25:4961-6.
14. Hortobagyi GN, Stemmer SM, Burris HA, et al. Updated results from MONALEESA-2, a phase III trial of first-line ribociclib plus letrozole versus placebo plus letrozole in hormone receptor-positive, HER2-negative advanced breast cancer. Ann Oncol 2018;29:1541-7.
15. Johnston SR, Semiglazov VF, Manikhas GM, et al. A phase II, randomized, blinded study of the farnesyltransferase inhibitor tipifarnib combined with letrozole in the treatment of advanced breast cancer after antiestrogen therapy. Breast Cancer Res Treat 2008;110:327-35.
16. Johnston S, Pippen J, Jr., Pivot X, et al. Lapatinib combined with letrozole versus letrozole and placebo as first-line therapy for postmenopausal hormone receptor-positive metastatic breast cancer. J Clin Oncol 2009;27:5538-46.
17. Mouridsen H, Gershanovich M, Sun Y, et al. Superior efficacy of letrozole versus tamoxifen as first-line therapy for postmenopausal women with advanced breast cancer: results of a phase III study of the International Letrozole Breast Cancer Group. J Clin Oncol 2001;19:2596-606.
18. Mouridsen H, Gershanovich M, Sun Y, et al. Phase III study of letrozole versus tamoxifen as first-line therapy of advanced breast cancer in postmenopausal women: analysis of survival and update of efficacy from the International Letrozole Breast Cancer Group. J Clin Oncol 2003;21:2101-9.
19. Mouridsen H, Chaudri-Ross HA. Efficacy of first-line letrozole versus tamoxifen as a function of age in postmenopausal women with advanced breast cancer. Oncologist 2004;9:497-506.
20. O'Shaughnessy J, Petrakova K, Sonke GS, et al. Ribociclib plus letrozole versus letrozole alone in patients with de novo HR+, HER2- advanced breast cancer in the randomized MONALEESA-2 trial. Breast Cancer Res Treat 2018;168:127-34.
21. Sonke GS, Hart LL, Campone M, et al. Ribociclib with letrozole vs letrozole alone in elderly patients with hormone receptor-positive, HER2-negative breast cancer in the randomized MONALEESA-2 trial. Breast Cancer Res Treat 2018;167:659-69.
22. Wolff AC, Lazar AA, Bondarenko I, et al. Randomized phase III placebo-controlled trial of letrozole plus oral temsirolimus as first-line endocrine therapy in postmenopausal women with locally advanced or metastatic breast cancer. J Clin Oncol 2013;31:195-202.
23. Dombernowsky P, Smith I, Falkson G, et al. Letrozole, a new oral aromatase inhibitor for advanced breast cancer: double-blind randomized trial showing a dose effect and improved efficacy and tolerability compared with megestrol acetate. J Clin Oncol 1998;16:453-61.
24. Gershanovich M, Chaudri HA, Campos D, et al. Letrozole, a new oral aromatase inhibitor: randomised trial comparing 2.5 mg daily, 0.5 mg daily and aminoglutethimide in postmenopausal women with advanced breast cancer. Letrozole International Trial Group (AR/BC3). Ann Oncol 1998;9:639-45.
25. Hida T, Nokihara H, Kondo M, et al. Alectinib versus crizotinib in patients with ALK-positive non-small-cell lung cancer (J-ALEX): an open-label, randomised phase 3 trial. Lancet 2017;390:29-39.
26. Ou SH, Ahn JS, De Petris L, et al. Alectinib in Crizotinib-Refractory ALK-Rearranged Non-Small-Cell Lung Cancer: A Phase II Global Study. J Clin Oncol 2016;34:661-8.
27. Peters S, Camidge DR, Shaw AT, et al. Alectinib versus Crizotinib in Untreated ALK-Positive Non-Small-Cell Lung Cancer. N Engl J Med 2017;377:829-38.
28. Shaw AT, Gandhi L, Gadgeel S, et al. Alectinib in ALK-positive, crizotinib-resistant, non-small-cell lung cancer: a single-group, multicentre, phase 2 trial. Lancet Oncol 2016;17:234-42.
29. Camidge DR, Kim HR, Ahn MJ, et al. Brigatinib versus Crizotinib in ALK-Positive Non-Small-Cell Lung Cancer. N Engl J Med 2018;379:2027-39.
30. Kim DW, Tiseo M, Ahn MJ, et al. Brigatinib in Patients With Crizotinib-Refractory Anaplastic Lymphoma Kinase-Positive Non-Small-Cell Lung Cancer: A Randomized, Multicenter Phase II Trial. J Clin Oncol 2017;35:2490-8.
31. Crino L, Ahn MJ, De Marinis F, et al. Multicenter Phase II Study of Whole-Body and Intracranial Activity With Ceritinib in Patients With ALK-Rearranged Non-Small-Cell Lung Cancer Previously Treated With Chemotherapy and Crizotinib: Results From ASCEND-2. J Clin Oncol 2016;34:2866-73.
32. Kim DW, Mehra R, Tan DSW, et al. Activity and safety of ceritinib in patients with ALK-rearranged non-small-cell lung cancer (ASCEND-1): updated results from the multicentre, open-label, phase 1 trial. Lancet Oncol 2016;17:452-63.
33. Shaw AT, Kim DW, Mehra R, et al. Ceritinib in ALK-rearranged non-small-cell lung cancer. N Engl J Med 2014;370:1189-97.
34. Shaw AT, Kim TM, Crino L, et al. Ceritinib versus chemotherapy in patients with ALK-rearranged non-small-cell lung cancer previously given chemotherapy and crizotinib (ASCEND-5): a randomised, controlled, open-label, phase 3 trial. Lancet Oncol 2017;18:874-86.
35. Camidge DR, Bang YJ, Kwak EL, et al. Activity and safety of crizotinib in patients with ALK-positive non-small-cell lung cancer: updated results from a phase 1 study. Lancet Oncol 2012;13:1011-9.
36. Nishio M, Kim DW, Wu YL, et al. Crizotinib versus Chemotherapy in Asian Patients with ALK-Positive Advanced Non-small Cell Lung Cancer. Cancer Res Treat 2018;50:691-700.
37. Shaw AT, Kim DW, Nakagawa K, et al. Crizotinib versus chemotherapy in advanced ALK-positive lung cancer. N Engl J Med 2013;368:2385-94.
38. Shaw AT, Ou SH, Bang YJ, et al. Crizotinib in ROS1-rearranged non-small-cell lung cancer. N Engl J Med 2014;371:1963-71.
39. Solomon BJ, Mok T, Kim DW, et al. First-line crizotinib versus chemotherapy in ALK-positive lung cancer. N Engl J Med 2014;371:2167-77.
40. Cainap C, Qin S, Huang WT, et al. Linifanib versus Sorafenib in patients with advanced hepatocellular carcinoma: results of a randomized phase III trial. J Clin Oncol 2015;33:172-9.
41. Kang YK, Yau T, Park JW, et al. Randomized phase II study of axitinib versus placebo plus best supportive care in second-line treatment of advanced hepatocellular carcinoma. Ann Oncol 2015;26:2457-63.
42. Li J, Qin S, Xu J, et al. Randomized, Double-Blind, Placebo-Controlled Phase III Trial of Apatinib in Patients With Chemotherapy-Refractory Advanced or Metastatic Adenocarcinoma of the Stomach or Gastroesophageal Junction. J Clin Oncol 2016;34:1448-54.
43. Llovet JM, Decaens T, Raoul JL, et al. Brivanib in patients with advanced hepatocellular carcinoma who were intolerant to sorafenib or for whom sorafenib failed: results from the randomized phase III BRISK-PS study. J Clin Oncol 2013;31:3509-16.
44. Motzer RJ, Nosov D, Eisen T, et al. Tivozanib versus sorafenib as initial targeted therapy for patients with metastatic renal cell carcinoma: results from a phase III trial. J Clin Oncol 2013;31:3791-9.
45. Motzer RJ, Hutson TE, Glen H, et al. Lenvatinib, everolimus, and the combination in patients with metastatic renal cell carcinoma: a randomised, phase 2, open-label, multicentre trial. Lancet Oncol 2015;16:1473-82.

46. Schlumberger M, Tahara M, Wirth LJ, et al. Lenvatinib versus placebo in radioiodine-refractory thyroid cancer. N Engl J Med 2015;372:621-30.
47. Sternberg CN, Davis ID, Mardiak J, et al. Pazopanib in locally advanced or metastatic renal cell carcinoma: results of a randomized phase III trial. J Clin Oncol 2010;28:1061-8.
48. van der Graaf WT, Blay JY, Chawla SP, et al. Pazopanib for metastatic soft-tissue sarcoma (PALETTE): a randomised, double-blind, placebo-controlled phase 3 trial. Lancet 2012;379:1879-86.
49. von Pawel J, Jotte R, Spigel DR, et al. Randomized phase III trial of amrubicin versus topotecan as second-line treatment for patients with small-cell lung cancer. J Clin Oncol 2014;32:4012-9.
50. Crump M, Gluck S, Tu D, et al. Randomized trial of high-dose chemotherapy with autologous peripheral-blood stem-cell support compared with standard-dose chemotherapy in women with metastatic breast cancer: NCIC MA.16. J Clin Oncol 2008;26:37-43.
51. Chan S, Friedrichs K, Noel D, et al. Prospective randomized trial of docetaxel versus doxorubicin in patients with metastatic breast cancer. J Clin Oncol 1999;17:2341-54.
52. Gish RG, Porta C, Lazar L, et al. Phase III randomized controlled trial comparing the survival of patients with unresectable hepatocellular carcinoma treated with nolatrexed or doxorubicin. J Clin Oncol 2007;25:3069-75.
53. Henderson IC, Allegra JC, Woodcock T, et al. Randomized clinical trial comparing mitoxantrone with doxorubicin in previously treated patients with metastatic breast cancer. J Clin Oncol 1989;7:560-71.
54. Ingle JN, Pfeifle DM, Green SJ, et al. Randomized clinical trial of doxorubicin alone or combined with mitolactol in women with advanced breast cancer and prior chemotherapy exposure. Am J Clin Oncol 1985;8:275-82.
55. Kramer JA, Curran D, Piccart M, et al. Randomised trial of paclitaxel versus doxorubicin as first-line chemotherapy for advanced breast cancer: quality of life evaluation using the EORTC QLQ-C30 and the Rotterdam symptom checklist. Eur J Cancer 2000;36:1488-97.
56. Paridaens R, Biganzoli L, Bruning P, et al. Paclitaxel versus doxorubicin as first-line single-agent chemotherapy for metastatic breast cancer: a European Organization for Research and Treatment of Cancer Randomized Study with cross-over. J Clin Oncol 2000;18:724-33.
57. Qin S, Bai Y, Lim HY, et al. Randomized, multicenter, open-label study of oxaliplatin plus fluorouracil/leucovorin versus doxorubicin as palliative chemotherapy in patients with advanced hepatocellular carcinoma from Asia. J Clin Oncol 2013;31:3501-8.
58. Reyno L, Seymour L, Tu D, et al. Phase III study of N,N-diethyl-2-[4-(phenylmethyl) phenoxy]ethanamine (BMS-217380-01) combined with doxorubicin versus doxorubicin alone in metastatic/recurrent breast cancer: National Cancer Institute of Canada Clinical Trials Group Study MA.19. J Clin Oncol 2004;22:269-76.
59. Sledge GW, Neuberg D, Bernardo P, et al. Phase III trial of doxorubicin, paclitaxel, and the combination of doxorubicin and paclitaxel as front-line chemotherapy for metastatic breast cancer: an intergroup trial (E1193). J Clin Oncol 2003;21:588-92.
60. Thigpen JT, Blessing JA, DiSaia PJ, Yordan E, Carson LF, Evers C. A randomized comparison of doxorubicin alone versus doxorubicin plus cyclophosphamide in the management of advanced or recurrent endometrial carcinoma: A Gynecologic Oncology Group study. J Clin Oncol 1994;12:1408-14.
61. Thigpen JT, Brady MF, Homesley HD, et al. Phase III trial of doxorubicin with or without cisplatin in advanced endometrial carcinoma: a gynecologic oncology group study. J Clin Oncol 2004;22:3902-8.
62. Pujade-Lauraine E, Hilpert F, Weber B, et al. Bevacizumab combined with chemotherapy for platinum-resistant recurrent ovarian cancer: The AURELIA open-label randomized phase III trial. J Clin Oncol 2014;32:1302-8.
63. Judson I, Verweij J, Gelderblom H, et al. Doxorubicin alone versus intensified doxorubicin plus ifosfamide for first-line treatment of advanced or metastatic soft-tissue sarcoma: a randomised controlled phase 3 trial. Lancet Oncol 2014;15:415-23.
64. Lorigan P, Verweij J, Papai Z, et al. Phase III trial of two investigational schedules of ifosfamide compared with standard-dose doxorubicin in advanced or metastatic soft tissue sarcoma: a European Organisation for Research and Treatment of Cancer Soft Tissue and Bone Sarcoma Group Study. J Clin Oncol 2007;25:3144-50.
65. Martin-Broto J, Pousa AL, de Las Penas R, et al. Randomized Phase II Study of Trabectedin and Doxorubicin Compared With Doxorubicin Alone as First-Line Treatment in Patients With Advanced Soft Tissue Sarcomas: A Spanish Group for Research on Sarcoma Study. J Clin Oncol 2016;34:2294-302.
66. Maurel J, Lopez-Pousa A, de Las Penas R, et al. Efficacy of sequential high-dose doxorubicin and ifosfamide compared with standard-dose doxorubicin in patients with advanced soft tissue sarcoma: an open-label randomized phase II study of the Spanish group for research on sarcomas. J Clin Oncol 2009;27:1893-8.
67. Tap WD, Papai Z, Van Tine BA, et al. Doxorubicin plus evofosfamide versus doxorubicin alone in locally advanced, unresectable or metastatic soft-tissue sarcoma (TH CR-406/SARC021): an international, multicentre, open-label, randomised phase 3 trial. Lancet Oncol 2017;18:1089-103.
68. Ryan CJ, Smith MR, de Bono JS, et al. Abiraterone in metastatic prostate cancer without previous chemotherapy. N Engl J Med 2013;368:138-48.
69. de Bono JS, Logothetis CJ, Molina A, et al. Abiraterone and increased survival in metastatic prostate cancer. N Engl J Med 2011;364:1995-2005.
70. Sweeney CJ, Chen YH, Carducci M, et al. Chemohormonal Therapy in Metastatic Hormone-Sensitive Prostate Cancer. N Engl J Med 2015;373:737-46.
71. Beer TM, Armstrong AJ, Rathkopf DE, et al. Enzalutamide in metastatic prostate cancer before chemotherapy. N Engl J Med 2014;371:424-33.
72. Scher HI, Fizazi K, Saad F, et al. Increased survival with enzalutamide in prostate cancer after chemotherapy. N Engl J Med 2012;367:1187-97.
73. Fizazi K, Jones R, Oudard S, et al. Phase III, randomized, double-blind, multicenter trial comparing orteronel (TAK-700) plus prednisone with placebo plus prednisone in patients with metastatic castration-resistant prostate cancer that has progressed during or after docetaxel-based therapy: ELM-PC 5. J Clin Oncol 2015;33:723-31.
74. Ardizzoni A, Tiseo M, Boni L, et al. Pemetrexed versus pemetrexed and carboplatin as second-line chemotherapy in advanced non-small-cell lung cancer: results of the GOIRC 02-2006 randomized phase II study and pooled analysis with the NVALT7 trial. J Clin Oncol 2012;30:4501-7.
75. Boeck S, Weigang-Kohler K, Fuchs M, et al. Second-line chemotherapy with pemetrexed after gemcitabine failure in patients with advanced pancreatic cancer: a multicenter phase II trial. Ann Oncol 2007;18:745-51.
76. Ciuleanu T, Brodowicz T, Zielinski C, et al. Maintenance pemetrexed plus best supportive care versus placebo plus best supportive care for non-small-cell lung cancer: a randomised, double-blind, phase 3 study. Lancet 2009;374:1432-40.
77. Cullen MH, Zatloukal P, Sorenson S, et al. A randomized phase III trial comparing standard and high-dose pemetrexed as second-line treatment in patients with locally advanced or metastatic non-small-cell lung cancer. Ann Oncol 2008;19:939-45.
78. de Boer RH, Arrieta O, Yang CH, et al. Vandetanib plus pemetrexed for the second-line treatment of advanced non-small-cell lung cancer: a randomized, double-blind phase III trial. J Clin Oncol 2011;29:1067-74.
79. Dittrich C, Papai-Szekely Z, Vinolas N, et al. A randomised phase II study of pemetrexed versus pemetrexed+erlotinib as second-line treatment for locally advanced or metastatic non-squamous non-small cell lung cancer. Eur J Cancer 2014;50:1571-80.
80. Gautschi O, Rothschild SI, Li Q, et al. Bevacizumab Plus Pemetrexed Versus Pemetrexed Alone as Maintenance Therapy for Patients With Advanced Nonsquamous Non-Small-cell Lung Cancer: Update From the Swiss Group for Clinical Cancer Research (SAKK) 19/09 Trial. Clin Lung Cancer 2017;18:303-9.
81. Hanna N, Shepherd FA, Fossella FV, et al. Randomized phase III trial of pemetrexed versus docetaxel in patients with non-small-cell lung cancer previously treated with chemotherapy. J Clin Oncol 2004;22:1589-97.
82. Hanna NH, Kaiser R, Sullivan RN, et al. Nintedanib plus pemetrexed versus placebo plus pemetrexed in patients with relapsed or refractory, advanced non-small cell lung cancer (LUME-Lung 2): A randomized, double-blind, phase III trial. Lung Cancer 2016;102:65-73.
83. Jassem J, Ramlau R, Santoro A, et al. Phase III trial of pemetrexed plus best supportive care compared with best supportive care in previously treated patients with advanced malignant pleural mesothelioma. J Clin Oncol 2008;26:1698-704.
84. Karampeazis A, Voutsina A, Souglakos J, et al. Pemetrexed versus erlotinib in pretreated patients with advanced non-small cell lung cancer: a Hellenic Oncology Research Group (HORG) randomized phase 3 study. Cancer 2013;119:2754-64.
85. Karayama M, Inui N, Fujisawa T, et al. Maintenance therapy with pemetrexed and bevacizumab versus pemetrexed monotherapy after induction therapy with carboplatin, pemetrexed, and bevacizumab in patients with advanced non-squamous non small cell lung cancer. Eur J Cancer 2016;58:30-7.
86. Kim ES, Neubauer M, Cohn A, et al. Docetaxel or pemetrexed with or without cetuximab in recurrent or progressive non-small-cell lung cancer after platinum-based therapy: a phase 3, open-label, randomised trial. Lancet Oncol 2013;14:1326-36.
87. Lee DH, Lee JS, Kim SW, et al. Three-arm randomised controlled phase 2 study comparing pemetrexed and erlotinib to either pemetrexed or erlotinib alone as second-line treatment for never-smokers with non-squamous non-small cell lung cancer. Eur J Cancer 2013;49:3111-21.
88. Li N, Ou W, Yang H, et al. A randomized phase 2 trial of erlotinib versus pemetrexed as second-line therapy in the treatment of patients with advanced EGFR wild-type and EGFR FISH-positive lung adenocarcinoma. Cancer 2014;120:1379-86.
89. Paz-Ares LG, de Marinis F, Dediu M, et al. PARAMOUNT: Final overall survival results of the phase III study of maintenance pemetrexed versus placebo immediately after induction treatment with pemetrexed plus cisplatin for advanced nonsquamous non-small-cell lung cancer. J Clin Oncol 2013;31:2895-902.
90. Smit EF, Burgers SA, Biesma B, et al. Randomized phase II and pharmacogenetic study of pemetrexed compared with pemetrexed plus carboplatin in pretreated patients with advanced non-small-cell lung cancer. J Clin Oncol 2009;27:2038-45.
91. Sun JM, Lee KH, Kim SW, et al. Gefitinib versus pemetrexed as second-line treatment in patients with nonsmall cell lung cancer previously treated with platinum-based chemotherapy (KCSG-LU08-01): an open-label, phase 3 trial. Cancer 2012;118:6234-42.
92. Zhou Q, Cheng Y, Yang JJ, et al. Pemetrexed versus gefitinib as a second-line treatment in advanced nonsquamous nonsmall-cell lung cancer patients harboring wild-type EGFR (CTONG0806): a multicenter randomized trial. Ann Oncol 2014;25:2385-91.

93. Zukin M, Barrios CH, Pereira JR, et al. Randomized phase III trial of single-agent pemetrexed versus carboplatin and pemetrexed in patients with advanced non-small-cell lung cancer and Eastern Cooperative Oncology Group performance status of 2. J Clin Oncol 2013;31:2849-53.
94. Long GV, Stroyakovskiy D, Gogas H, et al. Combined BRAF and MEK inhibition versus BRAF inhibition alone in melanoma. N Engl J Med 2014;371:1877-88.
95. Chapman PB, Hauschild A, Robert C, et al. Improved survival with vemurafenib in melanoma with BRAF V600E mutation. N Engl J Med 2011;364:2507-16.
96. Larkin J, Ascierto PA, Dreno B, et al. Combined vemurafenib and cobimetinib in BRAF-mutated melanoma. N Engl J Med 2014;371:1867-76.
97. McArthur GA, Maio M, Arance A, et al. Vemurafenib in metastatic melanoma patients with brain metastases: an open-label, single-arm, phase 2, multicentre study. Ann Oncol 2017;28:634-41.
98. Robert C, Karaszewska B, Schachter J, et al. Improved overall survival in melanoma with combined dabrafenib and trametinib. N Engl J Med 2015;372:30-9.
99. Sosman JA, Kim KB, Schuchter L, et al. Survival in BRAF V600-mutant advanced melanoma treated with vemurafenib. N Engl J Med 2012;366:707-14.
100. Johnson BE, Kabbinavar F, Fehrenbacher L, et al. ATLAS: randomized, double-blind, placebo-controlled, phase IIIB trial comparing bevacizumab therapy with or without erlotinib, after completion of chemotherapy, with bevacizumab for first-line treatment of advanced non-small-cell lung cancer. J Clin Oncol 2013;31:3926-34.
101. Scagliotti GV, Krzakowski M, Szczesna A, et al. Sunitinib plus erlotinib versus placebo plus erlotinib in patients with previously treated advanced non-small-cell lung cancer: a phase III trial. J Clin Oncol 2012;30:2070-8.
102. Scagliotti G, von Pawel J, Novello S, et al. Phase III Multinational, Randomized, Double-Blind, Placebo-Controlled Study of Tivantinib (ARQ 197) Plus Erlotinib Versus Erlotinib Alone in Previously Treated Patients With Locally Advanced or Metastatic Nonsquamous Non-Small-Cell Lung Cancer. J Clin Oncol 2015;33:2667-74.
103. Andersson M, Lidbrink E, Bjerre K, et al. Phase III randomized study comparing docetaxel plus trastuzumab with vinorelbine plus trastuzumab as first-line therapy of metastatic or locally advanced human epidermal growth factor receptor 2-positive breast cancer: the HERNATA study. J Clin Oncol 2011;29:264-71.
104. Baselga J, Cortes J, Kim SB, et al. Pertuzumab plus trastuzumab plus docetaxel for metastatic breast cancer. N Engl J Med 2012;366:109-19.
105. Baselga J, Manikhas A, Cortes J, et al. Phase III trial of nonpegylated liposomal doxorubicin in combination with trastuzumab and paclitaxel in HER2-positive metastatic breast cancer. Ann Oncol 2014;25:592-8.
106. Blackwell KL, Burstein HJ, Storniolo AM, et al. Randomized study of Lapatinib alone or in combination with trastuzumab in women with ErbB2-positive, trastuzumab-refractory metastatic breast cancer. J Clin Oncol 2010;28:1124-30.
107. Chan A, Martin M, Untch M, et al. Vinorelbine plus trastuzumab combination as first-line therapy for HER 2-positive metastatic breast cancer patients: an international phase II trial. Br J Cancer 2006;95:788-93.
108. Chan A, Conte PF, Petruzelka L, et al. Phase II study of a triple combination of oral vinorelbine, capecitabine and trastuzumab as first-line treatment in HER2-positive metastatic breast cancer. Anticancer Res 2013;33:2657-64.
109. Dang C, Iyengar N, Datko F, et al. Phase II study of paclitaxel given once per week along with trastuzumab and pertuzumab in patients with human epidermal growth factor receptor 2-positive metastatic breast cancer. J Clin Oncol 2015;33:442-7.
110. Gelmon KA, Boyle FM, Kaufman B, et al. Lapatinib or Trastuzumab Plus Taxane Therapy for Human Epidermal Growth Factor Receptor 2-Positive Advanced Breast Cancer: Final Results of NCIC CTG MA.31. J Clin Oncol 2015;33:1574-83.
111. Hurvitz SA, Andre F, Jiang Z, et al. Combination of everolimus with trastuzumab plus paclitaxel as first-line treatment for patients with HER2-positive advanced breast cancer (BOLERO-1): a phase 3, randomised, double-blind, multicentre trial. Lancet Oncol 2015;16:816-29.
112. Martin M, Makhson A, Gligorov J, et al. Phase II study of bevacizumab in combination with trastuzumab and capecitabine as first-line treatment for HER-2-positive locally recurrent or metastatic breast cancer. Oncologist 2012;17:469-75.
113. Perez EA, Lopez-Vega JM, Petit T, et al. Safety and efficacy of vinorelbine in combination with pertuzumab and trastuzumab for first-line treatment of patients with HER2-positive locally advanced or metastatic breast cancer: VELVET Cohort 1 final results. Breast Cancer Res 2016;18:126.
114. Pivot X, Manikhas A, Zurawski B, et al. CEREBEL (EGF111438): A Phase III, Randomized, Open-Label Study of Lapatinib Plus Capecitabine Versus Trastuzumab Plus Capecitabine in Patients With Human Epidermal Growth Factor Receptor 2-Positive Metastatic Breast Cancer. J Clin Oncol 2015;33:1564-73.
115. Robert N, Leyland-Jones B, Asmar L, et al. Randomized phase III study of trastuzumab, paclitaxel, and carboplatin compared with trastuzumab and paclitaxel in women with HER-2-overexpressing metastatic breast cancer. J Clin Oncol 2006;24:2786-92.
116. Swain SM, Baselga J, Kim SB, et al. Pertuzumab, trastuzumab, and docetaxel in HER2-positive metastatic breast cancer. N Engl J Med 2015;372:724-34.
117. Toi M, Shao Z, Hurvitz S, et al. Efficacy and safety of everolimus in combination with trastuzumab and paclitaxel in Asian patients with HER2+ advanced breast cancer in BOLERO-1. Breast Cancer Res 2017;19:47.
118. Valero V, Forbes J, Pegram MD, et al. Multicenter phase III randomized trial comparing docetaxel and trastuzumab with docetaxel, carboplatin, and trastuzumab as first-line chemotherapy for patients with HER2-gene-amplified metastatic breast cancer (BCIRG 007 study): two highly active therapeutic regimens. J Clin Oncol 2011;29:149-56.
119. Wardley AM, Pivot X, Morales-Vasquez F, et al. Randomized phase II trial of first-line trastuzumab plus docetaxel and capecitabine compared with trastuzumab plus docetaxel in HER2-positive metastatic breast cancer. J Clin Oncol 2010;28:976-83.
120. Govindan R, Szczesna A, Ahn MJ, et al. Phase III Trial of Ipilimumab Combined With Paclitaxel and Carboplatin in Advanced Squamous Non-Small-Cell Lung Cancer. J Clin Oncol 2017;35:3449-57.
121. Hodi FS, O'Day SJ, McDermott DF, et al. Improved survival with ipilimumab in patients with metastatic melanoma. N Engl J Med 2010;363:711-23.
122. Lynch TJ, Bondarenko I, Luft A, et al. Ipilimumab in combination with paclitaxel and carboplatin as first-line treatment in stage IIIB/IV non-small-cell lung cancer: results from a randomized, double-blind, multicenter phase II study. J Clin Oncol 2012;30:2046-54.
123. Reck M, Luft A, Szczesna A, et al. Phase III Randomized Trial of Ipilimumab Plus Etoposide and Platinum Versus Placebo Plus Etoposide and Platinum in Extensive-Stage Small-Cell Lung Cancer. J Clin Oncol 2016;34:3740-8.
124. Robert C, Thomas L, Bondarenko I, et al. Ipilimumab plus dacarbazine for previously untreated metastatic melanoma. N Engl J Med 2011;364:2517-26.
125. Fassnacht M, Terzolo M, Allolio B, et al. Combination chemotherapy in advanced adrenocortical carcinoma. N Engl J Med 2012;366:2189-97.
126. Valle J, Wasan H, Palmer DH, et al. Cisplatin plus gemcitabine versus gemcitabine for biliary tract cancer. N Engl J Med 2010;362:1273-81.
127. Albain KS, Nag SM, Calderillo-Ruiz G, et al. Gemcitabine plus Paclitaxel versus Paclitaxel monotherapy in patients with metastatic breast cancer and prior anthracycline treatment. J Clin Oncol 2008;26:3950-7.
128. Baselga J, Segalla JG, Roche H, et al. Sorafenib in combination with capecitabine: an oral regimen for patients with HER2-negative locally advanced or metastatic breast cancer. J Clin Oncol 2012;30:1484-91.
129. Miller K, Wang M, Gralow J, et al. Paclitaxel plus bevacizumab versus paclitaxel alone for metastatic breast cancer. N Engl J Med 2007;357:2666-76.
130. Park YH, Jung KH, Im SA, et al. Phase III, multicenter, randomized trial of maintenance chemotherapy versus observation in patients with metastatic breast cancer after achieving disease control with six cycles of gemcitabine plus paclitaxel as first-line chemotherapy: KCSG-BR07-02. J Clin Oncol 2013;31:1732-9.
131. Rugo HS, Barry WT, Moreno-Aspitia A, et al. Randomized Phase III Trial of Paclitaxel Once Per Week Compared With Nanoparticle Albumin-Bound Nab-Paclitaxel Once Per Week or Ixabepilone With Bevacizumab As First-Line Chemotherapy for Locally Recurrent or Metastatic Breast Cancer: CALGB 40502/NCCTG N063H (Alliance). J Clin Oncol 2015;33:2361-9.
132. Sparano JA, Makhson AN, Semiglazov VF, et al. Pegylated liposomal doxorubicin plus docetaxel significantly improves time to progression without additive cardiotoxicity compared with docetaxel monotherapy in patients with advanced breast cancer previously treated with neoadjuvant-adjuvant anthracycline therapy: results from a randomized phase III study. J Clin Oncol 2009;27:4522-9.
133. Sparano JA, Vrdoljak E, Rixe O, et al. Randomized phase III trial of ixabepilone plus capecitabine versus capecitabine in patients with metastatic breast cancer previously treated with an anthracycline and a taxane. J Clin Oncol 2010;28:3256-63.
134. Stockler MR, Harvey VJ, Francis PA, et al. Capecitabine versus classical cyclophosphamide, methotrexate, and fluorouracil as first-line chemotherapy for advanced breast cancer. J Clin Oncol 2011;29:4498-504.
135. Baselga J, Campone M, Piccart M, et al. Everolimus in postmenopausal hormone-receptor-positive advanced breast cancer. N Engl J Med 2012;366:520-9.
136. Guan Z, Xu B, DeSilvio ML, et al. Randomized trial of lapatinib versus placebo added to paclitaxel in the treatment of human epidermal growth factor receptor 2-overexpressing metastatic breast cancer. J Clin Oncol 2013;31:1947-53.
137. Verma S, Miles D, Gianni L, et al. Trastuzumab emtansine for HER2-positive advanced breast cancer. N Engl J Med 2012;367:1783-91.
138. O'Shaughnessy J, Schwartzberg L, Danso MA, et al. Phase III study of iniparib plus gemcitabine and carboplatin versus gemcitabine and carboplatin in patients with metastatic triple-negative breast cancer. J Clin Oncol 2014;32:3840-7.
139. Monk BJ, Sill MW, McMeekin DS, et al. Phase III trial of four cisplatin-containing doublet combinations in stage IVB, recurrent, or persistent cervical carcinoma: a Gynecologic Oncology Group study. J Clin Oncol 2009;27:4649-55.
140. Tewari KS, Sill MW, Long HJ, 3rd, et al. Improved survival with bevacizumab in advanced cervical cancer. N Engl J Med 2014;370:734-43.

141. Douillard JY, Siena S, Cassidy J, et al. Randomized, phase III trial of panitumumab with infusional fluorouracil, leucovorin, and oxaliplatin (FOLFOX4) versus FOLFOX4 alone as first-line treatment in patients with previously untreated metastatic colorectal cancer: the PRIME study. J Clin Oncol 2010;28:4697-705.
142. Falcone A, Ricci S, Brunetti I, et al. Phase III trial of infusional fluorouracil, leucovorin, oxaliplatin, and irinotecan (FOLFOXIRI) compared with infusional fluorouracil, leucovorin, and irinotecan (FOLFIRI) as first-line treatment for metastatic colorectal cancer: the Gruppo Oncologico Nord Ovest. J Clin Oncol 2007;25:1670-6.
143. Fuchs CS, Marshall J, Mitchell E, et al. Randomized, controlled trial of irinotecan plus infusional, bolus, or oral fluoropyrimidines in first-line treatment of metastatic colorectal cancer: results from the BICC-C Study. J Clin Oncol 2007;25:4779-86.
144. Giantonio BJ, Catalano PJ, Meropol NJ, et al. Bevacizumab in combination with oxaliplatin, fluorouracil, and leucovorin (FOLFOX4) for previously treated metastatic colorectal cancer: results from the Eastern Cooperative Oncology Group Study E3200. J Clin Oncol 2007;25:1539-44.
145. Haller DG, Rothenberg ML, Wong AO, et al. Oxaliplatin plus irinotecan compared with irinotecan alone as second-line treatment after single-agent fluoropyrimidine therapy for metastatic colorectal carcinoma. J Clin Oncol 2008;26:4544-50.
146. Hecht JR, Mitchell E, Chidiac T, et al. A randomized phase IIIB trial of chemotherapy, bevacizumab, and panitumumab compared with chemotherapy and bevacizumab alone for metastatic colorectal cancer. J Clin Oncol 2009;27:672-80.
147. Hoff PM, Hochhaus A, Pestalozzi BC, et al. Cediranib plus FOLFOX/CAPOX versus placebo plus FOLFOX/CAPOX in patients with previously untreated metastatic colorectal cancer: a randomized, double-blind, phase III study (HORIZON II). J Clin Oncol 2012;30:3596-603.
148. Kim GP, Sargent DJ, Mahoney MR, et al. Phase III noninferiority trial comparing irinotecan with oxaliplatin, fluorouracil, and leucovorin in patients with advanced colorectal carcinoma previously treated with fluorouracil: N9841. J Clin Oncol 2009;27:2848-54.
149. Loupakis F, Cremolini C, Masi G, et al. Initial therapy with FOLFOXIRI and bevacizumab for metastatic colorectal cancer. N Engl J Med 2014;371:1609-18.
150. Saltz LB, Clarke S, Diaz-Rubio E, et al. Bevacizumab in combination with oxaliplatin-based chemotherapy as first-line therapy in metastatic colorectal cancer: a randomized phase III study. J Clin Oncol 2008;26:2013-9.
151. Schwartzberg LS, Rivera F, Karthaus M, et al. PEAK: a randomized, multicenter phase II study of panitumumab plus modified fluorouracil, leucovorin, and oxaliplatin (mFOLFOX6) or bevacizumab plus mFOLFOX6 in patients with previously untreated, unresectable, wild-type KRAS exon 2 metastatic colorectal cancer. J Clin Oncol 2014;32:2240-7.
152. Siu LL, Shapiro JD, Jonker DJ, et al. Phase III randomized, placebo-controlled study of cetuximab plus brivanib alaninate versus cetuximab plus placebo in patients with metastatic, chemotherapy-refractory, wild-type K-RAS colorectal carcinoma: the NCIC Clinical Trials Group and AGITG CO.20 Trial. J Clin Oncol 2013;31:2477-84.
153. Sobrero AF, Maurel J, Fehrenbacher L, et al. EPIC: phase III trial of cetuximab plus irinotecan after fluoropyrimidine and oxaliplatin failure in patients with metastatic colorectal cancer. J Clin Oncol 2008;26:2311-9.
154. Tebbutt NC, Wilson K, Gebski VJ, et al. Capecitabine, bevacizumab, and mitomycin in first-line treatment of metastatic colorectal cancer: results of the Australasian Gastrointestinal Trials Group Randomized Phase III MAX Study. J Clin Oncol 2010;28:3191-8.
155. Tol J, Koopman M, Cats A, et al. Chemotherapy, bevacizumab, and cetuximab in metastatic colorectal cancer. N Engl J Med 2009;360:563-72.
156. Van Cutsem E, Kohne CH, Hitre E, et al. Cetuximab and chemotherapy as initial treatment for metastatic colorectal cancer. N Engl J Med 2009;360:1408-17.
157. Van Cutsem E, Bajetta E, Valle J, et al. Randomized, placebo-controlled, phase III study of oxaliplatin, fluorouracil, and leucovorin with or without PTK787/ZK 222584 in patients with previously treated metastatic colorectal adenocarcinoma. J Clin Oncol 2011;29:2004-10.
158. Van Cutsem E, Tabernero J, Lakomy R, et al. Addition of aflibercept to fluorouracil, leucovorin, and irinotecan improves survival in a phase III randomized trial in patients with metastatic colorectal cancer previously treated with an oxaliplatin-based regimen. J Clin Oncol 2012;30:3499-506.
159. Peeters M, Price TJ, Cervantes A, et al. Randomized phase III study of panitumumab with fluorouracil, leucovorin, and irinotecan (FOLFIRI) compared with FOLFIRI alone as second-line treatment in patients with metastatic colorectal cancer. J Clin Oncol 2010;28:4706-13.
160. Ohtsu A, Shah MA, Van Cutsem E, et al. Bevacizumab in combination with chemotherapy as first-line therapy in advanced gastric cancer: a randomized, double-blind, placebo-controlled phase III study. J Clin Oncol 2011;29:3968-76.
161. Hecht JR, Bang YJ, Qin SK, et al. Lapatinib in Combination With Capecitabine Plus Oxaliplatin in Human Epidermal Growth Factor Receptor 2-Positive Advanced or Metastatic Gastric, Esophageal, or Gastroesophageal Adenocarcinoma: TRIO-013/LOGiC--A Randomized Phase III Trial. J Clin Oncol 2016;34:443-51.
162. Brada M, Stenning S, Gabe R, et al. Temozolomide versus procarbazine, lomustine, and vincristine in recurrent high-grade glioma. J Clin Oncol 2010;28:4601-8.
163. Chinot OL, Wick W, Mason W, et al. Bevacizumab plus radiotherapy-temozolomide for newly diagnosed glioblastoma. N Engl J Med 2014;370:709-22.
164. Gilbert MR, Dignam JJ, Armstrong TS, et al. A randomized trial of bevacizumab for newly diagnosed glioblastoma. N Engl J Med 2014;370:699-708.
165. Vermorken JB, Mesia R, Rivera F, et al. Platinum-based chemotherapy plus cetuximab in head and neck cancer. N Engl J Med 2008;359:1116-27.
166. Atkins MB, Hsu J, Lee S, et al. Phase III trial comparing concurrent biochemotherapy with cisplatin, vinblastine, dacarbazine, interleukin-2, and interferon alfa-2b with cisplatin, vinblastine, and dacarbazine alone in patients with metastatic malignant melanoma (E3695): a trial coordinated by the Eastern Cooperative Oncology Group. J Clin Oncol 2008;26:5748-54.
167. Abe T, Takeda K, Ohe Y, et al. Randomized phase III trial comparing weekly docetaxel plus cisplatin versus docetaxel monotherapy every 3 weeks in elderly patients with advanced non-small-cell lung cancer: the intergroup trial JCOG0803/WJOG4307L. J Clin Oncol 2015;33:575-81.
168. Belani CP, Yamamoto N, Bondarenko IM, et al. Randomized phase II study of pemetrexed/cisplatin with or without axitinib for non-squamous non-small-cell lung cancer. BMC Cancer 2014;14:290.
169. Crawford J, Swanson P, Schwarzenberger P, et al. A phase 2 randomized trial of paclitaxel and carboplatin with or without panitumumab for first-line treatment of advanced non-small-cell lung cancer. J Thorac Oncol 2013;8:1510-8.
170. Han JY, Park K, Kim SW, et al. First-SIGNAL: first-line single-agent iressa versus gemcitabine and cisplatin trial in never-smokers with adenocarcinoma of the lung. J Clin Oncol 2012;30:1122-8.
171. Heigener DF, Deppermann KM, Pawel JV, et al. Open, randomized, multi-center phase II study comparing efficacy and tolerability of Erlotinib vs. Carboplatin/Vinorelbin in elderly patients (>70 years of age) with untreated non-small cell lung cancer. Lung Cancer 2014;84:62-6.
172. Janne PA, Paz-Ares L, Oh Y, et al. Randomized, double-blind, phase II trial comparing gemcitabine-cisplatin plus the LTB4 antagonist LY293111 versus gemcitabine-cisplatin plus placebo in first-line non-small-cell lung cancer. J Thorac Oncol 2014;9:126-31.
173. Kubota K, Sakai H, Katakami N, et al. A randomized phase III trial of oral S-1 plus cisplatin versus docetaxel plus cisplatin in Japanese patients with advanced non-small-cell lung cancer: TCOG0701 CATS trial. Ann Oncol 2015;26:1401-8.
174. Langer CJ, Novello S, Park K, et al. Randomized, phase III trial of first-line figitumumab in combination with paclitaxel and carboplatin versus paclitaxel and carboplatin alone in patients with advanced non-small-cell lung cancer. J Clin Oncol 2014;32:2059-66.
175. Lilenbaum R, Axelrod R, Thomas S, et al. Randomized phase II trial of erlotinib or standard chemotherapy in patients with advanced non-small-cell lung cancer and a performance status of 2. J Clin Oncol 2008;26:863-9.
176. Ma X, Song Y, Zhang K, et al. Recombinant mutated human TNF in combination with chemotherapy for stage IIIB/IV non-small cell lung cancer: a randomized, phase III study. Sci Rep 2015;4:9918.
177. Mitsudomi T, Morita S, Yatabe Y, et al. Gefitinib versus cisplatin plus docetaxel in patients with non-small-cell lung cancer harbouring mutations of the epidermal growth factor receptor (WJTOG3405): an open label, randomised phase 3 trial. Lancet Oncol 2010;11:121-8.
178. Novello S, Besse B, Felip E, et al. A phase II randomized study evaluating the addition of iniparib to gemcitabine plus cisplatin as first-line therapy for metastatic non-small-cell lung cancer. Ann Oncol 2014;25:2156-62.
179. Novello S, Scagliotti GV, Sydorenko O, et al. Motesanib plus carboplatin/paclitaxel in patients with advanced squamous non-small-cell lung cancer: results from the randomized controlled MONET1 study. J Thorac Oncol 2014;9:1154-61.
180. Patel JD, Socinski MA, Garon EB, et al. PointBreak: a randomized phase III study of pemetrexed plus carboplatin and bevacizumab followed by maintenance pemetrexed and bevacizumab versus paclitaxel plus carboplatin and bevacizumab followed by maintenance bevacizumab in patients with stage IIIB or IV nonsquamous non-small-cell lung cancer. J Clin Oncol 2013;31:4349-57.
181. Paz-Ares LG, Biesma B, Heigener D, et al. Phase III, randomized, double-blind, placebo-controlled trial of gemcitabine/cisplatin alone or with sorafenib for the first-line treatment of advanced, nonsquamous non-small-cell lung cancer. J Clin Oncol 2012;30:3084-92.
182. Ramlau R, Gorbunova V, Ciuleanu TE, et al. Aflibercept and Docetaxel versus Docetaxel alone after platinum failure in patients with advanced or metastatic non-small-cell lung cancer: a randomized, controlled phase III trial. J Clin Oncol 2012;30:3640-7.
183. Reck M, von Pawel J, Zatloukal P, et al. Phase III trial of cisplatin plus gemcitabine with either placebo or bevacizumab as first-line therapy for nonsquamous non-small-cell lung cancer: AVAil. J Clin Oncol 2009;27:1227-34.
184. Rosell R, Carcereny E, Gervais R, et al. Erlotinib versus standard chemotherapy as first-line treatment for European patients with advanced EGFR mutation-positive non-small-cell lung cancer (EURTAC): a multicentre, open-label, randomised phase 3 trial. Lancet Oncol 2012;13:239-46.

185. Takeda K, Hida T, Sato T, et al. Randomized phase III trial of platinum-doublet chemotherapy followed by gefitinib compared with continued platinum-doublet chemotherapy in Japanese patients with advanced non-small-cell lung cancer: results of a west Japan thoracic oncology group trial (WJTOG0203). J Clin Oncol 2010;28:753-60.
186. Tsukada H, Yokoyama A, Goto K, et al. Randomized controlled trial comparing docetaxel-cisplatin combination with weekly docetaxel alone in elderly patients with advanced non-small-cell lung cancer: Japan Clinical Oncology Group (JCOG) 0207dagger. Jpn J Clin Oncol 2015;45:88-95.
187. Wu YL, Chu DT, Han B, et al. Phase III, randomized, open-label, first-line study in Asia of gefitinib versus carboplatin/paclitaxel in clinically selected patients with advanced non-small-cell lung cancer: evaluation of patients recruited from mainland China. Asia Pac J Clin Oncol 2012;8:232-43.
188. Wu YL, Zhou C, Liam CK, et al. First-line erlotinib versus gemcitabine/cisplatin in patients with advanced EGFR mutation-positive non-small-cell lung cancer: analyses from the phase III, randomized, open-label, ENSURE study. Ann Oncol 2015;26:1883-9.
189. Zhou C, Wu YL, Chen G, et al. Erlotinib versus chemotherapy as first-line treatment for patients with advanced EGFR mutation-positive non-small-cell lung cancer (OPTIMAL, CTONG-0802): a multicentre, open-label, randomised, phase 3 study. Lancet Oncol 2011;12:735-42.
190. Mok TS, Wu YL, Thongprasert S, et al. Gefitinib or carboplatin-paclitaxel in pulmonary adenocarcinoma. N Engl J Med 2009;361:947-57.
191. Gridelli C, Ciardiello F, Gallo C, et al. First-line erlotinib followed by second-line cisplatin-gemcitabine chemotherapy in advanced non-small-cell lung cancer: the TORCH randomized trial. J Clin Oncol 2012;30:3002-11.
192. Maemondo M, Inoue A, Kobayashi K, et al. Gefitinib or chemotherapy for non-small-cell lung cancer with mutated EGFR. N Engl J Med 2010;362:2380-8.
193. Sequist LV, Yang JC, Yamamoto N, et al. Phase III study of afatinib or cisplatin plus pemetrexed in patients with metastatic lung adenocarcinoma with EGFR mutations. J Clin Oncol 2013;31:3327-34.
194. Scagliotti GV, Parikh P, von Pawel J, et al. Phase III study comparing cisplatin plus gemcitabine with cisplatin plus pemetrexed in chemotherapy-naive patients with advanced-stage non-small-cell lung cancer. J Clin Oncol 2008;26:3543-51.
195. Scagliotti GV, Vynnychenko I, Park K, et al. International, randomized, placebo-controlled, double-blind phase III study of motesanib plus carboplatin/paclitaxel in patients with advanced nonsquamous non-small-cell lung cancer: MONET1. J Clin Oncol 2012;30:2829-36.
196. Reck M, Rodriguez-Abreu D, Robinson AG, et al. Pembrolizumab versus Chemotherapy for PD-L1-Positive Non-Small-Cell Lung Cancer. N Engl J Med 2016;375:1823-33.
197. Borad MJ, Reddy SG, Bahary N, et al. Randomized Phase II Trial of Gemcitabine Plus TH-302 Versus Gemcitabine in Patients With Advanced Pancreatic Cancer. J Clin Oncol 2015;33:1475-81.
198. Conroy T, Desseigne F, Ychou M, et al. FOLFIRINOX versus gemcitabine for metastatic pancreatic cancer. N Engl J Med 2011;364:1817-25.
199. Cunningham D, Chau I, Stocken DD, et al. Phase III randomized comparison of gemcitabine versus gemcitabine plus capecitabine in patients with advanced pancreatic cancer. J Clin Oncol 2009;27:5513-8.
200. Moore MJ, Goldstein D, Hamm J, et al. Erlotinib plus gemcitabine compared with gemcitabine alone in patients with advanced pancreatic cancer: a phase III trial of the National Cancer Institute of Canada Clinical Trials Group. J Clin Oncol 2007;25:1960-6.
201. Ueno H, Ioka T, Ikeda M, et al. Randomized phase III study of gemcitabine plus S-1, S-1 alone, or gemcitabine alone in patients with locally advanced and metastatic pancreatic cancer in Japan and Taiwan: GEST study. J Clin Oncol 2013;31:1640-8.
202. Van Cutsem E, Vervenne WL, Bennouna J, et al. Phase III trial of bevacizumab in combination with gemcitabine and erlotinib in patients with metastatic pancreatic cancer. J Clin Oncol 2009;27:2231-7.
203. Von Hoff DD, Ervin T, Arena FP, et al. Increased survival in pancreatic cancer with nab-paclitaxel plus gemcitabine. N Engl J Med 2013;369:1691-703.
204. Kelly WK, Halabi S, Carducci M, et al. Randomized, double-blind, placebo-controlled phase III trial comparing docetaxel and prednisone with or without bevacizumab in men with metastatic castration-resistant prostate cancer: CALGB 90401. J Clin Oncol 2012;30:1534-40.
205. Escudier B, Bellmunt J, Negrier S, et al. Phase III trial of bevacizumab plus interferon alfa-2a in patients with metastatic renal cell carcinoma (AVOREN): final analysis of overall survival. J Clin Oncol 2010;28:2144-50.
206. Rini BI, Halabi S, Rosenberg JE, et al. Phase III trial of bevacizumab plus interferon alfa versus interferon alfa monotherapy in patients with metastatic renal cell carcinoma: final results of CALGB 90206. J Clin Oncol 2010;28:2137-43.
207. Satouchi M, Kotani Y, Shibata T, et al. Phase III study comparing amrubicin plus cisplatin with irinotecan plus cisplatin in the treatment of extensive-disease small-cell lung cancer: JCOG 0509. J Clin Oncol 2014;32:1262-8.
208. Socinski MA, Smit EF, Lorigan P, et al. Phase III study of pemetrexed plus carboplatin compared with etoposide plus carboplatin in chemotherapy-naive patients with extensive-stage small-cell lung cancer. J Clin Oncol 2009;27:4787-92.
209. Tiseo M, Boni L, Ambrosio F, et al. Italian, Multicenter, Phase III, Randomized Study of Cisplatin Plus Etoposide With or Without Bevacizumab as First-Line Treatment in Extensive-Disease Small-Cell Lung Cancer: The GOIRC-AIFA FARM6PMFJM Trial. J Clin Oncol 2017;35:1281-7.
210. Schmid P, Adams S, Rugo HS, et al. Atezolizumab and Nab-Paclitaxel in Advanced Triple-Negative Breast Cancer. N Engl J Med 2018;379:2108-21.
211. Gandhi L, Rodriguez-Abreu D, Gadgeel S, et al. Pembrolizumab plus Chemotherapy in Metastatic Non-Small-Cell Lung Cancer. N Engl J Med 2018;378:2078-92.
212. Paz-Ares L, Luft A, Vicente D, et al. Pembrolizumab plus Chemotherapy for Squamous Non-Small-Cell Lung Cancer. N Engl J Med 2018;379:2040-51.
213. Socinski MA, Jotte RM, Cappuzzo F, et al. Atezolizumab for First-Line Treatment of Metastatic Nonsquamous NSCLC. N Engl J Med 2018;378:2288-301.
214. Hodi FS, Chesney J, Pavlick AC, et al. Combined nivolumab and ipilimumab versus ipilimumab alone in patients with advanced melanoma: 2-year overall survival outcomes in a multicentre, randomised, controlled, phase 2 trial. Lancet Oncol 2016;17:1558-68.
215. Larkin J, Chiarion-Sileni V, Gonzalez R, et al. Combined Nivolumab and Ipilimumab or Monotherapy in Untreated Melanoma. N Engl J Med 2015;373:23-34.
216. Long GV, Atkinson V, Cebon JS, et al. Standard-dose pembrolizumab in combination with reduced-dose ipilimumab for patients with advanced melanoma (KEYNOTE-029): an open-label, phase 1b trial. Lancet Oncol 2017;18:1202-10.
217. Postow MA, Chesney J, Pavlick AC, et al. Nivolumab and ipilimumab versus ipilimumab in untreated melanoma. N Engl J Med 2015;372:2006-17.
218. Tawbi HA, Forsyth PA, Algazi A, et al. Combined Nivolumab and Ipilimumab in Melanoma Metastatic to the Brain. N Engl J Med 2018;379:722-30.
219. Wolchok JD, Chiarion-Sileni V, Gonzalez R, et al. Overall Survival with Combined Nivolumab and Ipilimumab in Advanced Melanoma. N Engl J Med 2017;377:1345-56.
220. Motzer RJ, Tannir NM, McDermott DF, et al. Nivolumab plus Ipilimumab versus Sunitinib in Advanced Renal-Cell Carcinoma. N Engl J Med 2018;378:1277-90.
221. Hellmann MD, Ciuleanu TE, Pluzanski A, et al. Nivolumab plus Ipilimumab in Lung Cancer with a High Tumor Mutational Burden. N Engl J Med 2018;378:2093-104.
222. Aghajanian C, Blank SV, Goff BA, et al. OCEANS: a randomized, double-blind, placebo-controlled phase III trial of chemotherapy with or without bevacizumab in patients with platinum-sensitive recurrent epithelial ovarian, primary peritoneal, or fallopian tube cancer. J Clin Oncol 2012;30:2039-45.
223. Burger RA, Brady MF, Bookman MA, et al. Incorporation of bevacizumab in the primary treatment of ovarian cancer. N Engl J Med 2011;365:2473-83.
224. du Bois A, Herrstedt J, Hardy-Bessard AC, et al. Phase III trial of carboplatin plus paclitaxel with or without gemcitabine in first-line treatment of epithelial ovarian cancer. J Clin Oncol 2010;28:4162-9.
225. Perren TJ, Swart AM, Pfisterer J, et al. A phase 3 trial of bevacizumab in ovarian cancer. N Engl J Med 2011;365:2484-96.
226. Demetri GD, von Mehren M, Jones RL, et al. Efficacy and Safety of Trabectedin or Dacarbazine for Metastatic Liposarcoma or Leiomyosarcoma After Failure of Conventional Chemotherapy: Results of a Phase III Randomized Multicenter Clinical Trial. J Clin Oncol 2016;34:786-93.
227. Flaherty KT, Robert C, Hersey P, et al. Improved survival with MEK inhibition in BRAF-mutated melanoma. N Engl J Med 2012;367:107-14.
228. Robert C, Long GV, Brady B, et al. Nivolumab in previously untreated melanoma without BRAF mutation. N Engl J Med 2015;372:320-30.
229. Cunningham D, Humblet Y, Siena S, et al. Cetuximab monotherapy and cetuximab plus irinotecan in irinotecan-refractory metastatic colorectal cancer. N Engl J Med 2004;351:337-45.
230. Hecht JR, Mitchell E, Neubauer MA, et al. Lack of correlation between epidermal growth factor receptor status and response to Panitumumab monotherapy in metastatic colorectal cancer. Clin Cancer Res 2010;16:2205-13.
231. Muro K, Yoshino T, Doi T, et al. A phase 2 clinical trial of panitumumab monotherapy in Japanese patients with metastatic colorectal cancer. Jpn J Clin Oncol 2009;39:321-6.
232. Van Cutsem E, Peeters M, Siena S, et al. Open-label phase III trial of panitumumab plus best supportive care compared with best supportive care alone in patients with chemotherapy-refractory metastatic colorectal cancer. J Clin Oncol 2007;25:1658-64.
233. Van Cutsem E, Siena S, Humblet Y, et al. An open-label, single-arm study assessing safety and efficacy of panitumumab in patients with metastatic colorectal cancer refractory to standard chemotherapy. Ann Oncol 2008;19:92-8.
234. Hecht JR, Patnaik A, Berlin J, et al. Panitumumab monotherapy in patients with previously treated metastatic colorectal cancer. Cancer 2007;110:980-8.
235. Jonker DJ, O'Callaghan CJ, Karapetis CS, et al. Cetuximab for the treatment of colorectal cancer. N Engl J Med 2007;357:2040-8.

236. Amado RG, Wolf M, Peeters M, et al. Wild-type KRAS is required for panitumumab efficacy in patients with metastatic colorectal cancer. J Clin Oncol 2008;26:1626-34.
237. Karapetis CS, Khambata-Ford S, Jonker DJ, et al. K-ras mutations and benefit from cetuximab in advanced colorectal cancer. N Engl J Med 2008;359:1757-65.
238. Zhang W, Winder T, Ning Y, et al. A let-7 microRNA-binding site polymorphism in 3'-untranslated region of KRAS gene predicts response in wild-type KRAS patients with metastatic colorectal cancer treated with cetuximab monotherapy. Ann Oncol 2011;22:104-9.
239. Machiels JP, Subramanian S, Ruzsa A, et al. Zalutumumab plus best supportive care versus best supportive care alone in patients with recurrent or metastatic squamous-cell carcinoma of the head and neck after failure of platinum-based chemotherapy: an open-label, randomised phase 3 trial. Lancet Oncol 2011;12:333-43.
240. Vermorken JB, Trigo J, Hitt R, et al. Open-label, uncontrolled, multicenter phase II study to evaluate the efficacy and toxicity of cetuximab as a single agent in patients with recurrent and/or metastatic squamous cell carcinoma of the head and neck who failed to respond to platinum-based therapy. J Clin Oncol 2007;25:2171-7.
241. Cheng Y, Murakami H, Yang PC, et al. Randomized Phase II Trial of Gefitinib With and Without Pemetrexed as First-Line Therapy in Patients With Advanced Nonsquamous Non-Small-Cell Lung Cancer With Activating Epidermal Growth Factor Receptor Mutations. J Clin Oncol 2016;34:3258-66.
242. Douillard JY, Ostoros G, Cobo M, et al. First-line gefitinib in Caucasian EGFR mutation-positive NSCLC patients: a phase-IV, open-label, single-arm study. Br J Cancer 2014;110:55-62.
243. Goto K, Nishio M, Yamamoto N, et al. A prospective, phase II, open-label study (JO22903) of first-line erlotinib in Japanese patients with epidermal growth factor receptor (EGFR) mutation-positive advanced non-small-cell lung cancer (NSCLC). Lung Cancer 2013;82:109-14.
244. Park K, Tan EH, O'Byrne K, et al. Afatinib versus gefitinib as first-line treatment of patients with EGFR mutation-positive non-small-cell lung cancer (LUX-Lung 7): a phase 2B, open-label, randomised controlled trial. Lancet Oncol 2016;17:577-89.
245. Seto T, Kato T, Nishio M, et al. Erlotinib alone or with bevacizumab as first-line therapy in patients with advanced non-squamous non-small-cell lung cancer harbouring EGFR mutations (JO25567): an open-label, randomised, multicentre, phase 2 study. Lancet Oncol 2014;15:1236-44.
246. Soria JC, Ohe Y, Vansteenkiste J, et al. Osimertinib in Untreated EGFR-Mutated Advanced Non-Small-Cell Lung Cancer. N Engl J Med 2018;378:113-25.
247. Urata Y, Katakami N, Morita S, et al. Randomized Phase III Study Comparing Gefitinib With Erlotinib in Patients With Previously Treated Advanced Lung Adenocarcinoma: WJOG 5108L. J Clin Oncol 2016;34:3248-57.
248. Wu YL, Zhou C, Hu CP, et al. Afatinib versus cisplatin plus gemcitabine for first-line treatment of Asian patients with advanced non-small-cell lung cancer harbouring EGFR mutations (LUX-Lung 6): an open-label, randomised phase 3 trial. Lancet Oncol 2014;15:213-22.
249. Wu YL, Cheng Y, Zhou X, et al. Dacomitinib versus gefitinib as first-line treatment for patients with EGFR-mutation-positive non-small-cell lung cancer (ARCHER 1050): a randomised, open-label, phase 3 trial. Lancet Oncol 2017;18:1454-66.
250. Aerts JG, Codrington H, Lankheet NA, et al. A randomized phase II study comparing erlotinib versus erlotinib with alternating chemotherapy in relapsed non-small-cell lung cancer patients: the NVALT-10 study. Ann Oncol 2013;24:2860-5.
251. Ahn JS, Lee KH, Sun JM, et al. A randomized, phase II study of vandetanib maintenance for advanced or metastatic non-small-cell lung cancer following first-line platinum-doublet chemotherapy. Lung Cancer 2013;82:455-60.
252. Cappuzzo F, Ciuleanu T, Stelmakh L, et al. Erlotinib as maintenance treatment in advanced non-small-cell lung cancer: a multicentre, randomised, placebo-controlled phase 3 study. Lancet Oncol 2010;11:521-9.
253. Chen YM, Fan WC, Tsai CM, et al. A phase II randomized trial of gefitinib alone or with tegafur/uracil treatment in patients with pulmonary adenocarcinoma who had failed previous chemotherapy. J Thorac Oncol 2011;6:1110-6.
254. Bepler G, Williams C, Schell MJ, et al. Randomized international phase III trial of ERCC1 and RRM1 expression-based chemotherapy versus gemcitabine/carboplatin in advanced non-small-cell lung cancer. J Clin Oncol 2013;31:2404-12.
255. Cicenas S, Geater SL, Petrov P, et al. Maintenance erlotinib versus erlotinib at disease progression in patients with advanced non-small-cell lung cancer who have not progressed following platinum-based chemotherapy (IUNO study). Lung Cancer 2016;102:30-7.
256. Ciuleanu T, Stelmakh L, Cicenas S, et al. Efficacy and safety of erlotinib versus chemotherapy in second-line treatment of patients with advanced, non-small-cell lung cancer with poor prognosis (TITAN): a randomised multicentre, open-label, phase 3 study. Lancet Oncol 2012;13:300-8.
257. Crino L, Cappuzzo F, Zatloukal P, et al. Gefitinib versus vinorelbine in chemotherapy-naive elderly patients with advanced non-small-cell lung cancer (INVITE): a randomized, phase II study. J Clin Oncol 2008;26:4253-60.
258. Ellis PM, Shepherd FA, Millward M, et al. Dacomitinib compared with placebo in pretreated patients with advanced or metastatic non-small-cell lung cancer (NCIC CTG BR.26): a double-blind, randomised, phase 3 trial. Lancet Oncol 2014;15:1379-88.
259. Fukuoka M, Yano S, Giaccone G, et al. Multi-institutional randomized phase II trial of gefitinib for previously treated patients with advanced non-small-cell lung cancer (The IDEAL 1 Trial) [corrected]. J Clin Oncol 2003;21:2237-46.
260. Gaafar RM, Surmont VF, Scagliotti GV, et al. A double-blind, randomised, placebo-controlled phase III intergroup study of gefitinib in patients with advanced NSCLC, non-progressing after first line platinum-based chemotherapy (EORTC 08021/ILCP 01/03). Eur J Cancer 2011;47:2331-40.
261. Gemma A, Kudoh S, Ando M, et al. Final safety and efficacy of erlotinib in the phase 4 POLARSTAR surveillance study of 10 708 Japanese patients with non-small-cell lung cancer. Cancer Sci 2014;105:1584-90.
262. Goss G, Ferry D, Wierzbicki R, et al. Randomized phase II study of gefitinib compared with placebo in chemotherapy-naive patients with advanced non-small-cell lung cancer and poor performance status. J Clin Oncol 2009;27:2253-60.
263. Groen HJ, Socinski MA, Grossi F, et al. A randomized, double-blind, phase II study of erlotinib with or without sunitinib for the second-line treatment of metastatic non-small-cell lung cancer (NSCLC). Ann Oncol 2013;24:2382-9.
264. Han JY, Lee SH, Yoo NJ, et al. A randomized phase II study of gefitinib plus simvastatin versus gefitinib alone in previously treated patients with advanced non-small cell lung cancer. Clin Cancer Res 2011;17:1553-60.
265. Herbst RS, Ansari R, Bustin F, et al. Efficacy of bevacizumab plus erlotinib versus erlotinib alone in advanced non-small-cell lung cancer after failure of standard first-line chemotherapy (BeTa): a double-blind, placebo-controlled, phase 3 trial. Lancet 2011;377:1846-54.
266. Jackman DM, Yeap BY, Lindeman NI, et al. Phase II clinical trial of chemotherapy-naive patients > or = 70 years of age treated with erlotinib for advanced non-small-cell lung cancer. J Clin Oncol 2007;25:760-6.
267. Janne PA, Wang X, Socinski MA, et al. Randomized phase II trial of erlotinib alone or with carboplatin and paclitaxel in patients who were never or light former smokers with advanced lung adenocarcinoma: CALGB 30406 trial. J Clin Oncol 2012;30:2063-9.
268. Kawaguchi T, Ando M, Asami K, et al. Randomized phase III trial of erlotinib versus docetaxel as second- or third-line therapy in patients with advanced non-small-cell lung cancer: Docetaxel and Erlotinib Lung Cancer Trial (DELTA). J Clin Oncol 2014;32:1902-8.
269. Kim ES, Hirsh V, Mok T, et al. Gefitinib versus docetaxel in previously treated non-small-cell lung cancer (INTEREST): a randomised phase III trial. Lancet 2008;372:1809-18.
270. Kim HR, Jang JS, Sun JM, et al. A randomized, phase II study of gefitinib alone versus nimotuzumab plus gefitinib after platinum-based chemotherapy in advanced non-small cell lung cancer (KCSG LU12-01). Oncotarget 2017;8:15943-51.
271. Lee JS, Hirsh V, Park K, et al. Vandetanib Versus placebo in patients with advanced non-small-cell lung cancer after prior therapy with an epidermal growth factor receptor tyrosine kinase inhibitor: a randomized, double-blind phase III trial (ZEPHYR). J Clin Oncol 2012;30:1114-21.
272. Lee SM, Khan I, Upadhyay S, et al. First-line erlotinib in patients with advanced non-small-cell lung cancer unsuitable for chemotherapy (TOPICAL): a double-blind, placebo-controlled, phase 3 trial. Lancet Oncol 2012;13:1161-70.
273. Maruyama R, Nishiwaki Y, Tamura T, et al. Phase III study, V-15-32, of gefitinib versus docetaxel in previously treated Japanese patients with non-small-cell lung cancer. J Clin Oncol 2008;26:4244-52.
274. Mok T, Wu YL, Au JS, et al. Efficacy and safety of erlotinib in 1242 East/South-East Asian patients with advanced non-small cell lung cancer. J Thorac Oncol 2010;5:1609-15.
275. Mok TS, Geater SL, Su WC, et al. A Randomized Phase 2 Study Comparing the Combination of Ficlatuzumab and Gefitinib with Gefitinib Alone in Asian Patients with Advanced Stage Pulmonary Adenocarcinoma. J Thorac Oncol 2016;11:1736-44.
276. Natale RB, Bodkin D, Govindan R, et al. Vandetanib versus gefitinib in patients with advanced non-small-cell lung cancer: results from a two-part, double-blind, randomized phase ii study. J Clin Oncol 2009;27:2523-9.
277. Natale RB, Thongprasert S, Greco FA, et al. Phase III trial of vandetanib compared with erlotinib in patients with previously treated advanced non-small-cell lung cancer. J Clin Oncol 2011;29:1059-66.
278. Perng RP, Yang CH, Chen YM, et al. High efficacy of erlotinib in Taiwanese NSCLC patients in an expanded access program study previously treated with chemotherapy. Lung Cancer 2008;62:78-84.
279. Perol M, Chouaid C, Perol D, et al. Randomized, phase III study of gemcitabine or erlotinib maintenance therapy versus observation, with predefined second-line treatment, after cisplatin-gemcitabine induction chemotherapy in advanced non-small-cell lung cancer. J Clin Oncol 2012;30:3516-24.
280. Ramalingam SS, Spigel DR, Chen D, et al. Randomized phase II study of erlotinib in combination with placebo or R1507, a monoclonal antibody to insulin-like growth factor-1 receptor, for advanced-stage non-small-cell lung cancer. J Clin Oncol 2011;29:4574-80.
281. Ramalingam SS, Blackhall F, Krzakowski M, et al. Randomized phase II study of dacomitinib (PF-00299804), an irreversible pan-human epidermal growth factor receptor inhibitor, versus erlotinib in patients with advanced non-small-cell lung cancer. J Clin Oncol 2012;30:3337-44.
282. Ramalingam SS, Janne PA, Mok T, et al. Dacomitinib versus erlotinib in patients with advanced-stage, previously treated non-small-cell lung cancer (ARCHER 1009): a randomised, double-blind, phase 3 trial. Lancet Oncol 2014;15:1369-78.
283. Reck M, Buchholz E, Romer KS, Krutzfeldt K, Gatzemeier U, Manegold C. Gefitinib monotherapy in chemotherapy-naive patients with inoperable stage III/IV non-small-cell lung cancer. Clin Lung Cancer 2006;7:406-11.
284. Reck M, van Zandwijk N, Gridelli C, et al. Erlotinib in advanced non-small cell lung cancer: efficacy and safety findings of the global phase IV Tarceva Lung Cancer Survival Treatment study. J Thorac Oncol 2010;5:1616-22.
285. Scagliotti GV, Bondarenko I, Blackhall F, et al. Randomized, phase III trial of figitumumab in combination with erlotinib versus erlotinib alone in patients with nonadenocarcinoma nonsmall-cell lung cancer. Ann Oncol 2015;26:497-504.

286. Schneider CP, Heigener D, Schott-von-Romer K, et al. Epidermal growth factor receptor-related tumor markers and clinical outcomes with erlotinib in non-small cell lung cancer: an analysis of patients from german centers in the TRUST study. J Thorac Oncol 2008;3:1446-53.
287. Sequist LV, von Pawel J, Garmey EG, et al. Randomized phase II study of erlotinib plus tivantinib versus erlotinib plus placebo in previously treated non-small-cell lung cancer. J Clin Oncol 2011;29:3307-15.
288. Shepherd FA, Rodrigues Pereira J, Ciuleanu T, et al. Erlotinib in previously treated non-small-cell lung cancer. N Engl J Med 2005;353:123-32.
289. Simon GR, Ruckdeschel JC, Williams C, et al. Gefitinib (ZD1839) in previously treated advanced non-small-cell lung cancer: experience from a single institution. Cancer Control 2003;10:388-95.
290. Soria JC, Felip E, Cobo M, et al. Afatinib versus erlotinib as second-line treatment of patients with advanced squamous cell carcinoma of the lung (LUX-Lung 8): an open-label randomised controlled phase 3 trial. Lancet Oncol 2015;16:897-907.
291. Spigel DR, Burris HA, 3rd, Greco FA, et al. Randomized, double-blind, placebo-controlled, phase II trial of sorafenib and erlotinib or erlotinib alone in previously treated advanced non-small-cell lung cancer. J Clin Oncol 2011;29:2582-9.
292. Stinchcombe TE, Peterman AH, Lee CB, et al. A randomized phase II trial of first-line treatment with gemcitabine, erlotinib, or gemcitabine and erlotinib in elderly patients (age >/=70 years) with stage IIIB/IV non-small cell lung cancer. J Thorac Oncol 2011;6:1569-77.
293. Thatcher N, Chang A, Parikh P, et al. Gefitinib plus best supportive care in previously treated patients with refractory advanced non-small-cell lung cancer: results from a randomised, placebo-controlled, multicentre study (Iressa Survival Evaluation in Lung Cancer). Lancet 2005;366:1527-37.
294. Tiseo M, Gridelli C, Cascinu S, et al. An expanded access program of erlotinib (Tarceva) in patients with advanced non-small cell lung cancer (NSCLC): data report from Italy. Lung Cancer 2009;64:199-206.
295. Uhm JE, Park BB, Ahn MJ, et al. Erlotinib monotherapy for stage IIIB/IV non-small cell lung cancer: a multicenter trial by the Korean Cancer Study Group. J Thorac Oncol 2009;4:1136-43.
296. Van Meerbeeck J, Galdermans D, Bustin F, De Vos L, Lechat I, Abraham I. Survival outcomes in patients with advanced non-small cell lung cancer treated with erlotinib: expanded access programme data from Belgium (the TRUST study). Eur J Cancer Care (Engl) 2014;23:370-9.
297. West HL, Franklin WA, McCoy J, et al. Gefitinib therapy in advanced bronchioloalveolar carcinoma: Southwest Oncology Group Study S0126. J Clin Oncol 2006;24:1807-13.
298. Witta SE, Jotte RM, Konduri K, et al. Randomized phase II trial of erlotinib with and without entinostat in patients with advanced non-small-cell lung cancer who progressed on prior chemotherapy. J Clin Oncol 2012;30:2248-55.
299. Zhang L, Ma S, Song X, et al. Gefitinib versus placebo as maintenance therapy in patients with locally advanced or metastatic non-small-cell lung cancer (INFORM; C-TONG 0804): a multicentre, double-blind randomised phase 3 trial. Lancet Oncol 2012;13:466-75.
300. Bell DW, Lynch TJ, Haserlat SM, et al. Epidermal growth factor receptor mutations and gene amplification in non-small-cell lung cancer: molecular analysis of the IDEAL/INTACT gefitinib trials. J Clin Oncol 2005;23:8081-92.
301. Choi DR, Lee DH, Choi CM, Kim SW, Suh C, Lee JS. Erlotinib in first-line therapy for non-small cell lung cancer: a prospective phase II study. Anticancer Res 2011;31:3457-62.
302. Douillard JY, Shepherd FA, Hirsh V, et al. Molecular predictors of outcome with gefitinib and docetaxel in previously treated non-small-cell lung cancer: data from the randomized phase III INTEREST trial. J Clin Oncol 2010;28:744-52.
303. Garassino MC, Martelli O, Broggini M, et al. Erlotinib versus docetaxel as second-line treatment of patients with advanced non-small-cell lung cancer and wild-type EGFR tumours (TAILOR): a randomised controlled trial. Lancet Oncol 2013;14:981-8.
304. Han SW, Kim TY, Hwang PG, et al. Predictive and prognostic impact of epidermal growth factor receptor mutation in non-small-cell lung cancer patients treated with gefitinib. J Clin Oncol 2005;23:2493-501.
305. Yoshioka H, Azuma K, Yamamoto N, et al. A randomized, double-blind, placebo-controlled, phase III trial of erlotinib with or without a c-Met inhibitor tivantinib (ARQ 197) in Asian patients with previously treated stage IIIB/IV nonsquamous nonsmall-cell lung cancer harboring wild-type epidermal growth factor receptor (ATTENTION study). Ann Oncol 2015;26:2066-72.
306. Mayer RJ, Van Cutsem E, Falcone A, et al. Randomized trial of TAS-102 for refractory metastatic colorectal cancer. N Engl J Med 2015;372:1909-19.
307. Baselga J, Im SA, Iwata H, et al. Buparlisib plus fulvestrant versus placebo plus fulvestrant in postmenopausal, hormone receptor-positive, HER2-negative, advanced breast cancer (BELLE-2): a randomised, double-blind, placebo-controlled, phase 3 trial. Lancet Oncol 2017;18:904-16.
308. Chia S, Gradishar W, Mauriac L, et al. Double-blind, randomized placebo controlled trial of fulvestrant compared with exemestane after prior nonsteroidal aromatase inhibitor therapy in postmenopausal women with hormone receptor-positive, advanced breast cancer: results from EFECT. J Clin Oncol 2008;26:1664-70.
309. Cristofanilli M, Turner NC, Bondarenko I, et al. Fulvestrant plus palbociclib versus fulvestrant plus placebo for treatment of hormone-receptor-positive, HER2-negative metastatic breast cancer that progressed on previous endocrine therapy (PALOMA-3): final analysis of the multicentre, double-blind, phase 3 randomised controlled trial. Lancet Oncol 2016;17:425-39.
310. Di Leo A, Jerusalem G, Petruzelka L, et al. Results of the CONFIRM phase III trial comparing fulvestrant 250 mg with fulvestrant 500 mg in postmenopausal women with estrogen receptor-positive advanced breast cancer. J Clin Oncol 2010;28:4594-600.
311. Di Leo A, Johnston S, Lee KS, et al. Buparlisib plus fulvestrant in postmenopausal women with hormone-receptor-positive, HER2-negative, advanced breast cancer progressing on or after mTOR inhibition (BELLE-3): a randomised, double-blind, placebo-controlled, phase 3 trial. Lancet Oncol 2018;19:87-100.
312. Howell A, Robertson JF, Abram P, et al. Comparison of fulvestrant versus tamoxifen for the treatment of advanced breast cancer in postmenopausal women previously untreated with endocrine therapy: a multinational, double-blind, randomized trial. J Clin Oncol 2004;22:1605-13.
313. Jeselsohn R, Barry WT, Migliaccio I, et al. TransCONFIRM: Identification of a Genetic Signature of Response to Fulvestrant in Advanced Hormone Receptor-Positive Breast Cancer. Clin Cancer Res 2016;22:5755-64.
314. Johnston SR, Kilburn LS, Ellis P, et al. Fulvestrant plus anastrozole or placebo versus exemestane alone after progression on non-steroidal aromatase inhibitors in postmenopausal patients with hormone-receptor-positive locally advanced or metastatic breast cancer (SoFEA): a composite, multicentre, phase 3 randomised trial. Lancet Oncol 2013;14:989-98.
315. Krop IE, Mayer IA, Ganju V, et al. Pictilisib for oestrogen receptor-positive, aromatase inhibitor-resistant, advanced or metastatic breast cancer (FERGI): a randomised, double-blind, placebo-controlled, phase 2 trial. Lancet Oncol 2016;17:811-21.
316. Ohno S, Rai Y, Iwata H, et al. Three dose regimens of fulvestrant in postmenopausal Japanese women with advanced breast cancer: results from a double-blind, phase II comparative study (FINDER1). Ann Oncol 2010;21:2342-7.
317. Perey L, Paridaens R, Hawle H, et al. Clinical benefit of fulvestrant in postmenopausal women with advanced breast cancer and primary or acquired resistance to aromatase inhibitors: final results of phase II Swiss Group for Clinical Cancer Research Trial (SAKK 21/00). Ann Oncol 2007;18:64-9.
318. Pritchard KI, Rolski J, Papai Z, et al. Results of a phase II study comparing three dosing regimens of fulvestrant in postmenopausal women with advanced breast cancer (FINDER2). Breast Cancer Res Treat 2010;123:453-61.
319. Robertson JFR, Bondarenko IM, Trishkina E, et al. Fulvestrant 500 mg versus anastrozole 1 mg for hormone receptor-positive advanced breast cancer (FALCON): an international, randomised, double-blind, phase 3 trial. Lancet 2016;388:2997-3005.
320. Slamon DJ, Neven P, Chia S, et al. Phase III Randomized Study of Ribociclib and Fulvestrant in Hormone Receptor-Positive, Human Epidermal Growth Factor Receptor 2-Negative Advanced Breast Cancer: MONALEESA-3. J Clin Oncol 2018;36:2465-72.
321. Sledge GW, Jr., Toi M, Neven P, et al. MONARCH 2: Abemaciclib in Combination With Fulvestrant in Women With HR+/HER2- Advanced Breast Cancer Who Had Progressed While Receiving Endocrine Therapy. J Clin Oncol 2017;35:2875-84.
322. Turner NC, Ro J, Andre F, et al. Palbociclib in Hormone-Receptor-Positive Advanced Breast Cancer. N Engl J Med 2015;373:209-19.
323. Zhang Q, Shao Z, Shen K, et al. Fulvestrant 500 mg vs 250 mg in postmenopausal women with estrogen receptor-positive advanced breast cancer: a randomized, double-blind registrational trial in China. Oncotarget 2016;7:57301-9.
324. Brodowicz T, Krzakowski M, Zwitter M, et al. Cisplatin and gemcitabine first-line chemotherapy followed by maintenance gemcitabine or best supportive care in advanced non-small cell lung cancer: a phase III trial. Lung Cancer 2006;52:155-63.
325. Sederholm C, Hillerdal G, Lamberg K, et al. Phase III trial of gemcitabine plus carboplatin versus single-agent gemcitabine in the treatment of locally advanced or metastatic non-small-cell lung cancer: the Swedish Lung Cancer Study Group. J Clin Oncol 2005;23:8380-8.
326. Berlin JD, Catalano P, Thomas JP, Kugler JW, Haller DG, Benson AB, 3rd. Phase III study of gemcitabine in combination with fluorouracil versus gemcitabine alone in patients with advanced pancreatic carcinoma: Eastern Cooperative Oncology Group Trial E2297. J Clin Oncol 2002;20:3270-5.
327. Colucci G, Labianca R, Di Costanzo F, et al. Randomized phase III trial of gemcitabine plus cisplatin compared with single-agent gemcitabine as first-line treatment of patients with advanced pancreatic cancer: the GIP-1 study. J Clin Oncol 2010;28:1645-51.
328. Heinemann V, Quietzsch D, Gieseler F, et al. Randomized phase III trial of gemcitabine plus cisplatin compared with gemcitabine alone in advanced pancreatic cancer. J Clin Oncol 2006;24:3946-52.
329. Lee HS, Chung MJ, Park JY, et al. A randomized, multicenter, phase III study of gemcitabine combined with capecitabine versus gemcitabine alone as first-line chemotherapy for advanced pancreatic cancer in South Korea. Medicine (Baltimore) 2017;96:e5702.
330. Middleton G, Palmer DH, Greenhalf W, et al. Vandetanib plus gemcitabine versus placebo plus gemcitabine in locally advanced or metastatic pancreatic carcinoma (ViP): a prospective, randomised, double-blind, multicentre phase 2 trial. Lancet Oncol 2017;18:486-99.
331. Poplin E, Feng Y, Berlin J, et al. Phase III, randomized study of gemcitabine and oxaliplatin versus gemcitabine (fixed-dose rate infusion) compared with gemcitabine (30-minute infusion) in patients with pancreatic carcinoma E6201: a trial of the Eastern Cooperative Oncology Group. J Clin Oncol 2009;27:3778-85.

332. Rocha Lima CM, Green MR, Rotche R, et al. Irinotecan plus gemcitabine results in no survival advantage compared with gemcitabine monotherapy in patients with locally advanced or metastatic pancreatic cancer despite increased tumor response rate. J Clin Oncol 2004;22:3776-83.
333. Nishida T, Shirao K, Sawaki A, et al. Efficacy and safety profile of imatinib mesylate (ST1571) in Japanese patients with advanced gastrointestinal stromal tumors: a phase II study (STI571B1202). Int J Clin Oncol 2008;13:244-51.
334. Casali PG, Zalcberg J, Le Cesne A, et al. Ten-Year Progression-Free and Overall Survival in Patients With Unresectable or Metastatic GI Stromal Tumors: Long-Term Analysis of the European Organisation for Research and Treatment of Cancer, Italian Sarcoma Group, and Australasian Gastrointestinal Trials Group Intergroup Phase III Randomized Trial on Imatinib at Two Dose Levels. J Clin Oncol 2017;35:1713-20.
335. Blanke CD, Rankin C, Demetri GD, et al. Phase III randomized, intergroup trial assessing imatinib mesylate at two dose levels in patients with unresectable or metastatic gastrointestinal stromal tumors expressing the kit receptor tyrosine kinase: S0033. J Clin Oncol 2008;26:626-32.
336. Demetri GD, Reichardt P, Kang YK, et al. Efficacy and safety of regorafenib for advanced gastrointestinal stromal tumours after failure of imatinib and sunitinib (GRID): an international, multicentre, randomised, placebo-controlled, phase 3 trial. Lancet 2013;381:295-302.
337. Demetri GD, van Oosterom AT, Garrett CR, et al. Efficacy and safety of sunitinib in patients with advanced gastrointestinal stromal tumour after failure of imatinib: a randomised controlled trial. Lancet 2006;368:1329-38.
338. Komatsu Y, Ohki E, Ueno N, et al. Safety, efficacy and prognostic analyses of sunitinib in the post-marketing surveillance study of Japanese patients with gastrointestinal stromal tumor. Jpn J Clin Oncol 2015;45:1016-22.
339. Reichardt P, Kang YK, Rutkowski P, et al. Clinical outcomes of patients with advanced gastrointestinal stromal tumors: safety and efficacy in a worldwide treatment-use trial of sunitinib. Cancer 2015;121:1405-13.
340. Hurvitz SA, Dirix L, Kocsis J, et al. Phase II randomized study of trastuzumab emtansine versus trastuzumab plus docetaxel in patients with human epidermal growth factor receptor 2-positive metastatic breast cancer. J Clin Oncol 2013;31:1157-63.
341. Krop IE, Kim SB, Gonzalez-Martin A, et al. Trastuzumab emtansine versus treatment of physician's choice for pretreated HER2-positive advanced breast cancer (TH3RESA): a randomised, open-label, phase 3 trial. Lancet Oncol 2014;15:689-99.
342. Inoue K, Nakagami K, Mizutani M, et al. Randomized phase III trial of trastuzumab monotherapy followed by trastuzumab plus docetaxel versus trastuzumab plus docetaxel as first-line therapy in patients with HER2-positive metastatic breast cancer: the JO17360 Trial Group. Breast Cancer Res Treat 2010;119:127-36.
343. Vogel CL, Cobleigh MA, Tripathy D, et al. Efficacy and safety of trastuzumab as a single agent in first-line treatment of HER2-overexpressing metastatic breast cancer. J Clin Oncol 2002;20:719-26.
344. Creagan ET, Twito DI, Johansson SL, et al. A randomized prospective assessment of recombinant leukocyte A human interferon with or without aspirin in advanced renal adenocarcinoma. J Clin Oncol 1991;9:2104-9.
345. Escudier B, Szczylik C, Hutson TE, et al. Randomized phase II trial of first-line treatment with sorafenib versus interferon Alfa-2a in patients with metastatic renal cell carcinoma. J Clin Oncol 2009;27:1280-9.
346. Gore ME, Griffin CL, Hancock B, et al. Interferon alfa-2a versus combination therapy with interferon alfa-2a, interleukin-2, and fluorouracil in patients with untreated metastatic renal cell carcinoma (MRC RE04/EORTC GU 30012): an open-label randomised trial. Lancet 2010;375:641-8.
347. Hudes G, Carducci M, Tomczak P, et al. Temsirolimus, interferon alfa, or both for advanced renal-cell carcinoma. N Engl J Med 2007;356:2271-81.
348. Motzer RJ, Murphy BA, Bacik J, et al. Phase III trial of interferon alfa-2a with or without 13-cis-retinoic acid for patients with advanced renal cell carcinoma. J Clin Oncol 2000;18:2972-80.
349. Motzer RJ, Hutson TE, Tomczak P, et al. Sunitinib versus interferon alfa in metastatic renal-cell carcinoma. N Engl J Med 2007;356:115-24.
350. Rini BI, Halabi S, Rosenberg JE, et al. Bevacizumab plus interferon alfa compared with interferon alfa monotherapy in patients with metastatic renal cell carcinoma: CALGB 90206. J Clin Oncol 2008;26:5422-8.
351. Bang YJ, Cho JY, Kim YH, et al. Efficacy of Sequential Ipilimumab Monotherapy versus Best Supportive Care for Unresectable Locally Advanced/Metastatic Gastric or Gastroesophageal Junction Cancer. Clin Cancer Res 2017;23:5671-8.
352. Robert C, Schachter J, Long GV, et al. Pembrolizumab versus Ipilimumab in Advanced Melanoma. N Engl J Med 2015;372:2521-32.
353. Chiarion Sileni V, Pigozzo J, Ascierto PA, et al. Efficacy and safety of ipilimumab in elderly patients with pretreated advanced melanoma treated at Italian centres through the expanded access programme. J Exp Clin Cancer Res 2014;33:30.
354. Zimmer L, Vaubel J, Mohr P, et al. Phase II DeCOG-study of ipilimumab in pretreated and treatment-naive patients with metastatic uveal melanoma. PLoS One 2015;10:e0118564.
355. Beer TM, Kwon ED, Drake CG, et al. Randomized, Double-Blind, Phase III Trial of Ipilimumab Versus Placebo in Asymptomatic or Minimally Symptomatic Patients With Metastatic Chemotherapy-Naive Castration-Resistant Prostate Cancer. J Clin Oncol 2017;35:40-7.
356. Kwon ED, Drake CG, Scher HI, et al. Ipilimumab versus placebo after radiotherapy in patients with metastatic castration-resistant prostate cancer that had progressed after docetaxel chemotherapy (CA184-043): a multicentre, randomised, double-blind, phase 3 trial. Lancet Oncol 2014;15:700-12.
357. Doi T, Muro K, Boku N, et al. Multicenter phase II study of everolimus in patients with previously treated metastatic gastric cancer. J Clin Oncol 2010;28:1904-10.
358. Ohtsu A, Ajani JA, Bai YX, et al. Everolimus for previously treated advanced gastric cancer: results of the randomized, double-blind, phase III GRANITE-1 study. J Clin Oncol 2013;31:3935-43.
359. Yao JC, Pavel M, Lombard-Bohas C, et al. Everolimus for the Treatment of Advanced Pancreatic Neuroendocrine Tumors: Overall Survival and Circulating Biomarkers From the Randomized, Phase III RADIANT-3 Study. J Clin Oncol 2016;34:3906-13.
360. Buzzoni R, Carnaghi C, Strosberg J, et al. Impact of prior therapies on everolimus activity: an exploratory analysis of RADIANT-4. Onco Targets Ther 2017;10:5013-30.
361. Zhu AX, Kudo M, Assenat E, et al. Effect of everolimus on survival in advanced hepatocellular carcinoma after failure of sorafenib: the EVOLVE-1 randomized clinical trial. JAMA 2014;312:57-67.
362. Ou SH, Moon J, Garland LL, et al. SWOG S0722: phase II study of mTOR inhibitor everolimus (RAD001) in advanced malignant pleural mesothelioma (MPM). J Thorac Oncol 2015;10:387-91.
363. Kulke MH, Ruszniewski P, Van Cutsem E, et al. A randomized, open-label, phase 2 study of everolimus in combination with pasireotide LAR or everolimus alone in advanced, well-differentiated, progressive pancreatic neuroendocrine tumors: COOPERATE-2 trial. Ann Oncol 2017;28:1309-15.
364. Choueiri TK, Escudier B, Powles T, et al. Cabozantinib versus Everolimus in Advanced Renal-Cell Carcinoma. N Engl J Med 2015;373:1814-23.
365. Escudier B, Molinie V, Bracarda S, et al. Open-label phase 2 trial of first-line everolimus monotherapy in patients with papillary metastatic renal cell carcinoma: RAPTOR final analysis. Eur J Cancer 2016;69:226-35.
366. Hutson TE, Escudier B, Esteban E, et al. Randomized phase III trial of temsirolimus versus sorafenib as second-line therapy after sunitinib in patients with metastatic renal cell carcinoma. J Clin Oncol 2014;32:760-7.
367. Motzer RJ, Barrios CH, Kim TM, et al. Phase II randomized trial comparing sequential first-line everolimus and second-line sunitinib versus first-line sunitinib and second-line everolimus in patients with metastatic renal cell carcinoma. J Clin Oncol 2014;32:2765-72.
368. Motzer RJ, Escudier B, McDermott DF, et al. Nivolumab versus Everolimus in Advanced Renal-Cell Carcinoma. N Engl J Med 2015;373:1803-13.
369. Demetri GD, Chawla SP, Ray-Coquard I, et al. Results of an international randomized phase III trial of the mammalian target of rapamycin inhibitor ridaforolimus versus placebo to control metastatic sarcomas in patients after benefit from prior chemotherapy. J Clin Oncol 2013;31:2485-92.
370. Bellmunt J, de Wit R, Vaughn DJ, et al. Pembrolizumab as Second-Line Therapy for Advanced Urothelial Carcinoma. N Engl J Med 2017;376:1015-26.
371. Overman MJ, McDermott R, Leach JL, et al. Nivolumab in patients with metastatic DNA mismatch repair-deficient or microsatellite instability-high colorectal cancer (CheckMate 142): an open-label, multicentre, phase 2 study. Lancet Oncol 2017;18:1182-91.
372. Shitara K, Ozguroglu M, Bang YJ, et al. Pembrolizumab versus paclitaxel for previously treated, advanced gastric or gastro-oesophageal junction cancer (KEYNOTE-061): a randomised, open-label, controlled, phase 3 trial. Lancet 2018;392:123-33.
373. Bauml J, Seiwert TY, Pfister DG, et al. Pembrolizumab for Platinum- and Cetuximab-Refractory Head and Neck Cancer: Results From a Single-Arm, Phase II Study. J Clin Oncol 2017;35:1542-9.
374. Chow LQM, Haddad R, Gupta S, et al. Antitumor Activity of Pembrolizumab in Biomarker-Unselected Patients With Recurrent and/or Metastatic Head and Neck Squamous Cell Carcinoma: Results From the Phase Ib KEYNOTE-012 Expansion Cohort. J Clin Oncol 2016;34:3838-45.
375. Ferris RL, Blumenschein G, Jr., Fayette J, et al. Nivolumab for Recurrent Squamous-Cell Carcinoma of the Head and Neck. N Engl J Med 2016;375:1856-67.
376. Younes A, Santoro A, Shipp M, et al. Nivolumab for classical Hodgkin's lymphoma after failure of both autologous stem-cell transplantation and brentuximab vedotin: a multicentre, multicohort, single-arm phase 2 trial. Lancet Oncol 2016;17:1283-94.
377. Tumeh PC, Hellmann MD, Hamid O, et al. Liver Metastasis and Treatment Outcome with Anti-PD-1 Monoclonal Antibody in Patients with Melanoma and NSCLC. Cancer Immunol Res 2017;5:417-24.
378. Ribas A, Puzanov I, Dummer R, et al. Pembrolizumab versus investigator-choice chemotherapy for ipilimumab-refractory melanoma (KEYNOTE-002): a randomised, controlled, phase 2 trial. Lancet Oncol 2015;16:908-18.
379. Ribas A, Hamid O, Daud A, et al. Association of Pembrolizumab With Tumor Response and Survival Among Patients With Advanced Melanoma. JAMA 2016;315:1600-9.
380. Robert C, Ribas A, Wolchok JD, et al. Anti-programmed-death-receptor-1 treatment with pembrolizumab in ipilimumab-refractory advanced melanoma: a randomised dose-comparison cohort of a phase 1 trial. Lancet 2014;384:1109-17.
381. Topalian SL, Sznol M, McDermott DF, et al. Survival, durable tumor remission, and long-term safety in patients with advanced melanoma receiving nivolumab. J Clin Oncol 2014;32:1020-30.

382. Algazi AP, Tsai KK, Shoushtari AN, et al. Clinical outcomes in metastatic uveal melanoma treated with PD-1 and PD-L1 antibodies. Cancer 2016;122:3344-53.
383. Weber J, Gibney G, Kudchadkar R, et al. Phase I/II Study of Metastatic Melanoma Patients Treated with Nivolumab Who Had Progressed after Ipilimumab. Cancer Immunol Res 2016;4:345-53.
384. Kaufman HL, Russell J, Hamid O, et al. Avelumab in patients with chemotherapy-refractory metastatic Merkel cell carcinoma: a multicentre, single-group, open-label, phase 2 trial. Lancet Oncol 2016;17:1374-85.
385. Fehrenbacher L, Spira A, Ballinger M, et al. Atezolizumab versus docetaxel for patients with previously treated non-small-cell lung cancer (POPLAR): a multicentre, open-label, phase 2 randomised controlled trial. Lancet 2016;387:1837-46.
386. Borghaei H, Paz-Ares L, Horn L, et al. Nivolumab versus Docetaxel in Advanced Nonsquamous Non-Small-Cell Lung Cancer. N Engl J Med 2015;373:1627-39.
387. Brahmer J, Reckamp KL, Baas P, et al. Nivolumab versus Docetaxel in Advanced Squamous-Cell Non-Small-Cell Lung Cancer. N Engl J Med 2015;373:123-35.
388. Garon EB, Rizvi NA, Hui R, et al. Pembrolizumab for the treatment of non-small-cell lung cancer. N Engl J Med 2015;372:2018-28.
389. Gettinger SN, Horn L, Gandhi L, et al. Overall Survival and Long-Term Safety of Nivolumab (Anti-Programmed Death 1 Antibody, BMS-936558, ONO-4538) in Patients With Previously Treated Advanced Non-Small-Cell Lung Cancer. J Clin Oncol 2015;33:2004-12.
390. Gettinger S, Rizvi NA, Chow LQ, et al. Nivolumab Monotherapy for First-Line Treatment of Advanced Non-Small-Cell Lung Cancer. J Clin Oncol 2016;34:2980-7.
391. Gulley JL, Rajan A, Spigel DR, et al. Avelumab for patients with previously treated metastatic or recurrent non-small-cell lung cancer (JAVELIN Solid Tumor): dose-expansion cohort of a multicentre, open-label, phase 1b trial. Lancet Oncol 2017;18:599-610.
392. Herbst RS, Baas P, Kim DW, et al. Pembrolizumab versus docetaxel for previously treated, PD-L1-positive, advanced non-small-cell lung cancer (KEYNOTE-010): a randomised controlled trial. Lancet 2016;387:1540-50.
393. Peters S, Gettinger S, Johnson ML, et al. Phase II Trial of Atezolizumab As First-Line or Subsequent Therapy for Patients With Programmed Death-Ligand 1-Selected Advanced Non-Small-Cell Lung Cancer (BIRCH). J Clin Oncol 2017;35:2781-9.
394. Rizvi NA, Mazieres J, Planchard D, et al. Activity and safety of nivolumab, an anti-PD-1 immune checkpoint inhibitor, for patients with advanced, refractory squamous non-small-cell lung cancer (CheckMate 063): a phase 2, single-arm trial. Lancet Oncol 2015;16:257-65.
395. Motzer RJ, Rini BI, McDermott DF, et al. Nivolumab for Metastatic Renal Cell Carcinoma: Results of a Randomized Phase II Trial. J Clin Oncol 2015;33:1430-7.
396. Antonia SJ, Lopez-Martin JA, Bendell J, et al. Nivolumab alone and nivolumab plus ipilimumab in recurrent small-cell lung cancer (CheckMate 032): a multicentre, open-label, phase 1/2 trial. Lancet Oncol 2016;17:883-95.
397. Le DT, Durham JN, Smith KN, et al. Mismatch repair deficiency predicts response of solid tumors to PD-1 blockade. Science 2017;357:409-13.
398. Powles T, O'Donnell PH, Massard C, et al. Efficacy and Safety of Durvalumab in Locally Advanced or Metastatic Urothelial Carcinoma: Updated Results From a Phase 1/2 Open-label Study. JAMA Oncol 2017;3:e172411.
399. Sharma P, Callahan MK, Bono P, et al. Nivolumab monotherapy in recurrent metastatic urothelial carcinoma (CheckMate 032): a multicentre, open-label, two-stage, multi-arm, phase 1/2 trial. Lancet Oncol 2016;17:1590-8.
400. Ranson M, Davidson N, Nicolson M, et al. Randomized trial of paclitaxel plus supportive care versus supportive care for patients with advanced non-small-cell lung cancer. J Natl Cancer Inst 2000;92:1074-80.
401. Grothey A, Van Cutsem E, Sobrero A, et al. Regorafenib monotherapy for previously treated metastatic colorectal cancer (CORRECT): an international, multicentre, randomised, placebo-controlled, phase 3 trial. Lancet 2013;381:303-12.
402. Rao S, Cunningham D, de Gramont A, et al. Phase III double-blind placebo-controlled study of farnesyl transferase inhibitor R115777 in patients with refractory advanced colorectal cancer. J Clin Oncol 2004;22:3950-7.
403. Pavlakis N, Sjoquist KM, Martin AJ, et al. Regorafenib for the Treatment of Advanced Gastric Cancer (INTEGRATE): A Multinational Placebo-Controlled Phase II Trial. J Clin Oncol 2016;34:2728-35.
404. Caplin ME, Pavel M, Cwikla JB, et al. Lanreotide in metastatic enteropancreatic neuroendocrine tumors. N Engl J Med 2014;371:224-33.
405. Yao JC, Shah MH, Ito T, et al. Everolimus for advanced pancreatic neuroendocrine tumors. N Engl J Med 2011;364:514-23.
406. Abou-Alfa GK, Qin S, Ryoo BY, et al. Phase III randomized study of second line ADI-PEG 20 plus best supportive care versus placebo plus best supportive care in patients with advanced hepatocellular carcinoma. Ann Oncol 2018;29:1402-8.
407. Llovet JM, Ricci S, Mazzaferro V, et al. Sorafenib in advanced hepatocellular carcinoma. N Engl J Med 2008;359:378-90.
408. Zhu AX, Park JO, Ryoo BY, et al. Ramucirumab versus placebo as second-line treatment in patients with advanced hepatocellular carcinoma following first-line therapy with sorafenib (REACH): a randomised, double-blind, multicentre, phase 3 trial. Lancet Oncol 2015;16:859-70.
409. Eisen T, Trefzer U, Hamilton A, et al. Results of a multicenter, randomized, double-blind phase 2/3 study of lenalidomide in the treatment of pretreated relapsed or refractory metastatic malignant melanoma. Cancer 2010;116:146-54.
410. Fidias PM, Dakhil SR, Lyss AP, et al. Phase III study of immediate compared with delayed docetaxel after front-line therapy with gemcitabine plus carboplatin in advanced non-small-cell lung cancer. J Clin Oncol 2009;27:591-8.
411. Johnson EA, Marks RS, Mandrekar SJ, et al. Phase III randomized, double-blind study of maintenance CAI or placebo in patients with advanced non-small cell lung cancer (NSCLC) after completion of initial therapy (NCCTG 97-24-51). Lung Cancer 2008;60:200-7.
412. Miller VA, Hirsh V, Cadranel J, et al. Afatinib versus placebo for patients with advanced, metastatic non-small-cell lung cancer after failure of erlotinib, gefitinib, or both, and one or two lines of chemotherapy (LUX-Lung 1): a phase 2b/3 randomised trial. Lancet Oncol 2012;13:528-38.
413. O'Brien ME, Gaafar R, Hasan B, et al. Maintenance pazopanib versus placebo in Non-Small Cell Lung Cancer patients non-progressive after first line chemotherapy: A double blind randomised phase III study of the lung cancer group, EORTC 08092 (EudraCT: 2010-018566-23, NCT01208064). Eur J Cancer 2015;51:1511-28.
414. Parikh PM, Vaid A, Advani SH, et al. Randomized, double-blind, placebo-controlled phase II study of single-agent oral talactoferrin in patients with locally advanced or metastatic non-small-cell lung cancer that progressed after chemotherapy. J Clin Oncol 2011;29:4129-36.
415. Paz-Ares L, Hirsh V, Zhang L, et al. Monotherapy Administration of Sorafenib in Patients With Non-Small Cell Lung Cancer (MISSION) Trial: A Phase III, Multicenter, Placebo-Controlled Trial of Sorafenib in Patients with Relapsed or Refractory Predominantly Nonsquamous Non-Small-Cell Lung Cancer after 2 or 3 Previous Treatment Regimens. J Thorac Oncol 2015;10:1745-53.
416. Ramalingam S, Crawford J, Chang A, et al. Talactoferrin alfa versus placebo in patients with refractory advanced non-small-cell lung cancer (FORTIS-M trial). Ann Oncol 2013;24:2875-80.
417. Ledermann J, Harter P, Gourley C, et al. Olaparib maintenance therapy in platinum-sensitive relapsed ovarian cancer. N Engl J Med 2012;366:1382-92.
418. Raymond E, Dahan L, Raoul JL, et al. Sunitinib malate for the treatment of pancreatic neuroendocrine tumors. N Engl J Med 2011;364:501-13.
419. Sternberg C, Armstrong A, Pili R, et al. Randomized, Double-Blind, Placebo-Controlled Phase III Study of Tasquinimod in Men With Metastatic Castration-Resistant Prostate Cancer. J Clin Oncol 2016;34:2636-43.
420. Motzer RJ, Escudier B, Oudard S, et al. Phase 3 trial of everolimus for metastatic renal cell carcinoma : final results and analysis of prognostic factors. Cancer 2010;116:4256-65.
421. Escudier B, Eisen T, Stadler WM, et al. Sorafenib in advanced clear-cell renal-cell carcinoma. N Engl J Med 2007;356:125-34.
422. Escudier B, Eisen T, Stadler WM, et al. Sorafenib for treatment of renal cell carcinoma: Final efficacy and safety results of the phase III treatment approaches in renal cancer global evaluation trial. J Clin Oncol 2009;27:3312-8.
423. Banerjee S, Rustin G, Paul J, et al. A multicenter, randomized trial of flat dosing versus intrapatient dose escalation of single-agent carboplatin as first-line chemotherapy for advanced ovarian cancer: an SGCTG (SCOTROC 4) and ANZGOG study on behalf of GCIG. Ann Oncol 2013;24:679-87.
424. Gore M, Mainwaring P, A'Hern R, et al. Randomized trial of dose-intensity with single-agent carboplatin in patients with epithelial ovarian cancer. London Gynaecological Oncology Group. J Clin Oncol 1998;16:2426-34.
425. International Collaborative Ovarian Neoplasm G. Paclitaxel plus carboplatin versus standard chemotherapy with either single-agent carboplatin or cyclophosphamide, doxorubicin, and cisplatin in women with ovarian cancer: the ICON3 randomised trial. Lancet 2002;360:505-15.
426. Lambert HE, Rustin GJ, Gregory WM, Nelstrop AE. A randomized trial of five versus eight courses of cisplatin or carboplatin in advanced epithelial ovarian carcinoma. A North Thames Ovary Group Study. Ann Oncol 1997;8:327-33.
427. Muggia FM, Braly PS, Brady MF, et al. Phase III randomized study of cisplatin versus paclitaxel versus cisplatin and paclitaxel in patients with suboptimal stage III or IV ovarian cancer: a gynecologic oncology group study. J Clin Oncol 2000;18:106-15.
428. Rankin EM, Mill L, Kaye SB, et al. A randomised study comparing standard dose carboplatin with chlorambucil and carboplatin in advanced ovarian cancer. Br J Cancer 1992;65:275-81.
429. Skarlos DV, Aravantinos G, Kosmidis P, et al. Carboplatin alone compared with its combination with epirubicin and cyclophosphamide in untreated advanced epithelial ovarian cancer: a Hellenic co-operative oncology group study. Eur J Cancer 1996;32A:421-8.
430. Horwich A, Oliver RT, Wilkinson PM, et al. A medical research council randomized trial of single agent carboplatin versus etoposide and cisplatin for advanced metastatic seminoma. MRC Testicular Tumour Working Party. Br J Cancer 2000;83:1623-9.
431. Baselga J, Gomez P, Greil R, et al. Randomized phase II study of the anti-epidermal growth factor receptor monoclonal antibody cetuximab with cisplatin versus cisplatin alone in patients with metastatic triple-negative breast cancer. J Clin Oncol 2013;31:2586-92.
432. Long HJ, 3rd, Bundy BN, Grendys EC, Jr., et al. Randomized phase III trial of cisplatin with or without topotecan in carcinoma of the uterine cervix: a Gynecologic Oncology Group Study. J Clin Oncol 2005;23:4626-33.
433. Moore DH, Blessing JA, McQuellon RP, et al. Phase III study of cisplatin with or without paclitaxel in stage IVB, recurrent, or persistent squamous cell carcinoma of the cervix: a gynecologic oncology group study. J Clin Oncol 2004;22:3113-9.

434. Omura GA, Blessing JA, Vaccarello L, et al. Randomized trial of cisplatin versus cisplatin plus mitolactol versus cisplatin plus ifosfamide in advanced squamous carcinoma of the cervix: a Gynecologic Oncology Group study. J Clin Oncol 1997;15:165-71.
435. Burtness B, Goldwasser MA, Flood W, Mattar B, Forastiere AA, Eastern Cooperative Oncology G. Phase III randomized trial of cisplatin plus placebo compared with cisplatin plus cetuximab in metastatic/recurrent head and neck cancer: an Eastern Cooperative Oncology Group study. J Clin Oncol 2005;23:8646-54.
436. Patil VM, Noronha V, Joshi A, et al. A prospective randomized phase II study comparing metronomic chemotherapy with chemotherapy (single agent cisplatin), in patients with metastatic, relapsed or inoperable squamous cell carcinoma of head and neck. Oral Oncol 2015;51:279-86.
437. Urba S, van Herpen CM, Sahoo TP, et al. Pemetrexed in combination with cisplatin versus cisplatin monotherapy in patients with recurrent or metastatic head and neck cancer: final results of a randomized, double-blind, placebo-controlled, phase 3 study. Cancer 2012;118:4694-705.
438. Urba S, Hong RL, Hossain AM, Cheng R, Orlando M. Pemetrexed in combination with cisplatin versus cisplatin monotherapy in East Asian patients with recurrent or metastatic head and neck cancer: Results of an exploratory subgroup analysis of a phase III trial. Asia Pac J Clin Oncol 2013;9:331-41.
439. Gatzemeier U, von Pawel J, Gottfried M, et al. Phase III comparative study of high-dose cisplatin versus a combination of paclitaxel and cisplatin in patients with advanced non-small-cell lung cancer. J Clin Oncol 2000;18:3390-9.
440. Sandler AB, Nemunaitis J, Denham C, et al. Phase III trial of gemcitabine plus cisplatin versus cisplatin alone in patients with locally advanced or metastatic non-small-cell lung cancer. J Clin Oncol 2000;18:122-30.
441. von Pawel J, von Roemeling R, Gatzemeier U, et al. Tirapazamine plus cisplatin versus cisplatin in advanced non-small-cell lung cancer: A report of the international CATAPULT I study group. Cisplatin and Tirapazamine in Subjects with Advanced Previously Untreated Non-Small-Cell Lung Tumors. J Clin Oncol 2000;18:1351-9.
442. Wozniak AJ, Crowley JJ, Balcerzak SP, et al. Randomized trial comparing cisplatin with cisplatin plus vinorelbine in the treatment of advanced non-small-cell lung cancer: a Southwest Oncology Group study. J Clin Oncol 1998;16:2459-65.
443. Bolis G, Scarfone G, Giardina G, et al. Carboplatin alone vs carboplatin plus epidoxorubicin as second-line therapy for cisplatin- or carboplatin-sensitive ovarian cancer. Gynecol Oncol 2001;81:3-9.
444. Pfisterer J, Plante M, Vergote I, et al. Gemcitabine plus carboplatin compared with carboplatin in patients with platinum-sensitive recurrent ovarian cancer: an intergroup trial of the AGO-OVAR, the NCIC CTG, and the EORTC GCG. J Clin Oncol 2006;24:4699-707.
445. Loehrer PJ, Sr., Einhorn LH, Elson PJ, et al. A randomized comparison of cisplatin alone or in combination with methotrexate, vinblastine, and doxorubicin in patients with metastatic urothelial carcinoma: a cooperative group study. J Clin Oncol 1992;10:1066-73.
446. Abou-Alfa GK, Schwartz L, Ricci S, et al. Phase II study of sorafenib in patients with advanced hepatocellular carcinoma. J Clin Oncol 2006;24:4293-300.
447. Cheng AL, Kang YK, Lin DY, et al. Sunitinib versus sorafenib in advanced hepatocellular cancer: results of a randomized phase III trial. J Clin Oncol 2013;31:4067-75.
448. Dingemans AM, Mellema WW, Groen HJ, et al. A phase II study of sorafenib in patients with platinum-pretreated, advanced (Stage IIIb or IV) non-small cell lung cancer with a KRAS mutation. Clin Cancer Res 2013;19:743-51.
449. Zhou Q, Zhou CC, Chen GY, et al. A multicenter phase II study of sorafenib monotherapy in clinically selected patients with advanced lung adenocarcinoma after failure of EGFR-TKI therapy (Chinese Thoracic Oncology Group, CTONG 0805). Lung Cancer 2014;83:369-73.
450. Procopio G, Verzoni E, Bracarda S, et al. Sorafenib with interleukin-2 vs sorafenib alone in metastatic renal cell carcinoma: the ROSORC trial. Br J Cancer 2011;104:1256-61.
451. Maki RG, D'Adamo DR, Keohan ML, et al. Phase II study of sorafenib in patients with metastatic or recurrent sarcomas. J Clin Oncol 2009;27:3133-40.
452. Smith M, De Bono J, Sternberg C, et al. Phase III Study of Cabozantinib in Previously Treated Metastatic Castration-Resistant Prostate Cancer: COMET-1. J Clin Oncol 2016;34:3005-13.
453. Blackwell KL, Pegram MD, Tan-Chiu E, et al. Single-agent lapatinib for HER2-overexpressing advanced or metastatic breast cancer that progressed on first- or second-line trastuzumab-containing regimens. Ann Oncol 2009;20:1026-31.
454. Kaufman B, Trudeau M, Awada A, et al. Lapatinib monotherapy in patients with HER2-overexpressing relapsed or refractory inflammatory breast cancer: final results and survival of the expanded HER2+ cohort in EGF103009, a phase II study. Lancet Oncol 2009;10:581-8.
455. Fassnacht M, Berruti A, Baudin E, et al. Linsitinib (OSI-906) versus placebo for patients with locally advanced or metastatic adrenocortical carcinoma: a double-blind, randomised, phase 3 study. Lancet Oncol 2015;16:426-35.
456. Michaelson MD, Oudard S, Ou YC, et al. Randomized, placebo-controlled, phase III trial of sunitinib plus prednisone versus prednisone alone in progressive, metastatic, castration-resistant prostate cancer. J Clin Oncol 2014;32:76-82.
457. Burstein HJ, Elias AD, Rugo HS, et al. Phase II study of sunitinib malate, an oral multitargeted tyrosine kinase inhibitor, in patients with metastatic breast cancer previously treated with an anthracycline and a taxane. J Clin Oncol 2008;26:1810-6.
458. Ansari J, Fatima A, Fernando K, Collins S, James ND, Porfiri E. Sunitinib in patients with metastatic renal cell carcinoma: Birmingham experience. Oncol Rep 2010;24:507-10.
459. Buzdar A, Hayes D, El-Khoudary A, et al. Phase III randomized trial of droloxifene and tamoxifen as first-line endocrine treatment of ER/PgR-positive advanced breast cancer. Breast Cancer Res Treat 2002;73:161-75.
460. Gill PG, Gebski V, Snyder R, et al. Randomized comparison of the effects of tamoxifen, megestrol acetate, or tamoxifen plus megestrol acetate on treatment response and survival in patients with metastatic breast cancer. Ann Oncol 1993;4:741-4.
461. Ingle JN, Suman VJ, Kardinal CG, et al. A randomized trial of tamoxifen alone or combined with octreotide in the treatment of women with metastatic breast carcinoma. Cancer 1999;85:1284-92.
462. Klijn JG, Beex LV, Mauriac L, et al. Combined treatment with buserelin and tamoxifen in premenopausal metastatic breast cancer: a randomized study. J Natl Cancer Inst 2000;92:903-11.
463. Lindemann K, Gibbs E, Avall-Lundqvist E, et al. Chemotherapy vs tamoxifen in platinum-resistant ovarian cancer: a phase III, randomised, multicentre trial (Ovaresist). Br J Cancer 2017;116:455-63.
464. Rose C, Kamby C, Mouridsen HT, et al. Combined endocrine treatment of elderly postmenopausal patients with metastatic breast cancer. A randomized trial of tamoxifen vs. tamoxifen + aminoglutethimide and hydrocortisone and tamoxifen + fluoxymesterone in women above 65 years of age. Breast Cancer Res Treat 2000;61:103-10.
465. Thigpen T, Brady MF, Homesley HD, Soper JT, Bell J. Tamoxifen in the treatment of advanced or recurrent endometrial carcinoma: a Gynecologic Oncology Group study. J Clin Oncol 2001;19:364-7.
466. Adachi I, Watanabe T, Takashima S, et al. A late phase II study of RP56976 (docetaxel) in patients with advanced or recurrent breast cancer. Br J Cancer 1996;73:210-6.
467. Kruijtzer CMF, Verweij J, Schellens JH, et al. Docetaxel in 253 previously treated patients with progressive locally advanced or metastatic breast cancer: results of a compassionate use program in The Netherlands. Anticancer Drugs 2000;11:249-55.
468. Nabholtz JM, Gelmon K, Bontenbal M, et al. Multicenter, randomized comparative study of two doses of paclitaxel in patients with metastatic breast cancer. J Clin Oncol 1996;14:1858-67.
469. Nabholtz JM, Senn HJ, Bezwoda WR, et al. Prospective randomized trial of docetaxel versus mitomycin plus vinblastine in patients with metastatic breast cancer progressing despite previous anthracycline-containing chemotherapy. 304 Study Group. J Clin Oncol 1999;17:1413-24.
470. Seidman AD, Berry D, Cirrincione C, et al. Randomized phase III trial of weekly compared with every-3-weeks paclitaxel for metastatic breast cancer, with trastuzumab for all HER-2 overexpressors and random assignment to trastuzumab or not in HER-2 nonoverexpressors: final results of Cancer and Leukemia Group B protocol 9840. J Clin Oncol 2008;26:1642-9.
471. Sjostrom J, Blomqvist C, Mouridsen H, et al. Docetaxel compared with sequential methotrexate and 5-fluorouracil in patients with advanced breast cancer after anthracycline failure: a randomised phase III study with crossover on progression by the Scandinavian Breast Group. Eur J Cancer 1999;35:1194-201.
472. Camps C, Massuti B, Jimenez A, et al. Randomized phase III study of 3-weekly versus weekly docetaxel in pretreated advanced non-small-cell lung cancer: a Spanish Lung Cancer Group trial. Ann Oncol 2006;17:467-72.
473. Fossella FV, DeVore R, Kerr RN, et al. Randomized phase III trial of docetaxel versus vinorelbine or ifosfamide in patients with advanced non-small-cell lung cancer previously treated with platinum-containing chemotherapy regimens. The TAX 320 Non-Small Cell Lung Cancer Study Group. J Clin Oncol 2000;18:2354-62.
474. Herbst RS, Sun Y, Eberhardt WE, et al. Vandetanib plus docetaxel versus docetaxel as second-line treatment for patients with advanced non-small-cell lung cancer (ZODIAC): a double-blind, randomised, phase 3 trial. Lancet Oncol 2010;11:619-26.
475. Lee DH, Park K, Kim JH, et al. Randomized Phase III trial of gefitinib versus docetaxel in non-small cell lung cancer patients who have previously received platinum-based chemotherapy. Clin Cancer Res 2010;16:1307-14.
476. Lilenbaum R, Rubin M, Samuel J, et al. A randomized phase II trial of two schedules of docetaxel in elderly or poor performance status patients with advanced non-small cell lung cancer. J Thorac Oncol 2007;2:306-11.
477. Pectasides D, Pectasides M, Farmakis D, et al. Comparison of docetaxel and docetaxel-irinotecan combination as second-line chemotherapy in advanced non-small-cell lung cancer: a randomized phase II trial. Ann Oncol 2005;16:294-9.
478. Schuette W, Nagel S, Blankenburg T, et al. Phase III study of second-line chemotherapy for advanced non-small-cell lung cancer with weekly compared with 3-weekly docetaxel. J Clin Oncol 2005;23:8389-95.
479. Buda A, Floriani I, Rossi R, et al. Randomised controlled trial comparing single agent paclitaxel vs epidoxorubicin plus paclitaxel in patients with advanced ovarian cancer in early progression after platinum-based chemotherapy: an Italian Collaborative Study from the Mario Negri Institute, Milan, G.O.N.O. (Gruppo Oncologico Nord Ovest) group and I.O.R. (Istituto Oncologico Romagnolo) group. Br J Cancer 2004;90:2112-7.
480. Eisenhauer EA, ten Bokkel Huinink WW, Swenerton KD, et al. European-Canadian randomized trial of paclitaxel in relapsed ovarian cancer: high-dose versus low-dose and long versus short infusion. J Clin Oncol 1994;12:2654-66.

481. Rosenberg P, Andersson H, Boman K, et al. Randomized trial of single agent paclitaxel given weekly versus every three weeks and with peroral versus intravenous steroid premedication to patients with ovarian cancer previously treated with platinum. Acta Oncol 2002;41:418-24.
482. ten Bokkel Huinink W, Gore M, Carmichael J, et al. Topotecan versus paclitaxel for the treatment of recurrent epithelial ovarian cancer. J Clin Oncol 1997;15:2183-93.
483. Trope C, Hogberg T, Kaern J, et al. Long-term results from a phase II study of single agent paclitaxel (Taxol) in previously platinum treated patients with advanced ovarian cancer: the Nordic experience. Ann Oncol 1998;9:1301-7.
484. Mendez M, Salut A, Garcia-Giron C, et al. A multicenter phase II study of irinotecan in patients with advanced colorectal cancer previously treated with 5-fluorouracil. Clin Colorectal Cancer 2003;3:174-9.
485. Seymour MT, Brown SR, Middleton G, et al. Panitumumab and irinotecan versus irinotecan alone for patients with KRAS wild-type, fluorouracil-resistant advanced colorectal cancer (PICCOLO): a prospectively stratified randomised trial. Lancet Oncol 2013;14:749-59.
486. Higuchi K, Tanabe S, Shimada K, et al. Biweekly irinotecan plus cisplatin versus irinotecan alone as second-line treatment for advanced gastric cancer: a randomised phase III trial (TCOG GI-0801/BIRIP trial). Eur J Cancer 2014;50:1437-45.
487. Nishikawa K, Fujitani K, Inagaki H, et al. Randomised phase III trial of second-line irinotecan plus cisplatin versus irinotecan alone in patients with advanced gastric cancer refractory to S-1 monotherapy: TRICS trial. Eur J Cancer 2015;51:808-16.
488. Tanabe K, Fujii M, Nishikawa K, et al. Phase II/III study of second-line chemotherapy comparing irinotecan-alone with S-1 plus irinotecan in advanced gastric cancer refractory to first-line treatment with S-1 (JACCRO GC-05). Ann Oncol 2015;26:1916-22.
489. Gligorov J, Doval D, Bines J, et al. Maintenance capecitabine and bevacizumab versus bevacizumab alone after initial first-line bevacizumab and docetaxel for patients with HER2-negative metastatic breast cancer (IMELDA): a randomised, open-label, phase 3 trial. Lancet Oncol 2014;15:1351-60.
490. Johnsson A, Hagman H, Frodin JE, et al. A randomized phase III trial on maintenance treatment with bevacizumab alone or in combination with erlotinib after chemotherapy and bevacizumab in metastatic colorectal cancer: the Nordic ACT Trial. Ann Oncol 2013;24:2335-41.
491. Tournigand C, Chibaudel B, Samson B, et al. Bevacizumab with or without erlotinib as maintenance therapy in patients with metastatic colorectal cancer (GERCOR DREAM; OPTIMOX3): a randomised, open-label, phase 3 trial. Lancet Oncol 2015;16:1493-505.
492. Cloughesy T, Finocchiaro G, Belda-Iniesta C, et al. Randomized, Double-Blind, Placebo-Controlled, Multicenter Phase II Study of Onartuzumab Plus Bevacizumab Versus Placebo Plus Bevacizumab in Patients With Recurrent Glioblastoma: Efficacy, Safety, and Hepatocyte Growth Factor and O(6)-Methylguanine-DNA Methyltransferase Biomarker Analyses. J Clin Oncol 2017;35:343-51.
493. Friedman HS, Prados MD, Wen PY, et al. Bevacizumab alone and in combination with irinotecan in recurrent glioblastoma. J Clin Oncol 2009;27:4733-40.
494. Carvajal RD, Wong MK, Thompson JA, et al. A phase 2 randomised study of ramucirumab (IMC-1121B) with or without dacarbazine in patients with metastatic melanoma. Eur J Cancer 2014;50:2099-107.
495. Barlesi F, Scherpereel A, Rittmeyer A, et al. Randomized phase III trial of maintenance bevacizumab with or without pemetrexed after first-line induction with bevacizumab, cisplatin, and pemetrexed in advanced nonsquamous non-small-cell lung cancer: AVAPERL (MO22089). J Clin Oncol 2013;31:3004-11.
496. Tew WP, Sill MW, Walker JL, et al. Randomized phase II trial of bevacizumab plus everolimus versus bevacizumab alone for recurrent or persistent ovarian, fallopian tube or peritoneal carcinoma: An NRG oncology/gynecologic oncology group study. Gynecol Oncol 2018;151:257-63.
497. Bukowski RM, Kabbinavar FF, Figlin RA, et al. Randomized phase II study of erlotinib combined with bevacizumab compared with bevacizumab alone in metastatic renal cell cancer. J Clin Oncol 2007;25:4536-41.
498. Flaherty KT, Manola JB, Pins M, et al. BEST: A Randomized Phase II Study of Vascular Endothelial Growth Factor, RAF Kinase, and Mammalian Target of Rapamycin Combination Targeted Therapy With Bevacizumab, Sorafenib, and Temsirolimus in Advanced Renal Cell Carcinoma--A Trial of the ECOG-ACRIN Cancer Research Group (E2804). J Clin Oncol 2015;33:2384-91.
499. Hainsworth JD, Shipley DL, Reeves J, Jr., Arrowsmith ER, Barnes EK, Waterhouse DM. High-dose bevacizumab in the treatment of patients with advanced clear cell renal carcinoma: a phase II trial of the Sarah Cannon Oncology Research Consortium. Clin Genitourin Cancer 2013;11:283-9 e1.
500. Yang JC, Haworth L, Sherry RM, et al. A randomized trial of bevacizumab, an anti-vascular endothelial growth factor antibody, for metastatic renal cancer. N Engl J Med 2003;349:427-34.
501. Fumoleau P, Delgado FM, Delozier T, et al. Phase II trial of weekly intravenous vinorelbine in first-line advanced breast cancer chemotherapy. J Clin Oncol 1993;11:1245-52.
502. Garcia-Conde J, Lluch A, Martin M, et al. Phase II trial of weekly IV vinorelbine in first-line advanced breast cancer chemotherapy. Ann Oncol 1994;5:854-7.
503. Gasparini G, Caffo O, Barni S, et al. Vinorelbine is an active antiproliferative agent in pretreated advanced breast cancer patients: a phase II study. J Clin Oncol 1994;12:2094-101.
504. Jones S, Winer E, Vogel C, et al. Randomized comparison of vinorelbine and melphalan in anthracycline-refractory advanced breast cancer. J Clin Oncol 1995;13:2567-74.
505. Martin M, Ruiz A, Munoz M, et al. Gemcitabine plus vinorelbine versus vinorelbine monotherapy in patients with metastatic breast cancer previously treated with anthracyclines and taxanes: final results of the phase III Spanish Breast Cancer Research Group (GEICAM) trial. Lancet Oncol 2007;8:219-25.
506. Toi M, Saeki T, Aogi K, et al. Late phase II clinical study of vinorelbine monotherapy in advanced or recurrent breast cancer previously treated with anthracyclines and taxanes. Jpn J Clin Oncol 2005;35:310-5.
507. Chen YM, Tsai CM, Fan WC, et al. Phase II randomized trial of erlotinib or vinorelbine in chemonaive, advanced, non-small cell lung cancer patients aged 70 years or older. J Thorac Oncol 2012;7:412-8.
508. Depierre A, Chastang C, Quoix E, et al. Vinorelbine versus vinorelbine plus cisplatin in advanced non-small cell lung cancer: a randomized trial. Ann Oncol 1994;5:37-42.

Supplementary Online Table 3. Tumor type, therapy type, prior therapy for advanced disease, and the Proportion of remaining patients progressing since prior scan for different time intervals between scans

Tumor	Rx†	no. studies	median PFS $t_{1/2}$ months	lowest PFS $t_{1/2}$ months	highest PFS $t_{1/2}$ months	median PFS $t_{1/2}$ weeks	Time (weeks) from last scan					
							3 wk	6 wk	9 wk	12 wk	18 wk	24 wk
							Percent of remaining patients progressing since prior scan					
**No prior systemic Rx for advanced disease**												
adrenal	combinations	2	4.4	2.7	6.1	19.1	10	20	28	35	48	58
breast	anthracycline	4	6	5.1	9.7	26.0	8	15	21	27	38	47
breast	capecitabine	1	6.4			27.7	7	14	20	26	36	45
breast	taxanes	6	5.8	3.4	9.7	25.1	8	15	22	28	39	48
breast	vinorelbine	2	4.8	4.5	5.1	20.8	10	18	26	33	45	55
breast	combinations	35	14.1	5.9	21.8	61.1	3	7	10	13	18	24
breast ER+	tamoxifen	10	8	6.5	15.1	34.7	6	11	16	21	30	38
breast ER+	Fulvestrant	4	18.8	7.2	20.6	81.5	3	5	7	10	14	18
breast ER+	Aromatase inhibitors	18	12.9	4.9	21.5	55.9	4	7	11	14	20	26
breast HER2+	trastuzumab	3	4.2	4.1	5.5	18.2	11	20	29	37	50	60
breast HER2+	T-DM1	1	14.7			63.7	3	6	9	12	18	23
cervix	cisplatin	1	3.2			13.9	14	26	36	45	59	70
cervix	combinations	4	5.8	4.3	7.4	25.1	8	15	22	28	39	48
colon	capecitabine	1	5.2			22.5	9	17	24	31	43	52
colon	combination	23	8.3	5.4	11.6	36.0	6	11	16	21	29	37
endometrium	doxorubicin	2	3.8	3.4	4.2	16.5	12	22	32	40	53	64
endometrium	tamoxifen	1	2.5			10.8	17	32	44	54	68	78
gastric	combination	4	5.5	4.9	6.2	23.8	8	16	23	29	41	50
GI/lung neuroendocrine	everolimus	2	12.4	12.2	12.6	53.7	4	7	11	14	21	27
GI/lung neuroendocrine	lanreotide	1	34.9			151.2	1	3	4	5	8	10
GI/lung neuroendocrine	placebo	1	18.9			81.9	3	5	7	10	14	18
glioma	temozolamide	2	4.3	3.7	4.9	18.6	11	20	28	36	49	59
glioma	XRT + temoz	2	7.2	6.8	7.6	31.2	6	12	18	23	33	41
glioma	XRT+temoz+bev	2	10.2	10.2	10.2	44.2	5	9	13	17	25	31
glioma	PCV	1	3.7			16.0	12	23	32	40	54	65
head/neck	cisplatin	4	2.5	2.1	3.1	10.8	17	32	44	54	68	78
head/neck	combination	2	4	3.2	4.8	17.3	11	21	30	38	51	62
hepatocellular	sorafenib	3	4.6	4	5.6	19.9	10	19	27	34	47	57
hepatocellular	linafanib	1	4.8			20.8	10	18	26	33	45	55
hepatocellular	doxorubicin	2	3.4	2.6	4.1	14.7	13	25	35	43	57	68

Continuation of Supplementary Online Table 3. Tumor type, therapy type, prior therapy for advanced disease, and the Proportion of remaining patients progressing since prior scan for different time intervals between scans												
Tumor	Rx†	no. studies	median PFS $t_{1/2}$ months	lowest PFS $t_{1/2}$ months	highest PFS $t_{1/2}$ months	median PFS $t_{1/2}$ weeks	Time (weeks) from last scan					
							3 wk	6 wk	9 wk	12 wk	18 wk	24 wk
							Percent of remaining patients progressing since prior scan					
hepatocellular	nolatrexed	1	3.9			16.9	12	22	31	39	52	63
hepatocellular	FOLFOX	1	3.6			15.6	12	23	33	41	55	66
hepatocellular	placebo	1	3.3			14.3	14	25	35	44	58	69
melanoma	Dacarbazine	4	2.5	2	3.1	10.8	17	32	44	54	68	78
melanoma	cisp+vinbl+dacarb	1	2.8			12.1	16	29	40	50	64	75
melanoma	IL2+IFN+chemo	1	4.6			19.9	10	19	27	34	47	57
melanoma	Ipilimumab	4	4.5	3.6	4.7	19.5	10	19	27	35	47	57
melanoma	ipi+dacarb	1	4			17.3	11	21	30	38	51	62
melanoma	PD1/PDL1 mono	6	10.4	6.5	14.6	45.1	5	9	13	17	24	31
melanoma	nivol+ipi	7	14.5	11.3	21.3	62.8	3	6	9	12	18	23
melanoma	Ramucirumab	1	2.1			9.1	20	37	50	60	75	84
melanoma BRAF-m	vemur or dabra	5	6.7	3.5	8.8	29.0	7	13	19	25	35	44
melanoma BRAF-m	Trametinib	1	4.7			20.4	10	18	26	34	46	56
melanoma BRAF-m	BRAF+MEK inhib	3	11.9	10.9	12.5	51.6	4	8	11	15	21	28
NSCLC	Cisplatin	4	2.6	2.4	3.8	11.3	17	31	43	52	67	77
NSCLC	Pemetrexed	1	2.7			11.7	16	30	41	51	66	76
NSCLC	Gemcitabine	1	3.8			16.5	12	22	32	40	53	64
NSCLC	Taxanes	5	3.6	2.2	4.4	15.6	12	23	33	41	55	66
NSCLC	vinorelbine	3	3.1	3	3.1	13.4	14	27	37	46	60	71
NSCLC	combinations	53	5	3	8.9	21.7	9	17	25	32	44	54
NSCLC	PD1/PDL1 mono	4	5.2	4.7	9.4	22.5	9	17	24	31	43	52
NSCLC	PD1/PDL1 mono + chemo	3	8.6	7.7	9	37.3	5	11	15	20	28	36
NSCLC	ipi + chemo	2	6	5.4	6.6	26.0	8	15	21	27	38	47
NSCLC ALK+	crizotinib	6	10.8	10.6	24.9	46.8	4	9	12	16	23	30
NSCLC ALK+	other ALK TKI	4	21.7	16.8	28.3	94.0	2	4	6	8	12	16
NSCLC-ROS1+	crizotinib	1	20			86.7	2	5	7	9	13	17
NSCLC EGFR-m	gefit/erlot/afat	16	10.4	8.1	14.2	45.1	5	9	13	17	24	31
NSCLC EGFR-m	dacomitinib	1	14.8			64.1	3	6	9	12	18	23
NSCLC EGFR-m	osimertinib	1	21.1			91.4	2	4	7	9	13	17
NSCLC EGFR-u	gefit or erlot	15	3.1	1.6	6.5	13.4	14	27	37	46	60	71
NSCLC EGFR WT	EGFR TKI	3	1.9	0.7	2.1	8.2	22	40	53	64	78	87

Continuation of Supplementary Online Table 3. Tumor type, therapy type, prior therapy for advanced disease, and the Proportion of remaining patients progressing since prior scan for different time intervals between scans

Tumor	Rx†	no. studies	median PFS $t_{1/2}$ months	lowest PFS $t_{1/2}$ months	highest PFS $t_{1/2}$ months	median PFS $t_{1/2}$ weeks	Time (weeks) from last scan					
							3 wk	6 wk	9 wk	12 wk	18 wk	24 wk
							Percent of remaining patients progressing since prior scan					
NSCLC	placebo/BSC	3	1.4	1.3	2.5	6.1	29	50	64	75	87	94
ovary	carbo/cisplatin	13	14.9	11.1	19.3	64.6	3	6	9	12	18	23
ovary	paclitaxel	1	11.2			48.5	4	8	12	16	23	29
ovary	platinum combo	5	20.1	12	23.8	87.1	2	5	7	9	13	17
pancreas	gemcitabine	14	3.6	2.8	6.4	15.6	12	23	33	41	55	66
pancreas	S1	1	3.4			14.7	13	25	35	43	57	68
pancreas	combination	9	4.9	3.2	5.5	21.2	9	18	25	32	44	54
Prostate	antiandrogen	1	23.8			103.1	2	4	6	8	11	15
Prostate	docet+anitandro	1	37			160.3	1	3	4	5	7	10
renal	interferon	7	5.5	2.2	6.4	23.8	8	16	23	29	41	50
renal	bevacizumab	1	9.1			39.4	5	10	15	19	27	34
renal	bev+IFN	2	8.7	8.3	9.1	37.7	5	10	15	20	28	36
renal	ipi+nivol	1	12.7			55.0	4	7	11	14	20	26
renal	mTOR inh	3	4.7	4.2	7.6	20.4	10	18	26	34	46	56
renal	sorafenib	2	7.5	5.9	9.1	32.5	6	12	17	23	32	40
renal	sunitinib	3	10.5	10.3	12.5	45.5	4	9	13	17	24	31
sarcoma	doxorubicin	5	4.8	3	5.2	20.8	10	18	26	33	45	55
SCLC	platinum combo	8	5			21.7	9	17	25	32	44	54
seminoma	carboplatin	1	88.1			381.8	1	1	2	2	3	4
TMB high	ipi+nivol	1	8.8			38.1	5	10	15	20	28	35
urothelial	cisplatin	1	2.8			12.1	16	29	40	50	64	75
**Prior systemic Rx for advanced disease**												
adrenal	lisitinib	1	2.2			9.5	20	35	48	58	73	83
biliary	gemcitabine	1	4.7			20.4	10	18	26	34	46	56
biliary	cisp+gem	1	6.6			28.6	7	14	20	25	35	44
breast	anthracycline	4	3.6	2.7	5	15.6	12	23	33	41	55	66
breast	capecitabine	2	4.5	4.3	4.7	19.5	10	19	27	35	47	57
breast	taxanes	13	4.6			19.9	10	19	27	34	47	57
breast	vinorelbine	4	3.9	2.8	5.3	16.9	12	22	31	39	52	63
breast	combinations	12	6.5	2.9	11.2	28.2	7	14	20	26	36	45
breast	sunitinib	1	2.9			12.6	15	28	39	48	63	73

Continuation of Supplementary Online Table 3. Tumor type, therapy type, prior therapy for advanced disease, and the Proportion of remaining patients progressing since prior scan for different time intervals between scans

Tumor	Rx†	no. studies	median PFS $t_{1/2}$ months	lowest PFS $t_{1/2}$ months	highest PFS $t_{1/2}$ months	median PFS $t_{1/2}$ weeks	Time (weeks) from last scan					
							3 wk	6 wk	9 wk	12 wk	18 wk	24 wk
							Percent of remaining patients progressing since prior scan					
breast ER+	Fulvestrant	22	5.4	2.4	11.2	23.4	9	16	23	30	41	51
breast ER+	Exemestane	2	4.2	3.9	4.4	18.2	11	20	29	37	50	60
breast ER+	Aromatase inhibitor	11	5.8	3.7	9.2	25.1	8	15	22	28	39	48
breast HER2+	lapatinib	3	3.3	1.9	3.3	14.3	14	25	35	44	58	69
breast HER2+	T-DM1	2	7.8	5.9	9.6	33.8	6	12	17	22	31	39
colon	irinotecan	5	4.1	2.6	5.7	17.8	11	21	30	37	50	61
colon	TAS-102	1	2.5			10.8	17	32	44	54	68	78
colon	combination	12	4.7	3.5	6.1	20.4	10	18	26	34	46	56
colon	EGFR mono	12	2.5	1.6	3.6	10.8	17	32	44	54	68	78
colon	regorafenib	1	2.4			10.4	18	33	45	55	70	80
colon	R115777	1	2.5			10.8	17	32	44	54	68	78
colon MSI-high	nivolumab	1	12.5			54.2	4	7	11	14	21	26
colon	placebo/BSC	9	1.5	1.3	2.4	6.5	27	47	62	72	85	92
gastric	irinotecan	3	4	3.5	10	17.3	11	21	30	38	51	62
gastric	regorafenib	1	2.4			10.4	18	33	45	55	70	80
gastric	apatinib	1	3			13.0	15	27	38	47	62	72
gastric	everolimus	2	2.3	2.1	2.5	10.0	19	34	47	57	71	81
gastric	Ipilimumab	1	3.2			13.9	14	26	36	45	59	70
gastric	pembrolizumab	1	2.6			11.3	17	31	43	52	67	77
gastric	placebo	3	1.5	1	1.6	6.5	27	47	62	72	85	92
GI/lung neuroendocrine	everolimus	3	11.9	9	12.1	51.6	4	8	11	15	21	28
GI/pancreas/lung neuroendocrine	placebo	2	5.2	4.9	5.4	22.5	9	17	24	31	43	52
GIST	sunitinib	3	6.4	5.3	9.1	27.7	7	14	20	26	36	45
GIST	regorafenib	1	4.5			19.5	10	19	27	35	47	57
GIST	placebo	2	1.5	1.2	1.8	6.5	27	47	62	72	85	92
glioma	bevacizumab	1	4.3			18.6	11	20	28	36	49	59
head/neck	chemo or cetux	1	2.5			10.8	17	32	44	54	68	78
head/neck	EGFR mono	2	2.6	2.4	2.8	11.3	17	31	43	52	67	77
head/neck	ramucirumab	1	3.9			16.9	12	22	31	39	52	63

Continuation of Supplementary Online Table 3. Tumor type, therapy type, prior therapy for advanced disease, and the Proportion of remaining patients progressing since prior scan for different time intervals between scans												
Tumor	Rx†	no. studies	median PFS $t_{1/2}$ months	lowest PFS $t_{1/2}$ months	highest PFS $t_{1/2}$ months	median PFS $t_{1/2}$ weeks	Time (weeks) from last scan					
							3 wk	6 wk	9 wk	12 wk	18 wk	24 wk
							Percent of remaining patients progressing since prior scan					
head/neck	PD1/PDL1 mono	4	2.8	2.5	2.9	12.1	16	29	40	50	64	75
head/neck	BSC	1	2.2			9.5	20	35	48	58	73	83
Hepatocellular	sorafenib	1	3.8			16.5	12	22	32	40	53	64
Hepatocellular	sunitinib	1	3.4			14.7	13	25	35	43	57	68
Hepatocellular	everolimus	1	3.3			14.3	14	25	35	44	58	69
Hepatocellular	brivanib	1	4			17.3	11	21	30	38	51	62
hepatocellular	placebo	3	2.6	2.4	3.2	11.3	17	31	43	52	67	77
melanoma	PD1/PDL1 mono	8	5.4	4.2	8.2	23.4	9	16	23	30	41	51
melanoma	ipilimumab	3	4.7	3.2	5.1	20.4	10	18	26	34	46	56
melanoma	ipi+pembro	1	21.5			93.2	2	4	6	9	13	16
melanoma	gp100	1	2.8			12.1	16	29	40	50	64	75
melanoma	ipi+gp100	1	2.6			11.3	17	31	43	52	67	77
melanoma BRAF-m	vemurafenib	2	5.6	4.5	6.6	24.3	8	16	23	29	40	50
melanoma	placebo	1	2.6			11.3	17	31	43	52	67	77
Merkel	avelumab	1	3.8			16.5	12	22	32	40	53	64
mesothelioma	pemetrexed	1	3.4			14.7	13	25	35	43	57	68
mesothelioma	everolimus	1	3.1			13.4	14	27	37	46	60	71
mesothelioma	BSC	1	2.1			9.1	20	37	50	60	75	84
NSCLC	sorafenib	3	3	2.5	3.2	13.0	15	27	38	47	62	72
NSCLC	taxanes	36	3.6	2.3	6.5	15.6	12	23	33	41	55	66
NSCLC	pemetrexed	16	3.4	2.6	5.8	14.7	13	25	35	43	57	68
NSCLC PDL1 unspecified	PD1/PDL1 mono	7	4.1	3.3	4.7	17.8	11	21	30	37	50	61
NSCLC PDL1 below an upper limit	PD1/PDL1 mono	11	3	2.7	3.8	13.0	15	27	38	47	62	72
NSCLC PDL1 present, no upper limit	PD1/PDL1 mono	12	4.8	3.4	7.1	20.8	10	18	26	33	45	55
NSCLC ALK+	crizotinib	2	7.6	7.6	7.6	32.9	6	12	17	22	32	40
NSCLC ALK+	Other ALK TKI	8	8	4.9	16.8	34.7	6	11	16	21	30	38
NSCLC EGFR-m	gefit or erlot	6	9	7.7	10.7	39.0	5	10	15	19	27	35
NSCLC EGFR-u	EGFR TKI	50	3.1	1.9	8.1	13.4	14	27	37	46	60	71
NSCLC EGFR-u	erlot combo	2	3.7	3.5	3.8	16.0	12	23	32	40	54	65
NSCLC EGFR WT	EGFR TKI	12	2.2	1.9	4.9	9.5	20	35	48	58	73	83

Continuation of Supplementary Online Table 3. Tumor type, therapy type, prior therapy for advanced disease, and the Proportion of remaining patients progressing since prior scan for different time intervals between scans

Tumor	Rx†	no. studies	median PFS $t_{1/2}$ months	lowest PFS $t_{1/2}$ months	highest PFS $t_{1/2}$ months	median PFS $t_{1/2}$ weeks	Time (weeks) from last scan					
							3 wk	6 wk	9 wk	12 wk	18 wk	24 wk
							Percent of remaining patients progressing since prior scan					
NSCLC	placebo/BSC	10	2	1.5	2.8	8.7	21	38	51	62	76	85
ovary	carboplatin	2	11.1	5.6	16.5	48.1	4	8	12	16	23	29
ovary	platinum combo	2	12	8.6	15.4	52.0	4	8	11	15	21	27
ovary	non-plat combo	1	6.6			28.6	7	14	20	25	35	44
ovary	paclitaxel	11	4	3.1	8.7	17.3	11	21	30	38	51	62
ovary	olaparib	1	10.4			45.1	5	9	13	17	24	31
ovary	bevacizumab	1	5.4			23.4	9	16	23	30	41	51
ovary	tamoxifen	1	2.2			9.5	20	35	48	58	73	83
pancreas	pemetrexed	1	2.1			9.1	20	37	50	60	75	84
pancreas neuroendocrine	sunitinib	1	11.8			51.1	4	8	11	15	22	28
prostate	abirat or enzalut or orteronel	5	8.4	6	22.8	36.4	6	11	16	20	29	37
prostate	small mol	7	6.1	3.4	9	26.4	8	15	21	27	38	47
prostate	docet+pred+/-bev	2	8	7	9	34.7	6	11	16	21	30	38
prostate	ipilimumab	2	5.6	4.3	6.8	24.3	8	16	23	29	40	50
prostate	placebo	6	4.3	3.3	4.9	18.6	11	20	28	36	49	59
renal	sorafenib	2	4.5	3.6	5.3	19.5	10	19	27	35	47	57
renal	mTOR inh	4	4.6	4.3	5	19.9	10	19	27	34	47	57
renal	Cabozantinib	1	7.4			32.1	6	12	18	23	32	40
renal	bevacizumab	2	4.4	3.3	5.5	19.1	10	20	28	35	48	58
renal	axitinib	1	3.7			16.0	12	23	32	40	54	65
renal	lenvatinib	1	8			34.7	6	11	16	21	30	38
renal	nivolumab	4	4.1	3.6	5.4	17.8	11	21	30	37	50	61
renal	placebo	5	2.8	2.2	3.8	12.1	16	29	40	50	64	75
sarcoma	Dacarbazine	1	2.3			10.0	19	34	47	57	71	81
sarcoma	trabectidin	1	4.1			17.8	11	21	30	37	50	61
sarcoma	pazopanib	1	4.2			18.2	11	20	29	37	50	60

Continuation of Supplementary Online Table 3. Tumor type, therapy type, prior therapy for advanced disease, and the Proportion of remaining patients progressing since prior scan for different time intervals between scans

Tumor	Rx†	no. studies	median PFS $t_{1/2}$ months	lowest PFS $t_{1/2}$ months	highest PFS $t_{1/2}$ months	median PFS $t_{1/2}$ weeks	Time (weeks) from last scan					
							3 wk	6 wk	9 wk	12 wk	18 wk	24 wk
							Percent of remaining patients progressing since prior scan					
SCLC	amrubicin	1	3.6			15.6	12	23	33	41	55	66
SCLC	topotecan	1	3.3			14.3	14	25	35	44	58	69
SCLC	nivolumab	1	2			8.7	21	38	51	62	76	85
thyroid	lenvatinib	1	17.6			76.3	3	5	8	10	15	20
thyroid	placebo	1	3.5			15.2	13	24	34	42	56	67
tumors MSI-H	PD1/PDL1 mono	1	19.3			83.6	2	5	7	9	14	18
urothelial	taxane or vinflunine	1	3.4			14.7	13	25	35	43	57	68
urothelial	PD1/PDL1 mono	5	3.1	1.7	3.9	13.4	14	27	37	46	60	71
**Both Rx naïve and pretreated patients included in study**												
breast	anthracycline	1	5.3			23.0	9	17	24	30	42	52
breast triple neg	cisplatin	1	2.2			9.5	20	35	48	58	73	83
breast	combinations	1	20.4			88.4	2	5	7	9	13	17
breast ER+	Fulvestrant	1	12.6			54.6	4	7	11	14	20	26
cervix	cisplatin	2	3.4	3.2	3.6	14.7	13	25	35	43	57	68
GIST	imatinib	5	23	19.8	26.1	99.7	2	4	6	8	12	15
melanoma	PD1/PDL1 mono	2	5.3	2.7	7.9	23.0	9	17	24	30	42	52
melanoma	ipilimumab	2	3.4	3.3	3.5	14.7	13	25	35	43	57	68
pancreas neuroendocrine	everolimus	1	16.9			73.2	3	6	8	11	16	20
renal	interferon	1	2.7			11.7	16	30	41	51	66	76
renal	sorafenib	1	9.6			41.6	5	10	14	18	26	33
renal	sunitinib	1	12.7			55.0	4	7	11	14	20	26
renal	tivozanib	1	12.4			53.7	4	7	11	14	21	27
renal	pazopanib	1	10.1			43.8	5	9	13	17	25	32
renal	bevacizumab	3	6.7	5.7	7.2	29.0	7	13	19	25	35	44
renal	placebo	1	4			17.3	11	21	30	38	51	62
sarcoma	sorafenib	1	3.4			14.7	13	25	35	43	57	68
**Maintenance therapy**												

Continuation of Supplementary Online Table 3. Tumor type, therapy type, prior therapy for advanced disease, and the Proportion of remaining patients progressing since prior scan for different time intervals between scans

Tumor	Rx†	no. studies	median PFS $t_{1/2}$ months	lowest PFS $t_{1/2}$ months	highest PFS $t_{1/2}$ months	median PFS $t_{1/2}$ weeks	Time (weeks) from last scan					
							3 wk	6 wk	9 wk	12 wk	18 wk	24 wk
							Percent of remaining patients progressing since prior scan					
breast	combination	1	7.3			31.6	6	12	18	23	33	41
breast	bevacizumab	1	5.3			23.0	9	17	24	30	42	52
breast	BSC	1	4.4			19.1	10	20	28	35	48	58
colon	bevacizumab	2	4.7	4.6	4.8	20.4	10	18	26	34	46	56
gastric	BSC	1	4.9			21.2	9	18	25	32	44	54
glioma	bevacizumab	1	3.9			16.9	12	22	31	39	52	63
NSCLC	pemetrexed	4	4.9	4.5	8.7	21.2	9	18	25	32	44	54
NSCLC	gemcitabine	2	3.8	3.8	3.8	16.5	12	22	32	40	53	64
NSCLC	bevacizumab	2	4	3.7	4.2	17.3	11	21	30	38	51	62
NSCLC	placebo/BSC	10	2.6	2.2	3.3	11.3	17	31	43	52	67	77
NSCLC EGFR-u	gefit or erlot	4	4.1	3.3	6.4	17.8	11	21	30	37	50	61
NSCLC EGFR-u	erlot+bev	1	5.1			22.1	9	17	25	31	43	53
NSCLC EGFR-u	vandetanib	1	3.2			13.9	14	26	36	45	59	70
NSCLC EGFR WT	erlotinib	1	2.8			12.1	16	29	40	50	64	75
ovary	placebo	1	4.7			20.4	10	18	26	34	46	56
sarcoma	ridaforolimus	1	4.4			19.1	10	20	28	35	48	58
sarcoma	placebo	1	3.3			14.3	14	25	35	44	58	69

† Abbreviations for tables	
abirat	abiraterone
ALK	anaplastic lymphoma kinase
ALK TKI	ALK tyrosine kinase inhibitor
bev	bevacizumab
breast ER+	breast estrogen receptor positive
breast HER2+	breast HER2 positive
breast triple neg	breast triple negative
BSC	best supportive care
carbo	carboplatin
cetux	cetuximab
chemo	chemotherapy
cisp	cisplatin
dabra	dabrafenib
dacarb	dacarbazine
docet	docetaxel
EGFR mono	EGFR monoclonal antibody
EGFR TKI	EGFR tyrosine kinase inhibitor
erlot	erlotinib
etop	etoposide
FOLFIRI	5-fluorouracil + folinic acid + irinotecan
FOLFOX	5-fluorouracil + folinic acid + oxaliplatin

Continuation of Supplementary Online Table 3. Tumor type, therapy type, prior therapy for advanced disease, and the Proportion of remaining patients progressing since prior scan for different time intervals between scans	
Gem	gemcitabine
GI	Gastrointestinal
GIST	gastrointestinal stromal tumor
IFN	interferon
IL2	interleukin-2
ipi	ipilimumab
LCI	lower 95% confidence interval
mo.	months
MSI-H	high microsatellite instability
mTOR inh	mTOR inhibitor
nivol	nivolumab
NSCLC	non-small cell lung cancer
NSCLC ALK+	NSCLC positive for ALK fusion gene
NSCLC EGFR WT	NSCLC with wild type EGFR
NSCLC EGFR-m	NSCLC positive for mutation in epidermal growth factor receptor
NSCLC EGFR-u	NSCLC unselected for EGFR mutation (mixture of patients with and without mutations)
oxal	oxaliplatin
panitum	panitumumab
PCV	procarbazine + CCNU + vincristine
PD1/PDL1	PD1/PDL1 monoclonal antibodies
pembro	pembrolizumab
Plat	cisplatin or carboplatin
pred	prednisone
Rx	treatment
SCLC	small cell lung cancer
small mol	Other small molecules
temoz	temozolamide
TKI	tyrosine kinase inhibitor
TMB high	high tumor mutation burden
UCI	upper 95% confidence interval
vemur	vemurafenib
vinbl	vinblastine
XELOX	capecitabine + folinic acid + oxaliplatin
XRT	radiotherapy
